# Supplementary material for: Conformational tuning improves the stability of spirocyclic nitroxides with long paramagnetic relaxation times
Source: Commun Chem. 2023 Jun 5;6:111. doi: 10.1038/s42004-023-00912-7 (PMC10241799; doi:10.1038/s42004-023-00912-7)
Supplement: Supplementary file 2 — Supplementary Information [file 42004_2023_912_MOESM2_ESM.pdf]

Supplementary Information

Conformational tuning improves the stability of spirocyclic nitroxides with long paramagnetic relaxation times

Mateusz P. Sowiński,[a] Sahil Gahlawat,[a,b] Bjarte A. Lund,[a†] Anna-Luisa Warnke,[a†] Kathrin H. Hopmann,[a] Janet E. Lovett,[c] Marius M. Haugland [a]*

[†] These authors contributed equally
** e-mail: marius.m.haugland@uit.no*

[a] Department of Chemistry, UiT The Arctic University of Norway, 9037 Tromsø, Norway

[b] Hylleraas Center for Quantum Molecular Sciences, UiT The Arctic University of Norway, 9037 Tromsø, Norway

[c] SUPA, School of Physics and Astronomy and BSRC, University of St Andrews, North Haugh, St Andrews, KY16 9SS, UK

1. Supplementary Methods - General procedures and materials

All reagents, obtained from Acros, Alfa, Sigma- Aldrich, TCI, and VWR were used directly as supplied unless otherwise noted. Phosphate-buffered saline (PBS) (10×, pH 7.4, Gibco™ product line, 1551.72 mM NaCl, 29.66 mM Na_2HPO_4, and 10.58 mM KH_2PO_4) was purchased from Thermo Fisher Scientific. Anhydrous solvents were dried by pre-storing over activated 4 Å molecular sieves. Thin layer chromatography (TLC) was used to monitor reaction progress. TLC analysis was performed on pre-coated aluminium-based plates (TLC Silica gel 60 F_{254}, Supelco) and plates were developed under UV irradiation (254 nm) or with $KMnO_4$ staining and subsequent heating. Column chromatography was performed with silica gel (Silica gel 60, irregular 40–63 µm for flash chromatography, VWR Chemicals). ^{1}H Nuclear magnetic resonance (NMR) spectra were recorded at ambient probe temperatures on the Bruker 9.4 Tesla Avance III HD system equipped with a SmartProbe (broad band) operating at 400 MHz. Spectra were referenced to residual protons in $CDCl_3$ (δ 7.26) or benzene (δ 7.36) for nitroxides reduced with phenylhydrazine. Chemical shifts (δ) are reported as parts per million (ppm). Coupling constants (*J*) are given in Hz to an accuracy of 0.1 Hz. Assignment of ^{1}H resonances were determined based on unambiguous chemical shift, coupling patterns, by analysis of 2D NMR (COSY, HSQC and/or HMBC) and/or by analogy to fully interpreted spectra of closely structurally related compounds. ^{13}C NMR spectra (^{1}H decoupled) were recorded at ambient probe temperatures on the instrument mentioned above, operating at 101 MHz, and referenced to the $CDCl_3$ (δ 77.16). High-resolution mass spectra (HRMS) were recorded at the Faculty of Biosciences, Fisheries and Economics, UiT The Arctic University of Norway, using high-resolution Agilent 6540B quadrupole time-of-flight (Q-ToF) mass spectrometer with a dual electrospray ionization (ESI) source, coupled to a Agilent 1290 Infinity UHPLC system, controlled by MassHunter software. IR spectra were obtained on an Agilent Technologies Cary 630 FTIR 318 spectrometer and absorptions are reported in wavenumber (cm^{-1}). Melting points were measured using Stuart SMP50 automatic melting point detector. Diastereoisomers were separated using a Teledyne ISCO CombiFlash EZ Prep system.

2. Crystallography data

Crystals of compounds **5**, **8** and *cis*,*cis*-**9** were grown from diethyl ether as solvent by the slow evaporation method. Crystals were glued to glass fibres and mounted on the goniometer. Data were collected using a D8 Venture system with a Cu-anode d (λ = 1.54178 Å). Integration of the data was done using Bruker SAINT software. The structures were solved by direct methods using SHELXT (Sheldrick 2014). Full-matrix least square refinement was done on the amplitudes using SHELXL (Sheldrick, 2018). Figures were prepared in PyMOL (Schrödinger). Ellipsoids are presented below. The crystal structures have been deposited and can be accessed in the Cambridge Crystallographic Data Centre (CCDC) with the following deposition numbers **5:** 2214626, *trans*-**8:** 2214625 and *cis,cis*-**9:** 2214627.

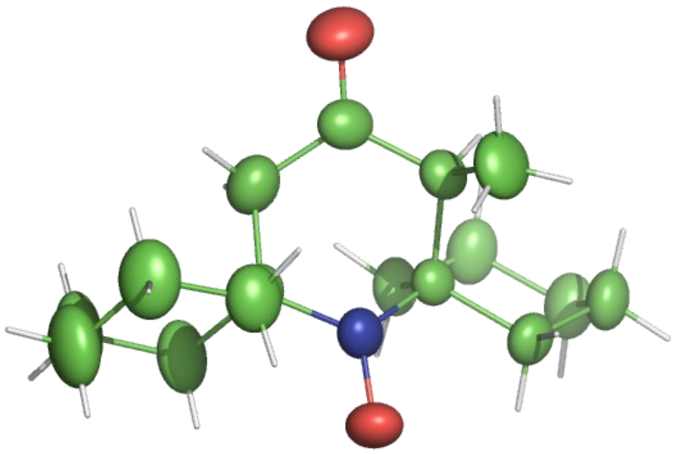

Figure S1. *Ellipsoids extracted from the X-ray analysis of the solid-state structure of **5**.*

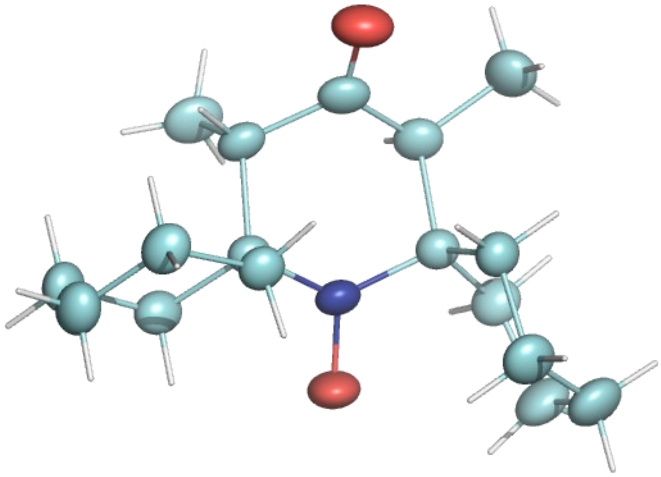

Figure S2. *Ellipsoids extracted from the X-ray analysis of the solid-state structure of trans-**8**.*

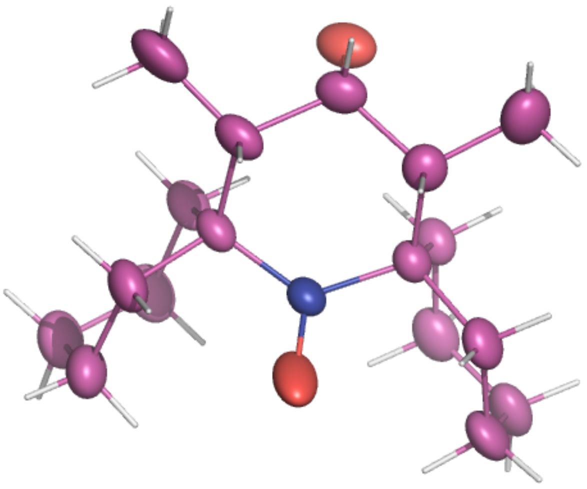

Figure S3. *Ellipsoids extracted from the X-ray analysis of the solid-state structure of cis,cis-**9**.*

3. Synthesis and characterization of nitroxides and intermediates

3.1. 7-Azadispiro[5.1.5^8.3^6]hexadecan-15-one (3)

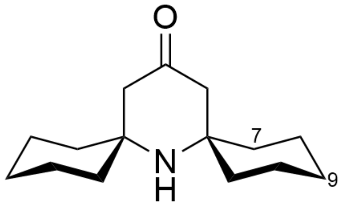

2,2,6,6-Tetramethyl-4-piperidone (8.00 g, 49.0 mmol, 1.0 equiv.), NH_4Cl (14.0 g, 261 mmol, 5.4 equiv.) and cyclohexanone (16.5 mL, 159 mmol, 3.3 equiv.) were dissolved in DMSO (32 mL) at room temperature under Ar. Triton B (40% in methanol, 11.0 mL) was added dropwise over stirring at room temperature, and the reaction mixture was stirred at 50 °C for 48 h. Water (60 mL) was added and the mixture was stirred for further 30 min. The solution was diluted with water (120 mL), acidified with conc. HCl, and extracted with diethyl ether (2 × 100 mL). The ether washings were discarded, and the aqueous DMSO solution was adjusted to pH > 9 with NaOH (6 M). The mixture was extracted with ethyl acetate (3 × 150 mL) and the combined extracts were dried over Na_2SO_4. The solvent was removed, and the residue purified by column chromatography (ethyl acetate / petroleum ether (2:8)). Subsequent recrystallization (EtOH) provided **3** as white crystals (4.54 g, 19.3 mmol, 39%).

R_f 0.38 (ethyl acetate / *n*-pentane (3:7)); **^{1}H NMR** (400 MHz, $CDCl_3$) δ 2.31 (4H, s, H3, H5), 1.72–1.30 (20H, m, H7-H11), 1.05 (1H, s, NH); **^{13}C NMR** (101 MHz, $CDCl_3$) δ 211.3, 56.6, 52.2, 40.6, 25.5, 22.2.

Data are consistent with literature values.[1]

3.2. 14-Methyl-7-azadispiro[5.1.5^{8}.3^{6}]hexadecan-15-one (4)

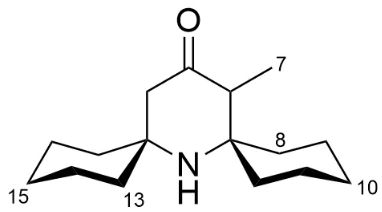

A dried vial equipped with a magnetic stir bar and a rubber serum cap was charged with anhydrous THF (2.5 mL) under a nitrogen atmosphere. Diisopropylamine (108 µL, 0.765 mmol, 1.2 equiv.) was added via syringe at room temperature and the solution was cooled to 0 °C. *n*-Butyllithium solution (333 µL, 0.765 mmol, 1.20 equiv., 2.3 M in hexanes) was added at 0 °C and the solution stirred at 0 °C for 15 min and then cooled to -78 °C. Ketoamine **3** (150 mg, 0.637 mmol, 1.0 equiv.) was dissolved in anhydrous THF (1 mL), added dropwise to freshly prepared LDA at -78 °C and stirred for 30 min. Methyl iodide (60.0 µL, 0.956 mmol, 1.5 equiv.) was added dropwise to the mixture and the cooling bath was allowed to warm up to room temperature and stirred for 48 h. Quenched by addition of sat. aqueous NH_4Cl solution (1 mL), diluted with sat. aqueous $NaHCO_3$ solution (10 mL) and extracted with diethyl ether (3 x 10 mL). The combined organic phases were dried over Na_2SO_4 and concentrated *in vacuo*. The residue purified by column chromatography (ethyl acetate / *n*-pentane (1:9)) provided **4** as white crystals (70 mg, 0.28 mmol, 44%).

R_f 0.22 (ethyl acetate / *n*-pentane (1:9)); **m.p.** 97-99 °C; **IR** (solid, v_{max} / cm^{-1}) 2925, 2854, 1696, 1443, 1264, 1007, 717; **^{1}H NMR** (400 MHz, $CDCl_3$) δ 2.43 (1H, d, *J* = 13.3 Hz, H3a), 2.32 (1H, qd, *J* = 6.9, 1.2 Hz, H5), 2.17 (1H, dd, *J* = 13.3, 1.2 Hz, H3b), 1.75–1.10 (20H, m, H8-12, H13-17), 1.05 (1H, s, NH), 1.00 (3H, d, *J* = 6.9 Hz, H7); **^{13}C NMR** (101 MHz, $CDCl_3$) δ 214.0, 59.6, 57.0, 54.8, 50.4, 42.2, 40.1, 39.9, 32.9, 26.0, 25.9, 22.7, 22.6, 22.1, 21.9, 10.4; **HRMS (EI+)** calc. for $C_{16}H_{27}NO$ $[M]^+$ 250.2126, found 250.2167.

3.3. 14-Methyl-7-azadispiro[5.1.5^{8}.3^{6}]hexadecan-15-one-1-oxyl (5)

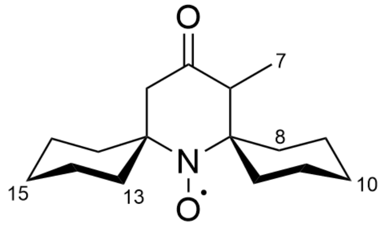

Ketoamine **4** (273 mg, 1.1 mmol, 1.0 equiv.) was dissolved in CH_2Cl_2 (2 mL) and the solution was cooled to 0 °C. A solution of *m*-CPBA (540 mg, 2.19 mmol, 2.0 equiv.) in CH_2Cl_2 (3.5 mL) was added dropwise at 0 °C, and the reaction mixture was stirred at room temperature for 3 h. The mixture was washed with 10% aqueous Na_2CO_3 (5 mL), and the aqueous phase was

back-extracted with CH_2Cl_2 (3 × 10 mL). The combined organic phases were dried over Na_2SO_4 and the solvent was removed *in vacuo*. The residue was purified by column chromatography (diethyl ether / *n*-pentane (1:9)) to afford nitroxide **5** as a red-orange solid (243 mg, 0.919 mmol, 84%). Prior to NMR analysis, radical **5** was reduced *in situ* to the corresponding hydroxylamine by addition of phenylhydrazine directly to the NMR tube.

R_f 0.16 (diethyl ether / *n*-pentane (1:9)); **m.p.** 58-62 °C; **IR** (solid, ν_{max} / cm^{-1}) 2940, 2921, 2866, 1707, 1458, 1231); **^{1}H NMR** (400 MHz, $CDCl_3$) δ 2.84 (1H, qd, *J* = 7.0, 1.7 Hz, H3a), 2.67 (1H, dd, *J* = 13.4, 1.8 Hz, H5), 2.51 (1H, d, *J* = 13.4 Hz, H3b), 2.14–1.18 (20H, m, H8-H12, H13-H17), 1.13 (3H, d, *J* = 7.0 Hz, H7); **^{13}C NMR** (101 MHz, $CDCl_3$) δ 213.1, 48.6, 44.3, 37.8, 33.1, 32.0, 25.7, 25.5, 22.8, 22.8, 22.7, 22.2, 11.7; **HRMS (ESI+)** calc. for $C_{16}H_{26}NO_2$ $[M+2H]^+$ 266.2031, found 266.2116.

3.4. 14,16-Dimethyl-7-azadispiro[5.1.5^8.3^6]hexadecan-15-one-1-oxyl (7)

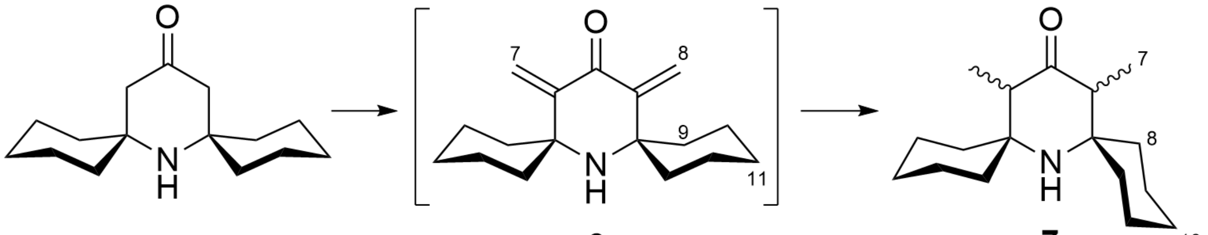

6 **7**

Preparation and isolation of 6

Ketoamine **4** (90 mg, 0.38 mmol, 1.0 equiv.) and NaOH (148 mg, 3.70 mmol, 9.7 equiv.) were dissolved in THF (20 mL) and water (60 mL) at room temperature and stirred for 15 min. Then, formaldehyde solution (0.41 mL, 5.5 mmol, 14.0 equiv., 37% wt. in water) was added and stirred at room temperature for 3 h. The mixture was extracted with diethyl ether (3 × 50 mL), dried over Na_2SO_4 and concentrated *in vacuo*. The desired product **6** was isolated by column chromatography (diethyl ether / *n*-pentane (1:9)) as a white solid (55 mg, 0.31 mmol; 82%).

R_f 0.28 (diethyl ether / *n*-pentane (1:9)); **^{1}H NMR** (400 MHz, $CDCl_3$) δ 6.06 (2H, d, *J* = 1.1 Hz, H7a, H8a), 5.33 (2H, d, *J* = 1.2 Hz, H7b, H8b), 1.82–0.79 (20H, m, H9-H13); **^{13}C NMR** (101 MHz, $CDCl_3$) δ 191.5, 153.3, 118.6, 54.7, 38.8, 25.9, 21.9.

Preparation of 7 without isolation of 6

Ketoamine **4** (756 mg, 3.21 mmol, 1.0 equiv.) and NaOH (1.00 g, 22.6 mmol, 7.0 equiv.) were dissolved in THF (65 mL) and water (65 mL) and room temperature and stirred for 15 min. Then, formaldehyde solution (3.3 mL, 45.0 mmol, 14.0 equiv., 37% wt. in water) was added

and stirred at room temperature for 3 h. The mixture was extracted with diethyl ether (3 × 50 mL) and dried over Na_2SO_4 and concentrated *in vacuo*. The residue was immediately reduced.

Reduction was performed in a Parr reactor. Residue containing **6** (1.056 g) from previous step, was dissolved in ethyl acetate (25 mL) and placed in 50 mL Parr reactor. Pd on charcoal (10 wt%, 90 mg) was weighed under nitrogen and placed in Parr reactor equipped with a seal and a stirred at room temperature. The reactor was flushed with hydrogen three times. The reaction was stirred for 18 h at 5 bar hydrogen gas pressure. Then the mixture was filtered over cellite and solvent was evaporated. Product **7** was isolated by column chromatography (diethyl ether / *n*-pentane (1:9)) as a white solid (593 mg, 2.25 mmol, 70% over two steps).

R_f 0.42, 0.40 (diethyl ether / *n*-pentane (3:7)); ***dr*** 1:1; **IR** (solid, ν_{max} / cm^{-1}) 2929, 2854, 1696, 1454, 1365, 1186, 1011; **^{1}H NMR** (400 MHz, $CDCl_3$) δ 2.47 (2H, q, *J* = 6.9 Hz, H3, H5), 2.28 (2H, q, J = 6.2 Hz, H3', H5'), 1.85–1.01 (30H, m, H8-H12, H8'-H12'), 0.99 (6H, d, *J* = 7.0 Hz, H7), 0.95 (6H, d, *J* = 6.8 Hz, H7').; **^{13}C NMR** (101 MHz, $CDCl_3$) δ 214.0, 62.1, 57.2, 41.7, 29.7, 26.3, 22.3, 9.6; **HRMS** (ESI+) calc. for $C_{17}H_{29}NO$ $[M]^+$ 264.2283, found 264.2325.

3.5. 14,16-Dimethyl-7-azadispiro[5.1.5^8.3^6]hexadecan-15-one-1-oxyl (8)

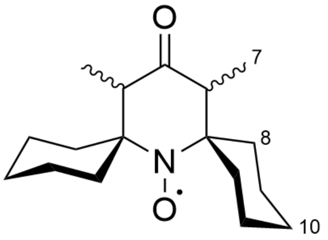

Ketoamine **7** (222 mg, 0.843 mmol, 1.0 equiv.) was dissolved in CH_2Cl_2 (4 mL) and K_2CO_3 (300 mg, 2.17 mmol, 2.6 equiv.) was added at room temperature. The suspension was cooled to 0 °C, a solution of *m*-CPBA (378 mg, 1.69 mmol, 2.0 equiv.) in CH_2Cl_2 (2 mL) was added dropwise and the reaction mixture was stirred at room temperature for 17 h. The mixture was washed with 10% aqueous Na_2CO_3 (10 mL), and the aqueous phase was back-extracted with CH_2Cl_2 (3 × 10 mL). The combined organic phases were dried over Na_2SO_4 and the solvent was removed *in vacuo*. The residue was purified by column chromatography (diethyl ether / *n*-pentane (2:8)) to afford nitroxide **8** as a red-orange solid (217 mg, 0.779 mmol, 92%). Prior to NMR analysis, radical **8** was reduced *in situ* to the corresponding hydroxylamine by addition of phenylhydrazine directly to the NMR tube.

R_f 0.53, 0.44 (diethyl ether / *n*-pentane (3:7)); ***dr*** 1:1; **IR** (solid, ν_{max} / cm^{-1}) 2929, 2858, 1711, 1451, 1357, 911; **^{1}H NMR** (400 MHz, $CDCl_3$) δ 2.94 (4H, q, *J* = 6.9 Hz, H3, H3', H5, H5'), 2.38–1.25 (40H, m, H8-H12, H8'-H12'), 1.17 (6H, d, *J* = 7.1 Hz, H7), 1.00 (6H, d, *J* = 6.8 Hz, H7').;

^{13}C NMR (101 MHz, $CDCl_3$) δ 213.9, 49.4, 37.1, 36.5, 32.4, 30.9, 26.6, 25.9, 22.9, 22.7, 22.7, 22.5, 12.1, 9.3; **HRMS** (ESI+) calc. for $C_{17}H_{28}NO_2$ $[M+2H]^+$ 280.2187, found 280.2277.

3.6. 14,16-Dimethyl-7-azadispiro[5.1.5^8.3^6]hexadecan-15-ol-1-oxyl (9)

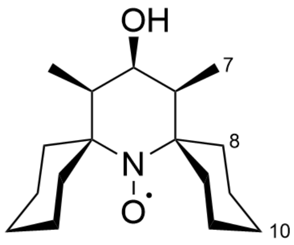

Nitroxide **8** (97 mg, 0.348 mmol, 1.0 equiv.) was dissolved in ethanol (3 mL). Then, $NaBH_4$ (15 mg, 0.381 mmol, 1.1 equiv.) was added carefully at room temperature over stirring. The reaction mixture was stirred at room temperature for 18 h, before being saturated with sat. aqueous NaCl solution. The mixture was extracted with diethyl ether (3 × 10 mL) and the combined organic layers were dried over Na_2SO_4. The solvent was removed *in vacuo* to afford nitroxide **9** as orange-red crystals (*dr* 1:1, 98 mg, 0.369 mmol, 99%). The diastereoisomers could be partially separated by column chromatography (diethyl ether / *n*-pentane (3:7)) to give pure *cis,cis*-isomer for further investigations. Prior to NMR analysis, radical **9** was reduced *in situ* to the corresponding hydroxylamine by addition of phenylhydrazine directly to the NMR tube. However, significant line-broadening was still apparent, especially in the ^{13}C NMR spectrum of **9**. Thus, we do not report ^{13}C resonances for this compound.

Characterization data for pure cis,cis-isomer:

R_f 0.22 (diethyl ether / *n*-pentane (3:7)); **m.p.** 156-159 °C; **IR** (solid, ν_{max} / cm^{-1}) 3495, 2925, 2858, 1458, 1037, 739; **^{1}H NMR** (400 MHz, $CDCl_3$) δ 3.78 (1H, dd, J = 10.0, 4.4 Hz, H4), 2.05–1.24 (20H, m, H8-H12), 1.04-1.07 (6H, m, H7); **HRMS** (ESI+) calc. for $C_{17}H_{30}NO_2$ $[M+2H]^+$ 282.2344, found 282.2426.

3.7. 7-Azadispiro[5.1.5^8.3^6]hexadecan-15-one-1-oxyl (10)

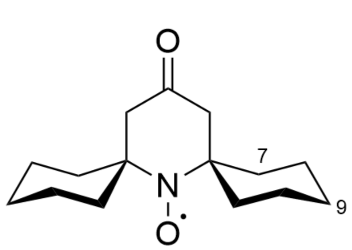

Ketoamine **3** (201 mg, 0.854 mmol, 1.0 equiv.) was dissolved in CH_2Cl_2 (4 mL) and K_2CO_3 (300 mg, 2.17 mmol, 2.5 equiv.) was added at room temperature. The suspension was cooled

to 0 °C and stirred. A solution of *m*-CPBA (420 mg, 1.70 mmol, 2.0 equiv.) in CH_2Cl_2 (1.7 mL) was added dropwise, and the reaction mixture was stirred at room temperature for 3 h. The mixture was washed with 10% aqueous Na_2CO_3 (10 mL), and the aqueous phase was back-extracted with CH_2Cl_2 (3 × 10 mL). The combined organic phases were dried over Na_2SO_4 and the solvent was removed *in vacuo*. The residue was purified by column chromatography (ethyl acetate / *n*-pentane (2:8)) to afford nitroxide **10** as a red-orange solid (180 mg, 0.719 mmol, 84%). Prior to NMR analysis, radical **10** was reduced *in situ* to the corresponding hydroxylamine by addition of phenylhydrazine directly to the NMR tube.

R_f 0.42 (ethyl acetate / *n*-pentane (2:8)); **^{1}H NMR** (400 MHz, $CDCl_3$) δ 2.53 (4H, s, H3, H5), 2.06–1.08 (20H, m, H7-H11); **^{13}C NMR** (101 MHz, $CDCl_3$) δ 47.6, 35.1, 25.5, 23.6, 22.9.

Data are consistent with literature values.[2]

3.8. 7-Azadispiro[5.1.5^8.3^6]hexadecan-15-ol-1-oxyl (11)

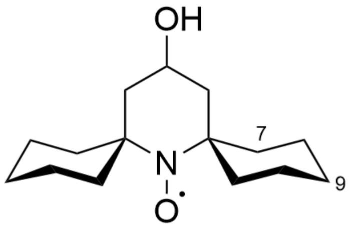

Nitroxide **10** (170 mg, 0.679 mmol, 1.0 equiv.) was dissolved in ethanol (6 mL). Then, $NaBH_4$ (29 mg, 0.747 mmol, 1.1 equiv.) was added carefully at room temperature while stirring. The reaction mixture was stirred at room temperature for 2 h, before being saturated with sat. aqueous NaCl solution. The mixture was extracted with diethyl ether (3 × 10 mL) and the combined organic layers were dried over Na_2SO_4. The solvent was removed *in vacuo* to afford nitroxide **11** as orange-red crystals (151 mg, 0.598 mmol, 88%). Prior to NMR analysis, radical **11** was reduced *in situ* to the corresponding hydroxylamine by addition of phenylhydrazine directly to the NMR tube.

R_f 0.42 (ethyl acetate / *n*-pentane (3:7)); **^{1}H NMR** (400 MHz, $CDCl_3$) δ 3.94–3.87 (1H, m, OH), 2.47 (1H, d, J = 11.1 Hz, H4), 2.07-1.90 (4H, m, H3, H5), 1.77–1.01 (20H, m, H7-H11).; **^{13}C NMR** (101 MHz, $CDCl_3$) δ 62.5, 41.6, 40.1, 25.4, 23.1, 22.7.

Data are consistent with literature values.[2]

3.9. 2,2,6,6-Tetramethylpiperidin-4-one-1-oxyl (14)

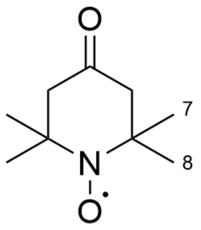

2,2,6,6-Tetramethyl-4-piperidone (215 mg, 1.32 mmol, 1.0 equiv.) was dissolved in CH_2Cl_2 (10 mL) and K_2CO_3 (460 mg, 3.33 mmol, 2.5 equiv.) was added. The suspension was cooled to 0 °C, a solution of *m*-CPBA (655 mg, 2.66 mmol, 2.0 equiv.) in CH_2Cl_2 (2.4 mL) was added dropwise, and the reaction mixture was stirred at room temperature for 2 h. The mixture was washed with 10% aqueous Na_2CO_3 (10 mL), and the aqueous phase was back-extracted with CH_2Cl_2 (3 × 10 mL). The combined organic phases were dried over Na_2SO_4 and the solvent was removed *in vacuo*. The residue was purified by column chromatography (diethyl ether / *n*-pentane (3:7)) to afford nitroxide **14** as a red-orange solid (173 mg, 1.02 mmol, 77%). Prior to NMR analysis, radical **14** was reduced *in situ* to the corresponding hydroxylamine by addition of phenylhydrazine directly to the NMR tube.

R_f 0.14 (diethyl ether / *n*-pentane (3:7)); **^{1}H NMR** (400 MHz, $CDCl_3$) δ 2.41-2.46 (4H, m, H3, H5), 1.25-1.26 (12H, m, H7, H8'); **^{13}C NMR** (101 MHz, $CDCl_3$) δ 47.4, 38.0, 26.1, 25.7.

Data are consistent with literature values.[2]

3.10. 2,2,6,6-Tetramethylpiperidin-4-ol-1-oxyl (12)

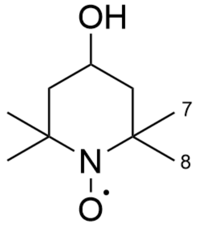

Nitroxide **14** (173 mg, 1.02 mmol, 1.0 equiv.) was dissolved in ethanol (8.5 mL). Then, $NaBH_4$ (53 mg, 1.35 mmol, 1.3 equiv.) was added carefully at room temperature while stirring. The reaction mixture was stirred at room temperature for 2 h, before being saturated with sat. aqueous NaCl solution. The mixture was extracted with diethyl ether (3 × 10 mL) and the combined organic layers were dried over Na_2SO_4. The solvent was removed *in vacuo* to afford nitroxide **12** as orange-red crystals (173 mg, 1.00 mmol, 99%). Prior to NMR analysis, radical **12** was reduced *in situ* to the corresponding hydroxylamine by addition of phenylhydrazine directly to the NMR tube.

R_f 0.26 (ethyl acetate / *n*-pentane (3:7)); **^{1}H NMR** (400 MHz, $CDCl_3$) δ 4.00 (1H, tt, J = 11.4, 4.3 Hz, H4), 1.98–1.91 (2H, m, H3a, H5a), 1.67-1.61 (2H, m, H3b, H5b), 1.30 (6H, s, H7, H7'), 1.21 (6H, s, H8, H8'); **^{13}C NMR** (101 MHz, $CDCl_3$) δ 62.9, 47.4, 31.4, 20.7.

Data are consistent with literature values.[2]

3.11. 3,11-Dithia-7-azadispiro[5.1.5^8.3^6]hexadecan-15-one (15)

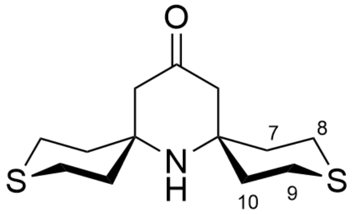

2,2,6,6-Tetramethyl-4-piperidone (1.97 g, 12.1 mmol, 1.0 equiv.), NH_4Cl (3.25 g, 60.8 mmol, 5.0 equiv.) and Tetrahydrothiopyran-4-one (4.21 g, 36.2 mmol, 3.0 equiv.) were dissolved in DMSO (8 mL) under Nitrogen. Triton B (40% in methanol, 2.7 mL) was added dropwise over stirring at room temperature, and the reaction mixture was stirred at 50 °C for 48 h. Water (10 mL) was added and the mixture was stirred for further 30 min. The solution was diluted with water (20 mL), acidified with conc. HCl, and extracted with diethyl ether (2 × 20 mL). The ether washings were discarded, and the aqueous DMSO solution was adjusted to pH > 9 with NaOH (6 M). The mixture was extracted with ethyl acetate (3 × 30 mL) and the combined extracts were dried over Na_2SO_4. The solvent was removed, and the residue purified by column chromatography (ethyl acetate / *n*-heptane (1:1)). Subsequent recrystallization (EtOH) provided **15** as pale yellow crystals (836 mg, 3.08 mmol, 26%).

R_f 0.34 (ethyl acetate / *n*-heptane (1:1)); **^{1}H NMR** (400 MHz, $CDCl_3$) δ 2.92 (4H, ddd, *J* = 13.2, 9.6, 3.1 Hz, H8, H8'), 2.50-2.43 (4H, m, H9, H9'), 2.28 (4H, s, H3, H5), 1.95–1.73 (8H, m, H7, H10), 0.82 (1H, s, NH); **^{13}C NMR** (101 MHz, $CDCl_3$) δ 209.2, 55.9, 53.2, 41.6, 24.2.

Data are consistent with literature values.[1]

3.12. 2,2,6,6-Tetraethylpiperidin-4-ol (16)

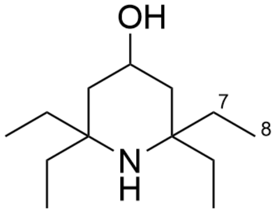

Ketoamine **15** (200 mg, 0.737 mmol, 1.0 equiv..) was dissolved in EtOH (6 mL) and treated with Raney®-Nickel (12 mL, 2800, slurry in water) at room temperature. The reaction mixture was heated at 65 °C for 20 h. After cooling to room temperature, the mixture was carefully filtered under a Nitrogen-flow through celite. The solvent of the filtrate was removed *in vacuo* to obtain the desired product (94.0 mg, 441 mmol, 60%) as a white solid.

R*f* 0.30 (ethyl acetate / *n*-pentane (2:8)); **^{1}H NMR** (400 MHz, $CDCl_3$) δ 4.02-3.96 (1H, m, H4), 1.86 (1H, d, *J* = 10.3 Hz, OH), 1.66–0.89 (12H, m, H3, H5, H7), 0.84-0.78 (12H, m, H8), 0.45 (1H, s, NH); **^{13}C NMR** (101 MHz, $CDCl_3$) δ 64.9, 56.0, 44.7, 34.8, 30.2, 8.6, 7.7.

Data are consistent with literature values.[3]

3.13. 2,2,6,6-Tetraethylpiperidin-4-ol-1-oxyl (13)

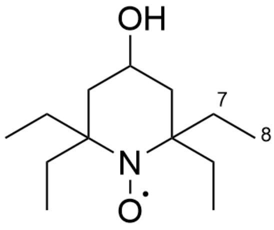

Aminoalcohol **16** (77 mg, 0.361 mmol, 1.0 equiv.) was dissolved in CH_2Cl_2 (1.5 mL) and K_2CO_3 (127 mg, 0.919 mmol, 2.6 equiv.) was added at room temperature. The suspension was cooled to 0 °C, a solution of *m*-CPBA (165 mg, 0.736 mmol, 2.0 equiv.) in CH_2Cl_2 (1.0 mL) was added dropwise, and the reaction mixture was stirred at room temperature for 24 h. The mixture was washed with 10% aqueous Na_2CO_3 (10 mL), and the aqueous phase was back-extracted with CH_2Cl_2 (3 × 10 mL). The combined organic phases were dried over Na_2SO_4 and the solvent was removed *in vacuo*. The residue was purified by column chromatography (ethyl acetate / *n*-heptane (4:6)) to afford nitroxide **13** as an orange solid (47 mg, 0.206 mmol, 57%). Prior to NMR analysis, radical **13** was reduced *in situ* to the corresponding hydroxylamine by addition of phenylhydrazine directly to the NMR tube.

R*f* 0.32 (ethyl acetate / *n*-heptane (4:6)); **^{1}H NMR** (400 MHz, $CDCl_3$) δ 3.99-3.92 (1H, m, H4), 2.01–1.25 (12H, m, H3, H5, H7), 0.86 (12H, t, *J* = 7.5 Hz, H8); **^{13}C NMR** (101 MHz, $CDCl_3$) δ 63.2, 39.1, 30.0, 26.6, 9.7, 8.1.

Data are consistent with literature values.[3]

4. EPR spectroscopy

4.1. CW spectra of nitroxides

Room temperature CW EPR measurements were acquired at the School of Physics and Astronomy, University of St Andrews, using an X-band Affirmo microESR Benchtop EPR Spectrometer (now marketed as the Bruker microESR).

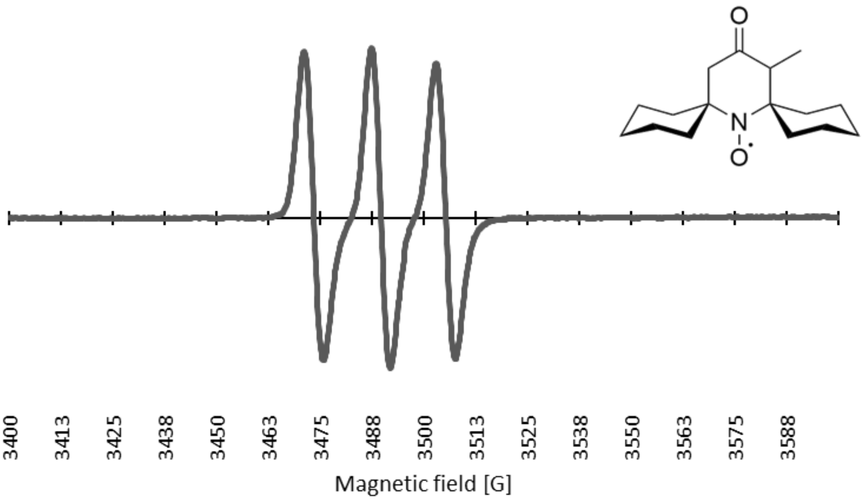

Figure S4. *Room temperature X-band (9.805 GHz) EPR spectrum of nitroxide **5** (2 mM in PBS buffer/DMSO 1:1 v/v).*

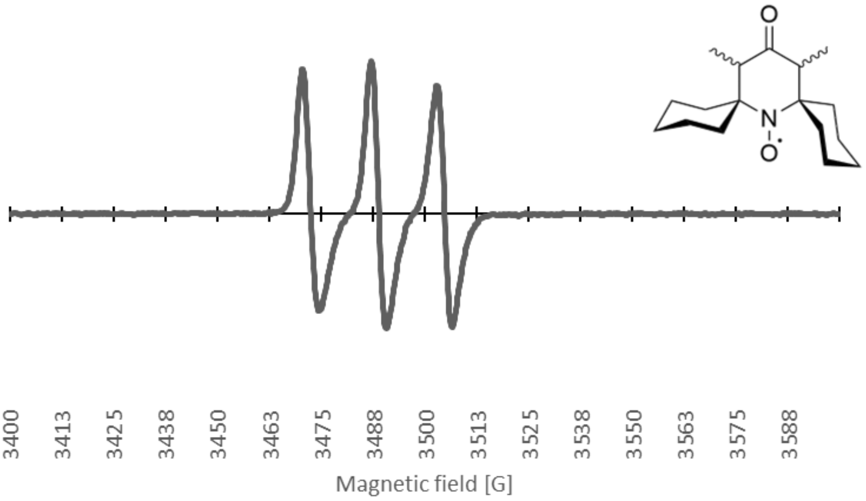

Figure S5. *Room temperature X-band (9.800 GHz) EPR spectrum of nitroxide **8** (2 mM in PBS buffer/DMSO 1:1 v/v).*

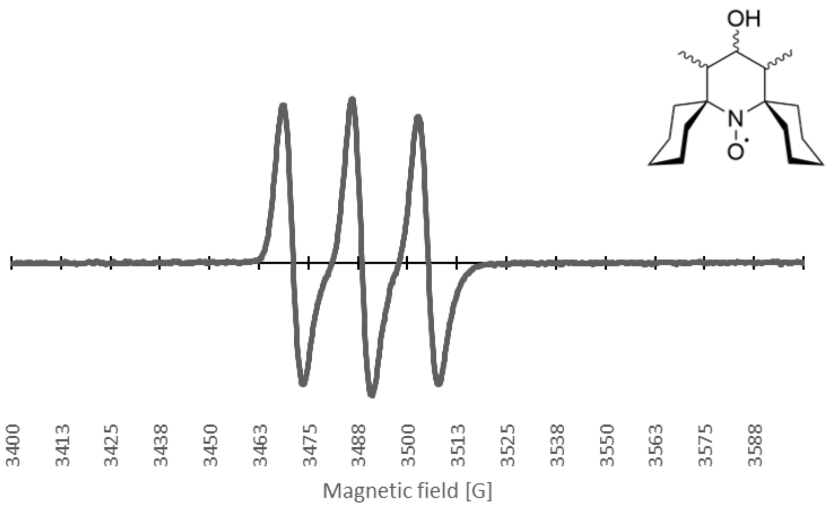

Figure S6. *Room temperature X-band (9.805 GHz) EPR spectrum of nitroxide cis,cis-**9** (2 mM in PBS buffer/DMSO 1:1 v/v).*

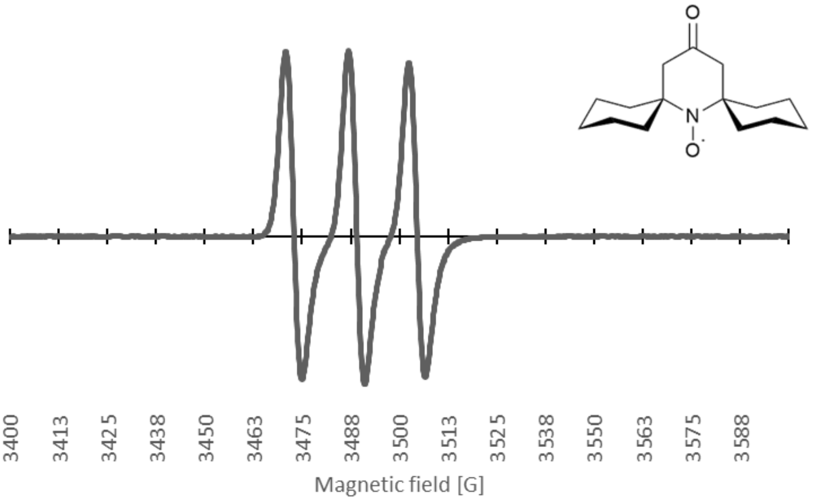

Figure S7. *Room temperature X-band (9.803 GHz) EPR spectrum of nitroxide **10** (2 mM in PBS buffer/DMSO 1:1*

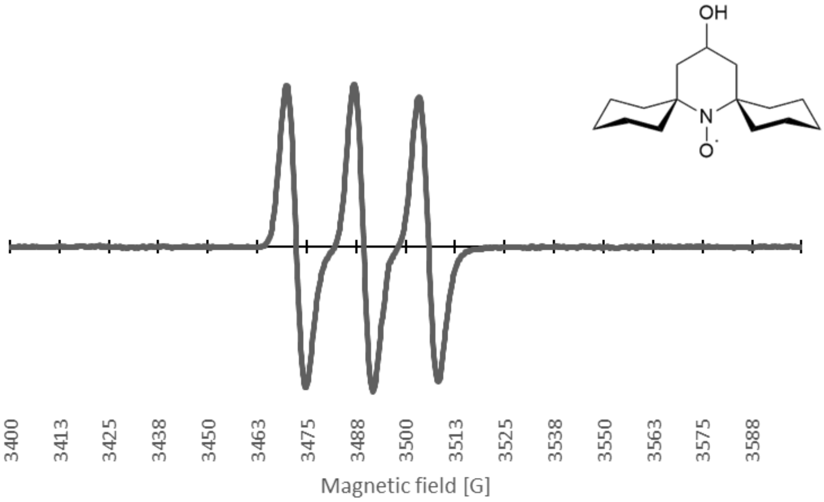

v/v).

Figure S8. *Room temperature X-band (9.803 GHz) EPR spectrum of nitroxide **11** (2 mM in PBS buffer/DMSO 1:1 v/v).*

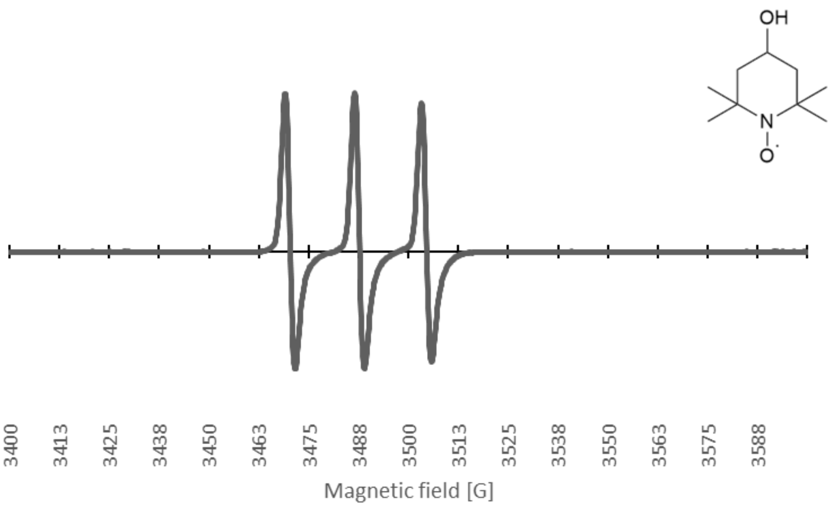

Figure S9. *Room temperature X-band (9.803 GHz) EPR spectrum of nitroxide **12** (2 mM in PBS buffer/DMSO 1:1 v/v).*

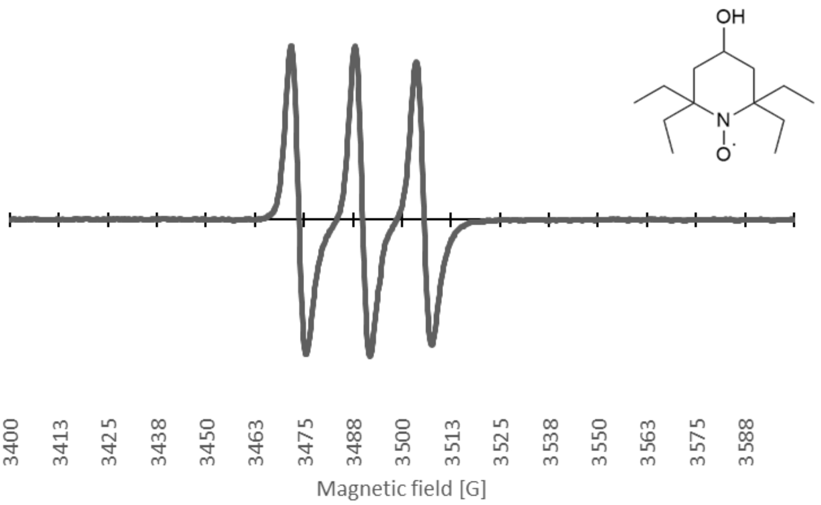

Figure S10. *Room temperature X-band (9.805 GHz) EPR spectrum of nitroxide **13** (2 mM in PBS buffer/DMSO 1:1 v/v).*

4.2. Kinetic studies

A solution of sodium ascorbate in pH 7.4 PBS buffer/DMSO 1:1 v/v (200 mM) was prepared freshly every working day. A stock solution of each nitroxide (100 mM) was made in DMSO. For kinetic runs, 10 µL of 100 mM stock solution nitroxide was dispensed into an Eppendorf tube by automatic pipette and diluted with 10 µL of PBS buffer, resulting in a 50 mM solution of nitroxide in PBS buffer/DMSO 1:1 v/v. 480 µL of PBS buffer/DMSO 1:1 v/v was added, and the mixture was vortexed, resulting in a 2 mM solution of nitroxide in PBS buffer/DMSO 1:1 v/v. For each kinetic run, 98 µL of 2 mM nitroxide solution was transferred by automatic pipette to an Eppendorf tube. 2 µL of 200 mM ascorbate solution in PBS buffer/DMSO 1:1 v/v was added, and the

timer was started as the tube was vortexed for 6 seconds. The resultant mixture was drawn into a micropipette, stoppered with clay, and placed into the EPR sample cavity in a Bruker microESR Benchtop EPR Spectrometer (X-band). The peak height of the low-field line of the nitroxide triplet was measured as a function of time. Each kinetic run was repeated three times on the same day using the same ascorbate solution. EPR parameters: Microwave power 10 mW, modulation coil amplitude 100%, field range 3400–3588 G, number of points 1600, sweep time 9.3 s. Decay curves of each parallel and averaged plots are presented in Figure S11.

To obtain second-order rate constants, the following integrated rate law equation was used:

$$ln\frac{[C_{A0}][C_N]}{[C_{N0}][C_A]} = k(C_{A0} - C_{N0})t$$

where [C_{A0}], [C_{N0}] are initial concentrations of ascorbate and nitroxide, respectively, and [C_A], [C_N] are concentrations of ascorbate and nitroxide, respectively, at the time *t*. Concentrations of nitroxides at given points in time were calculated from the initial concentration and relative change in intensity of the low-field peak of the nitroxide triplet. The concentration of ascorbate at given points in time was calculated from the concentration of nitroxide, assuming a 1:1 reaction. Logarithmic factors were calculated for each parallel, averaged, and subjected to statistical analysis to obtain standard deviations in Microsoft Excel.

The slope of the initial linear portion of the plots were used to calculate the second-order rate constants (Figure S12). As discussed in the main text of the paper, we observe significant deviation from ideal second-order reaction kinetics (as indicated by deviation from linearity) for rapidly reducing nitroxides; therefore, rate constants were not calculated for nitroxides **12** and **8**. For the majority of spirocyclic nitroxides, the approximated second-order behaviour was observed only in the initial part of the measurement. For tetraethyl nitroxide **13** and dimethylated spirocyclic nitroxide *cis,cis*-**9**, however, linearity was clearly observable for longer time intervals.

The calculated rate constants were compared with literature values where available and found to be in good agreement (Table S1). Nevertheless, considering the differences in solvent and the kinetic model, data should only be compared quantitatively within this article. The error is given as 2 × standard deviation.

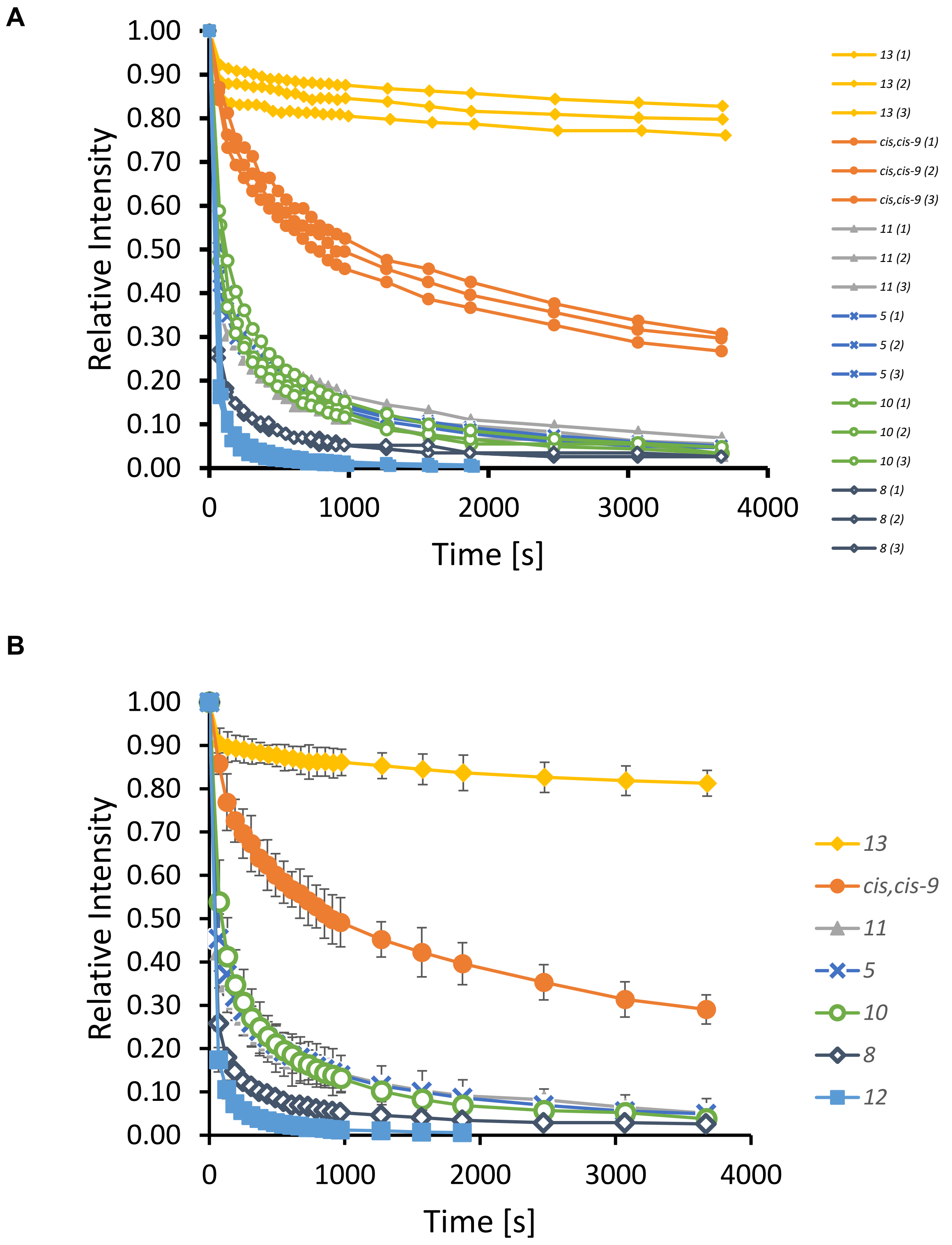

Figure S11. A. *Decay curves of examined nitroxide reductions under second-order conditions (1.4 equiv. of sodium ascorbate) in PBS buffer/DMSO 1:1 v/v at 293 K.* ***B.*** *Average reduction profiles of examined nitroxides. The error bars represent 2 × standard deviation.*

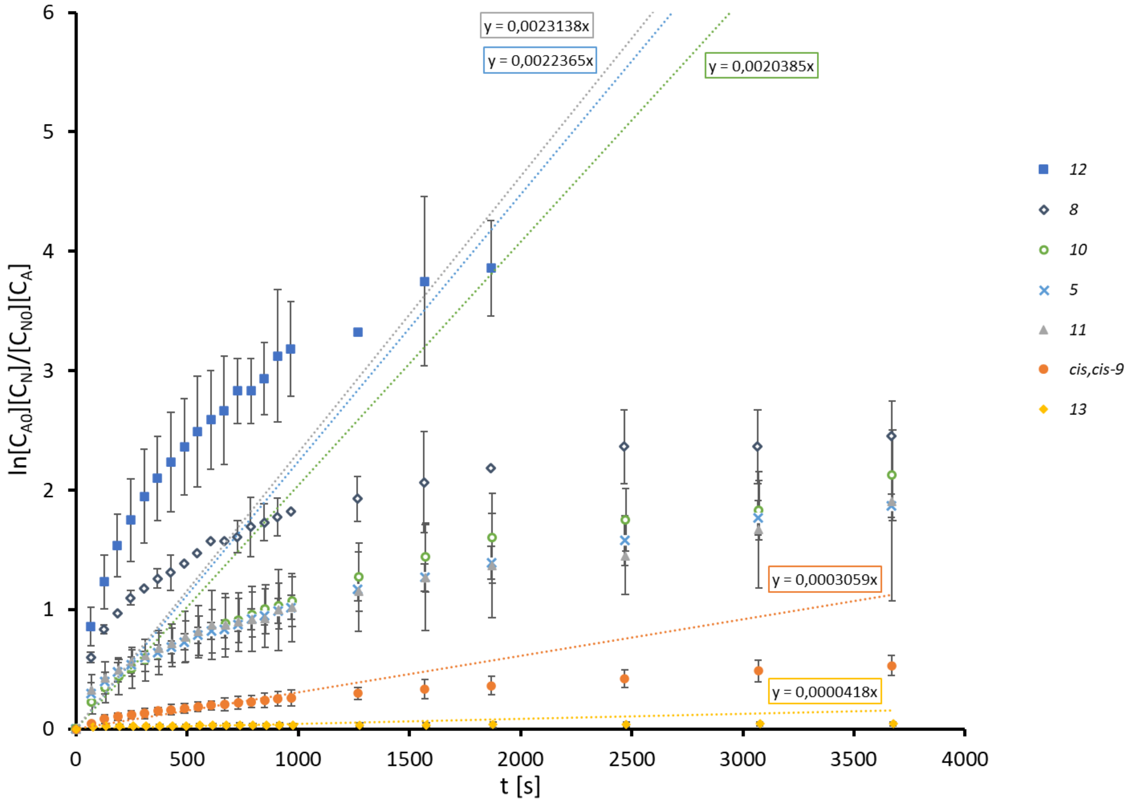

Figure S12. *Semi-logarithmic plots for second-order rate constant determination. The error bars represent 2 × standard deviation.*

Table S1. *Calculated second-order rate constants and literature reference values.*

**Nitroxide**	**k_{exp} [M^{-1} s^{-1}]**	**k_{lit1} [M^{-1} s^{-1}] [2]**	**k_{lit2} [M^{-1} s^{-1}] [4]**
cis,cis-**9**	0.392 ± 0.0779	–	–
**13**	0.0536 ± 0.0215	0.0393 ± 0.0075	0.058 ± 0.002
**5**	2.87 ± 0.295	–	–
**10**	2.61 ± 0.691	3.13 ± 0.21	–
**11**	2.97 ± 0.551	2.56 ± 0.055	–

4.3. Relaxation measurements

1 µL of 100 mM nitroxide stock solution in DMSO was placed in an Eppendorf tube. 999 µL of PBS buffer was added, resulting in a 100 µM solution of nitroxide in PBS buffer/DMSO 99:1 v/v. 30 µL of this solution was transferred to an Eppendorf tube and diluted by 30 µL of glycerol, resulting in a 50 µM solution of nitroxide in glycerol/PBS buffer/DMSO 100:99:1 v/v. This sample solution was placed in a quartz EPR tube and cooled in liquid nitrogen prior to analysis by EPR spectroscopy. A Bruker Elexsys E580 with high powered (150 W) Q-band (34 GHz) and an ER 5106QT-2w cylindrical resonator was used. T_m data was collected by monitoring the echo decay using a 16/32 ns detection sequence with initial separation of 380 ns (τ), 8 ns increment and 700 points at the top of the spectral absorption profile. The shot repetition time was 10 ms, the whole echo was integrated over, and a 2-step phase cycle was used. Most data were collected with 10 shots per point and 1 scan, but some of the higher temperature measurements necessarily required 4 averages. The temperature was incremented from 50 K upwards with a few minutes at each new temperature to allow stabilisation of temperature, but no retuning. The measurements were only taken once (no repeats) and not to equal signal-to-noise (SNR, due to relaxation and sample-variable drift in the tuning with temperature). There is also a deadtime associated with τ, so the phase memory times presented in this paper are only indicative, not fully accurate. Indicative curves are shown in Figure 3 of the main paper, where the time axes is in 2 τ. The data are all available with DOI 10.18710/UQMMZE.

Relaxation times were extracted using the curve fitting tool in Matlab. For simplicity of analysis, all data were fitted with a stretched exponential function $y = a \cdot e^{-(\frac{x}{b})^c + d}$, though this was not always necessary (indicated for cases where the stretch parameter is almost unity), or for the case of the ethyl-substituted nitroxides at lower temperature where the fit was not good for the fast-relaxing part of the curve. However, the extracted T_m give a good indication of the overall trend in phase memory times, dependent on molecule and temperature. The times and stretch parameters are given in Figure S13A and B, respectively.

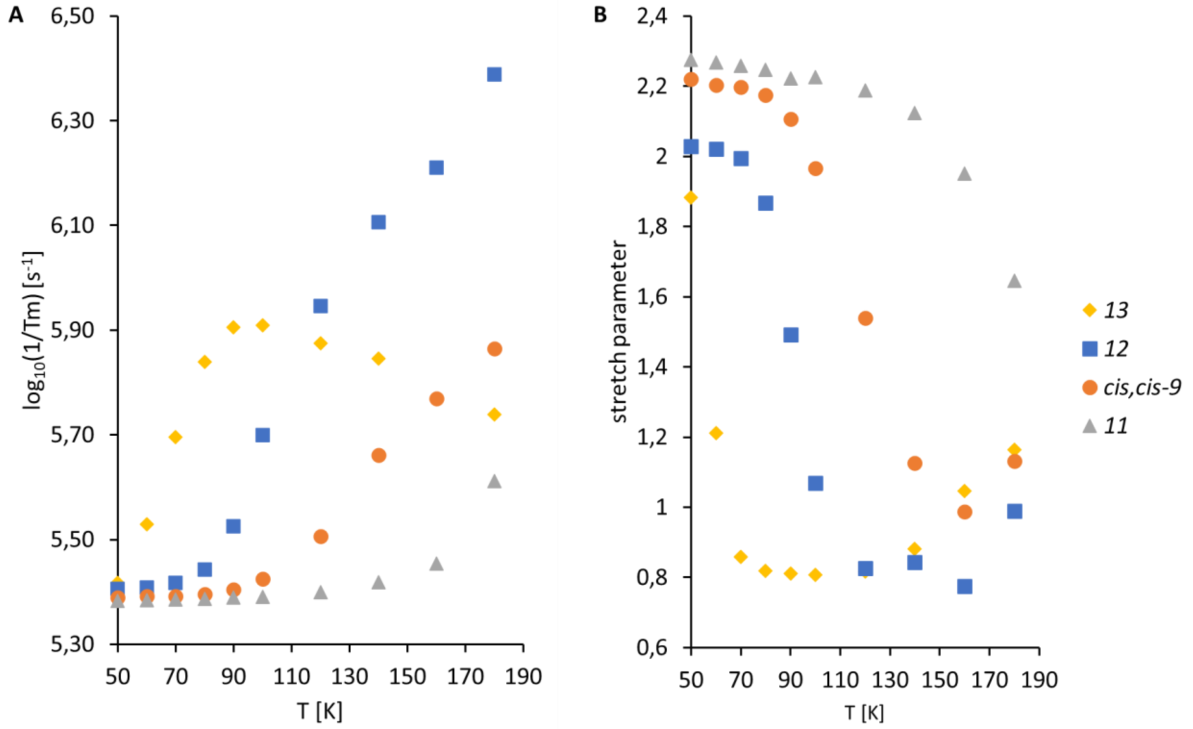

Figure S13. A. *Plots of the phase memory time T_m, measured through spin echo decay, for cis,cis-**9**, **11**, **12** and **13**. **B.** Stretch parameter for fitting the phase memory time plot.*

5. Computations

All calculations were performed on complete molecular systems without any truncations using Gaussian16 (Revision B.01).[5] The systems were fully relaxed, and no symmetry constraints were imposed. For the geometry optimizations, the DFT hybrid functional PBE0,[6,7] which contains 25 percent Hartree-Fock exchange, was used along with the D3BJ[8] dispersion correction (additional calculations with B3LYP[9]-D3 provide the same trends, see Figure S14). The basis set used for geometry optimizations and frequency calculations was def2-TZVPP.[10-13] Solvation effects were included using the polarizable continuum model (IEFPCM)[14] with the parameters of water (ε = 80). Reported Gibbs free energies include thermal corrections computed at 298 K.

The computed Gibbs free energies and Boltzmann distributions of *open, semi-open* and *closed* conformers of alcohol nitroxides are shown in Figure S15. The corresponding data for ketone nitroxides is shown in Figure S16. Both chair and twist-boat-type conformations were evaluated.

The computed Gibbs free energies of nitroxide anions, taking into account both equatorial and axial positions of O^-, are shown in Figure S17. The adiabatic electron affinities were calculated

by taking the energy difference between the energetically lowest-lying conformations of anion and radical nitroxide, respectively.

11

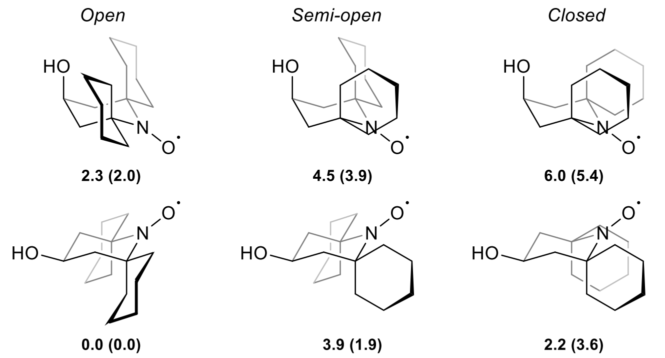

cis,cis-**9**

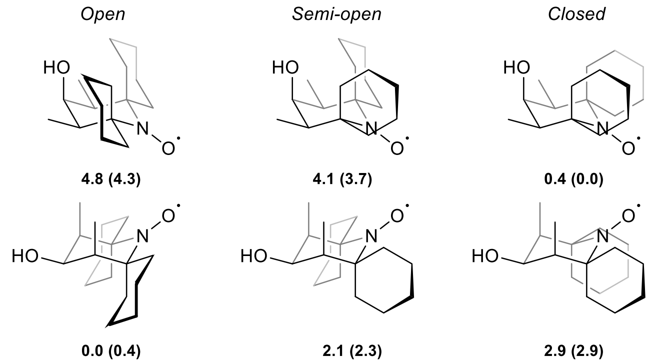

trans,trans-**9**

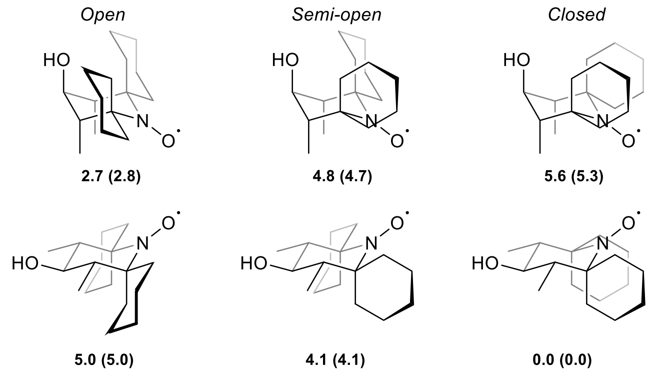

cis,trans-**9**

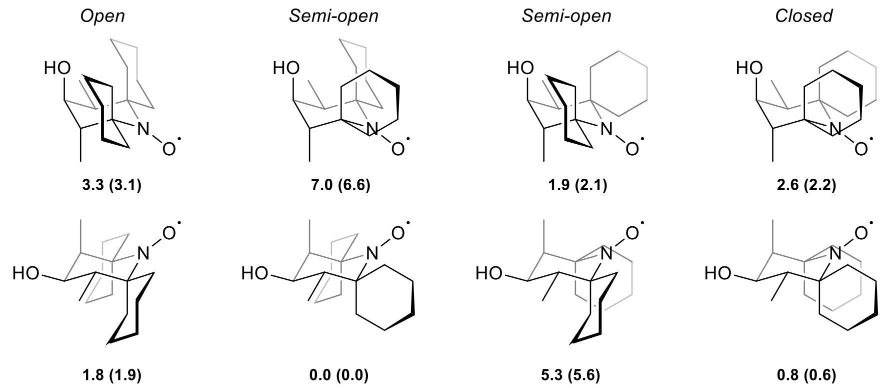

(Continued on next page.)

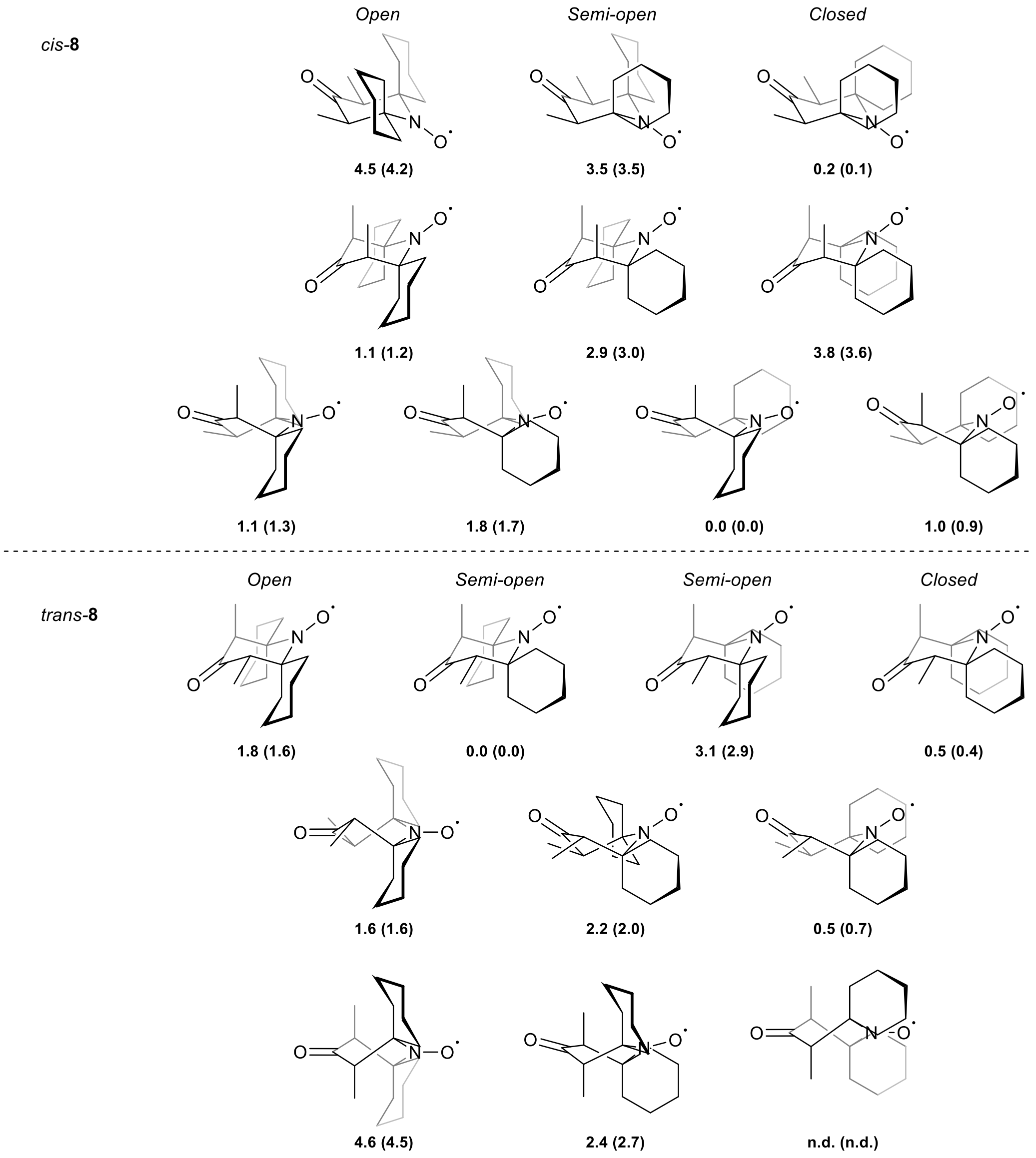

Figure S14. *Calculated Gibbs free energies (in kcal/mol, relative to the most stable conformation) of open, semi-open and closed conformations of nitroxides at the PBE0-D3BJ and B3LYP-D3 (in parenthesis) levels of theory. (n.d. = conformation could not be optimized)*

11

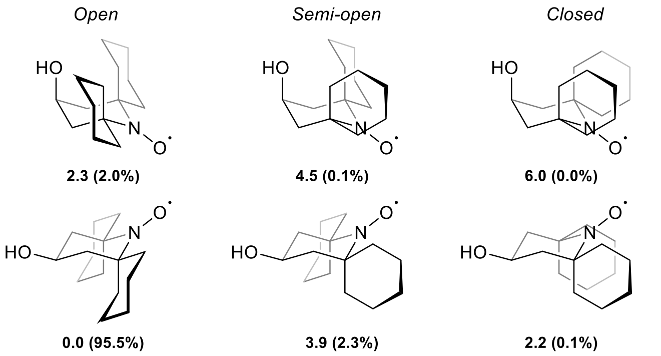

---

cis,cis-**9**

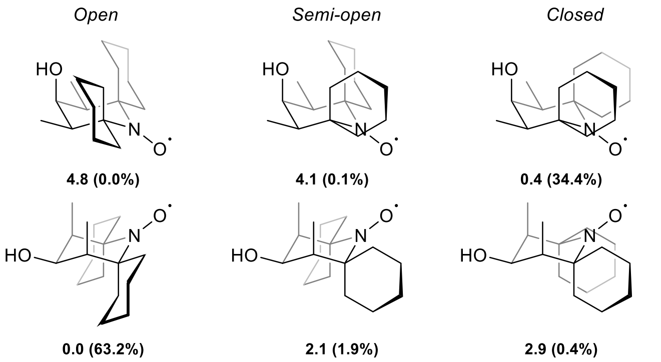

---

trans,trans-**9**

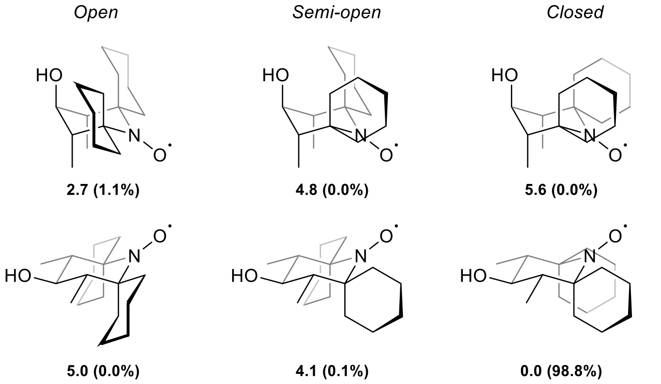

---

cis,trans-**9**

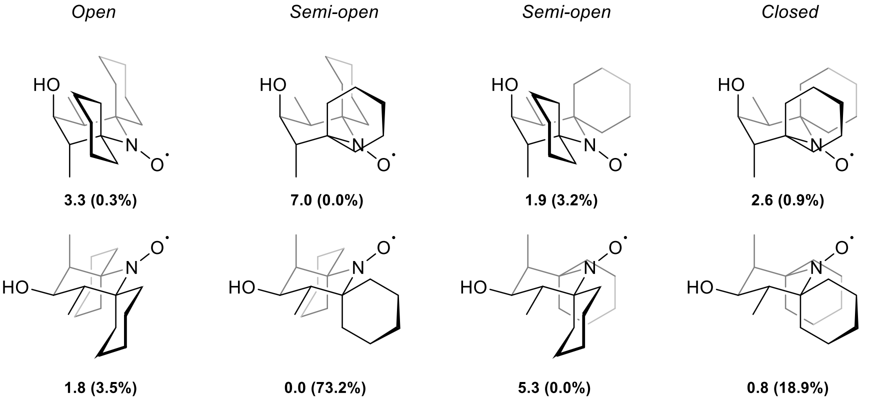

Figure S15. *Calculated Gibbs free energies (in kcal/mol, relative to the most stable conformation) and Boltzmann distribution (parentheses, in percentage) of all open, semi-open and closed conformations of alcohol nitroxides* ***11****, cis,cis-**9**, trans,trans-**9**, and cis,trans-**9** (298 K, PBE0-D3BJ/def2-TZVPP[IEFPCM, water]).*

	Open	*Semi-open*	*Closed*
**10**	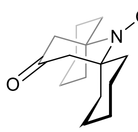<br>**1.7 (5.1%)**	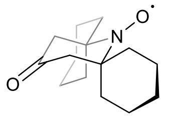<br>**3.7 (0.2%)**	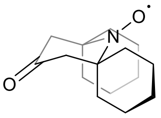<br>**5.1 (0.0%)**
	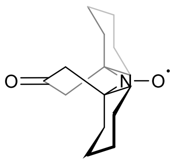<br>**0.0 (84.7%)**	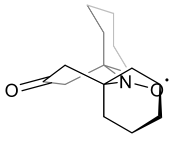<br>**1.3 (9.7%)**	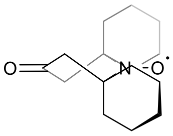<br>**3.3 (0.3%)**

	Open	*Semi-open*	*Semi-open*	*Closed*
**5**	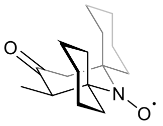<br>**4.2 (0.0%)**	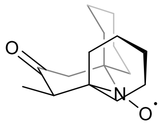<br>**n.d.**	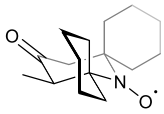<br>**6.5 (0.0%)**	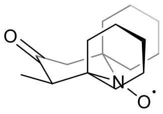<br>**3.9 (0.0%)**
	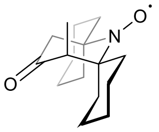<br>**0.3 (20.6%)**	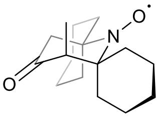<br>**0.8 (8.0%)**	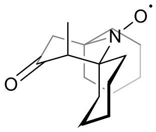<br>**2.8 (0.3%)**	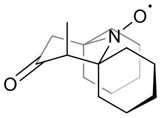<br>**3.1 (0.2%)**
	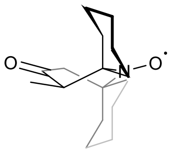<br>**3.1 (0.2%)**	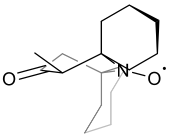<br>**0.2 (21.9%)**	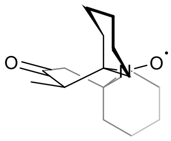<br>**4.1 (0.0%)**	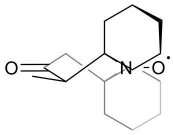<br>**2.8 (0.3%)**
	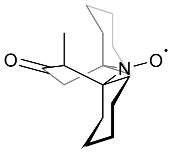<br>**0.0 (33.1%)**	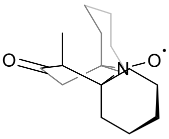<br>**0.5 (14.8%)**	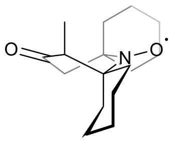<br>**2.7 (0.3%)**	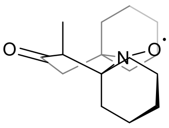<br>**2.9 (0.2%)**

(Continued on the next page.)

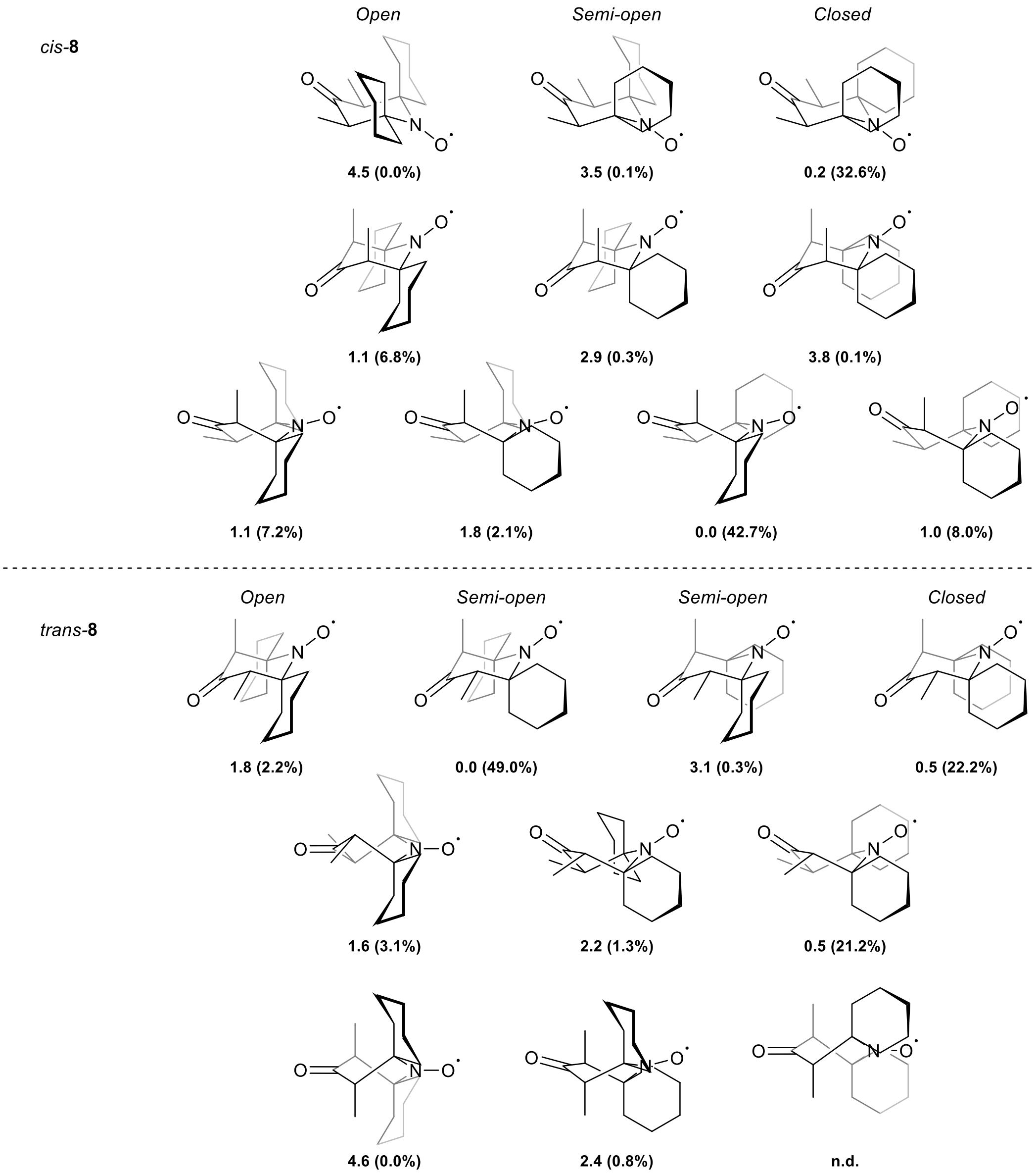

*Figure **S16**. Calculated Gibbs free energies (in kcal/mol, relative to the most stable conformation) and Boltzmann distribution (parentheses, in percentage) of all open, semi-open and closed conformations of the chair- or twist-boat-types of ketone nitroxides **10**, **5**, cis-**8**, and trans-**8** (298 K, PBE0-D3BJ/def2-TZVPP[IEFPCM, water]). (n.d. = conformation could not be optimized)*

11

Open	*Semi-open*	*Closed*
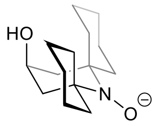	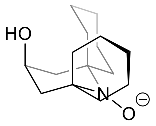	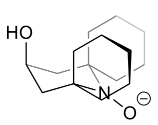
**3.8, 8.4**	**8.0, 7.7**	**13.0, 8.8**
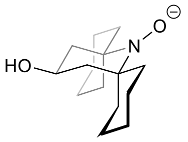	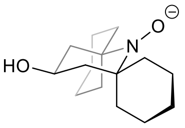	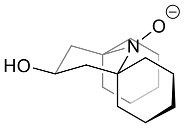
**0.0, 5.5**	**4.5, 4.4**	**9.7, 5.3**

---

cis,cis-**9**

Open	*Semi-open*	*Closed*
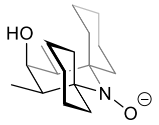	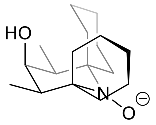	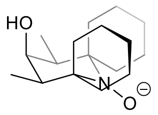
**3.7, 8.9**	**5.9, 4.9**	**8.8, 2.5**
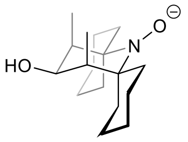	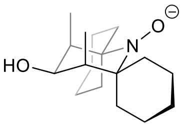	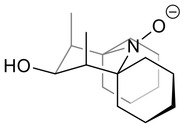
**0.0, 7.3**	**5.7, 6.8**	**10.8, 7.0**

12

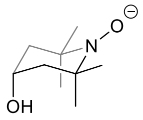

3.6, 7.2

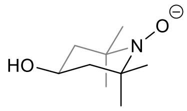

0.0, 4.2

13

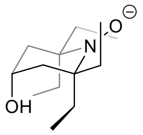

3.6, 6.9

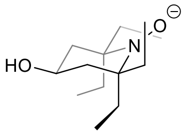

0.0, 1.4

(Continued on the next page.)

10	*Open*	*Semi-open*	*Closed*
	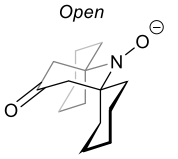	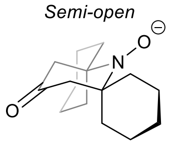	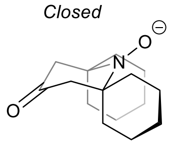
	**0.0, 1.9**	**4.3, 1.5**	**9.7, 1.4**
	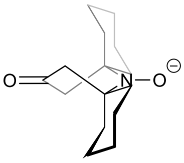	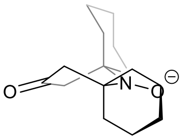	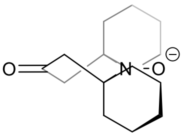
	**0.6, (-8.2)**	**n.d., 5.5**	**n.d., n.d.**

5	*Open*	*Semi-open*	*Semi-open*	*Closed*
	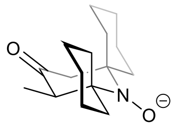	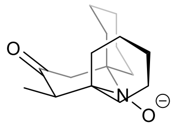	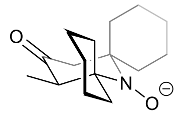	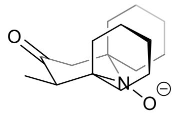
	**2.2, 4.2**	**3.9, 0.0**	**6.6, 2.9**	**9.9, 4.1**
	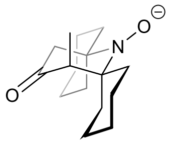	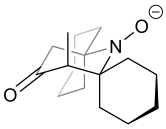	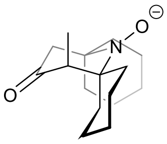	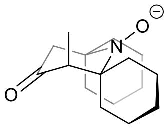
	**0.2, 4.2**	**5.0, 3.7**	**5.1, 3.8**	**9.2, 4.2**
	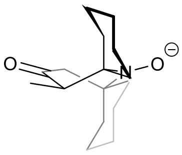	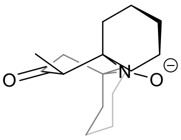	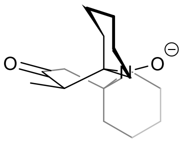	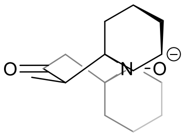
	**2.5, n.d.**	**6.5, 5.5**	**7.0, (-6.4)**	**n.d., n.d.**
	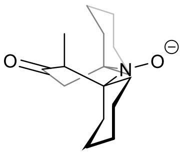	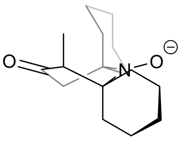	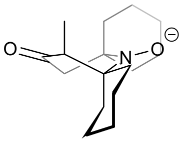	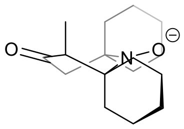
	**2.8, (-8.3)**	**n.d., 8.5**	**n.d., n.d.**	**8.6, (-6.1)**

(Continued on the next page.)

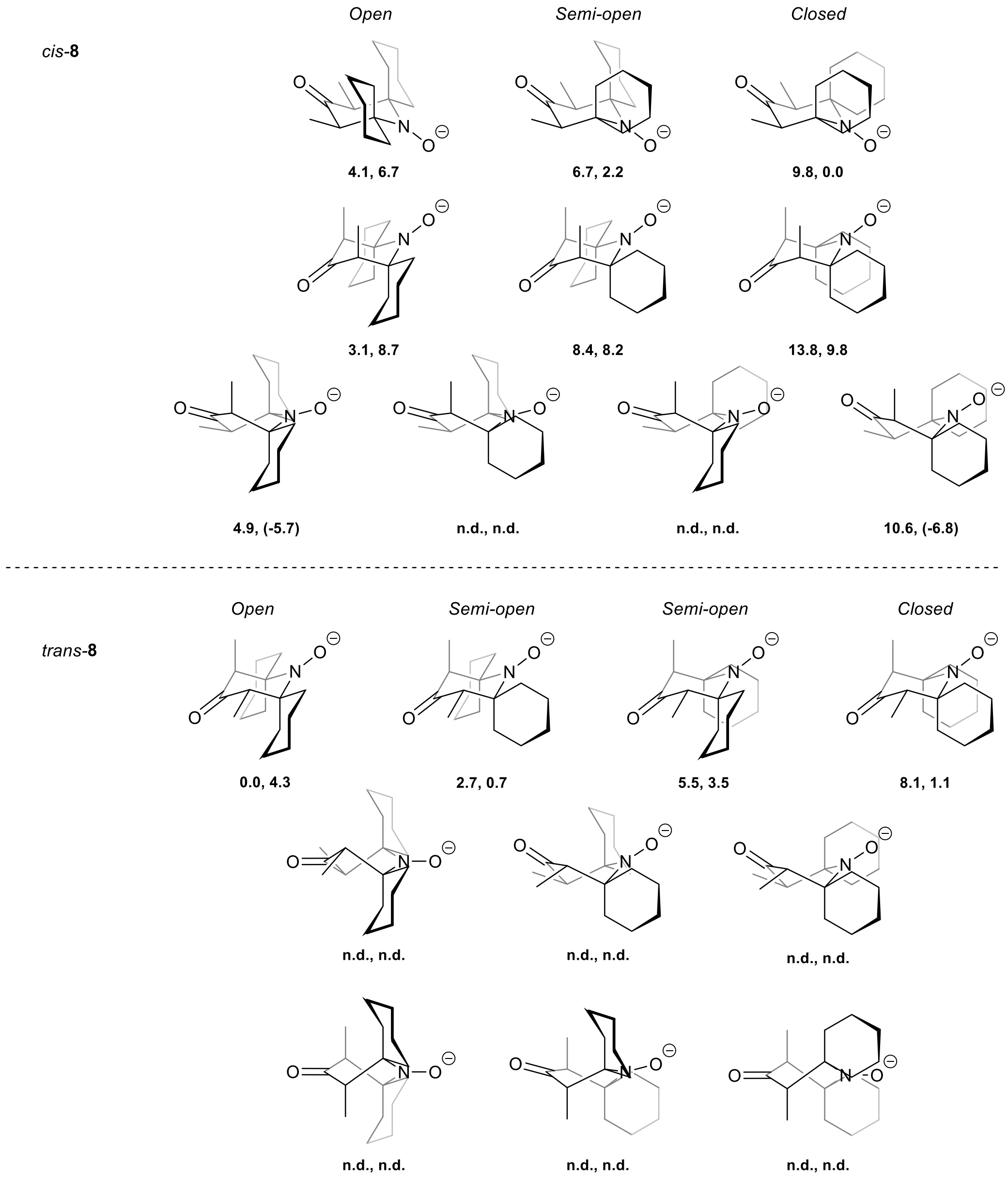

Figure S17. *Calculated Gibbs free energies (in kcal/mol, relative to the most stable conformation) of open, semi-open and closed conformations of nitroxide anions (298 K, PBE0-D3BJ/def2-TZVPP[IEFPCM, water]). The first and second energy values correspond to equatorial and axial position of O^-, respectively. Negative values in parentheses correspond to the formation of a bicyclic structure. (n.d. = conformation could not be optimized)*

Open *Semi-open* *Closed*

11

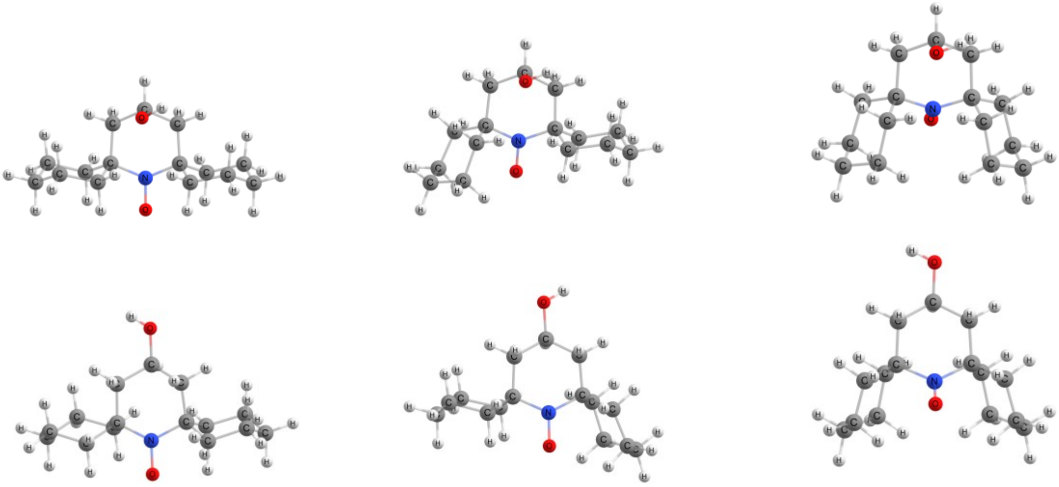

Open *Semi-open* *Closed*

*cis,cis-***9**

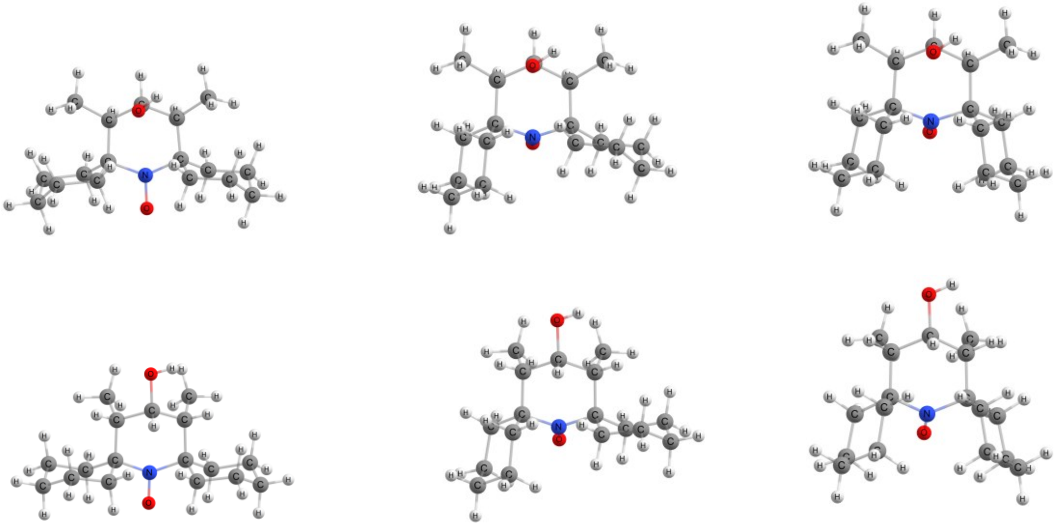

Open *Semi-open* *Closed*

*trans,trans-***9**

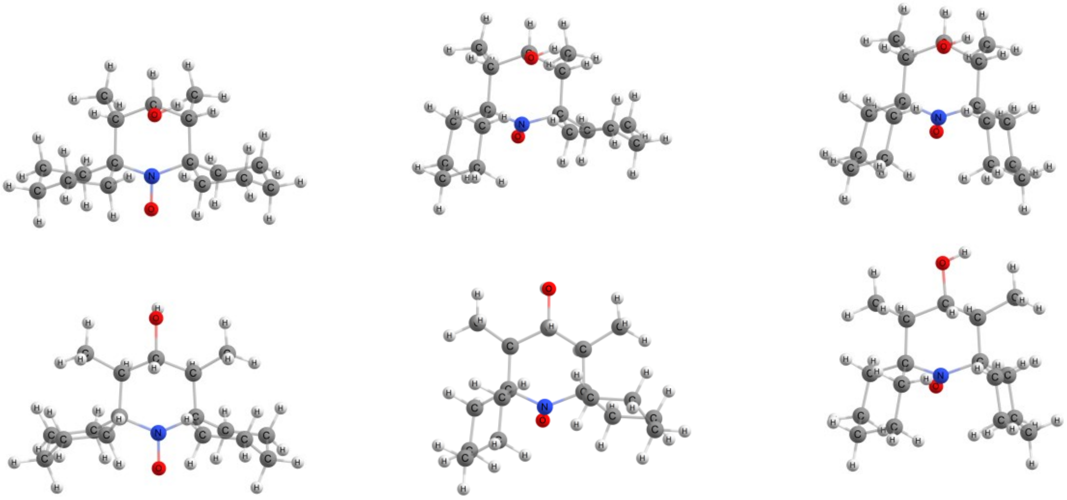

(Continued on the next page.)

Open | *Semi-open* | *Semi-open* | *Closed*

*cis,trans-**9***

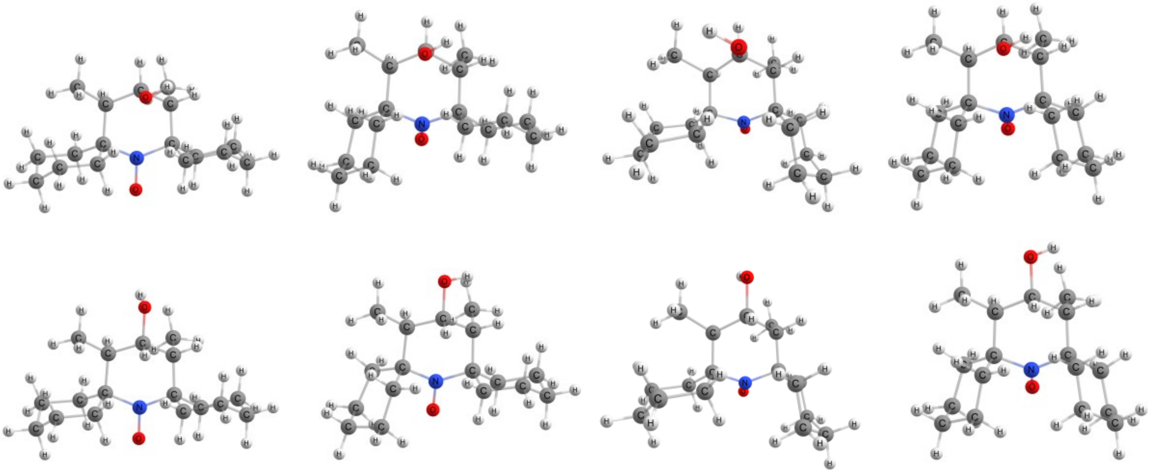

Figure S18. *Optimized geometries of all open, semi-open and closed conformations of alcohol nitroxides **11**, cis,cis-**9**, trans,trans-**9**, and cis,trans-**9** (298 K, PBE0-D3BJ/def2-TZVPP[IEFPCM, water]). The ordering of structures is the same as in Figure S15.*

Table S2. *Calculated energies (Hartrees) of the calculated conformers of nitroxides and their reduced anions (298 K, PBE0-D3BJ/def2-TZVPP[IEFPCM, water]).*

Nitroxide	Conformer description	G [Hartrees]
**11**	open: oh, ax	-791.478631
	semi-open: oh, ax	-791.475176
	closed: oh, ax	-791.472769
	open: oh, eq	-791.482303
	semi-open: oh, eq	-791.478795
	closed: oh, eq	-791.476113
cis,cis-**9**	open: oh, ax	-869.977435
	semi-open: oh, ax	-869.974126
	closed: oh, ax	-869.972744
	open: oh, eq	-869.969861
	semi-open: oh, eq	-869.970877
	closed: oh, eq	-869.97686
trans,trans-**9**	open: ax	-869.975021
	semi-open: ax	-869.97170
	closed: ax	-869.97038
	open: eq	-869.971375
	semi-open: eq	-869.972768
	closed: eq	-869.979304
cis,trans-**9**	open: oh, ax	-869.975261
	semi-open1: oh, ax	-869.977632
	semi-open2: oh, ax	-869.969376
	closed: oh, ax	-869.976443
	open: oh, eq	-869.977723
	semi-open1: oh, eq	-869.980582
	semi-open2: oh, eq	-869.972091
	closed: oh, eq	-869.979304
**12**	oh, eq	-558.315269
	oh, ax	-558.311959
**13**	oh, ax	-715.315036
	oh, eq 1	-715.319026

	oh, eq 2	-715.318866
	oh, eq 3	-715.324984
	oh, eq 4	-715.319505
	oh, eq 5	-715.309944
**10**	open: central ring, chair	-790.299864
	semi-open: central ring, chair	-790.296697
	closed: central ring, chair	-790.294348
	open: central ring, twist-boat	-790.302523
	semi-open: central ring, twist-boat	-790.300478
	closed: central ring, twist-boat	-790.297229
**5**	open, chair : me, eq	-829.546474
	semi-open1, chair : me, eq	-829.542881
	closed, chair : me, eq	-829.547037
	open, chair : me, ax	-829.552758
	semi-open1, chair : me, ax	-829.551861
	semi-open2, chair : me, ax	-829.548678
	closed, chair : me, ax	-829.548186
	open, twist-boat : me, eq	-829.548221
	semi-open1, twist-boat : me, eq	-829.552816
	semi-open2, twist-boat : me, eq	-829.54668
	closed, twist-boat : me, eq	-829.548797
	open, twist-boat : me, ax	-829.553203
	semi-open1, twist-boat : me, ax	-829.552442
	semi-open2, twist-boat : me, ax	-829.548894
	closed, twist-boat : me, ax	-829.548566
cis-**8**	open, both me-eq: central ring, chair	-868.793332
	semi-open, both me-eq: central ring, chair	-868.794944
	closed, both me-eq: central ring, chair	-868.800225
	open, both me-ax: central ring, chair	-868.798747
	semi-open, both me-ax: central ring, chair	-868.795779
	closed, both me-ax: central ring, chair	-868.79437
	open: central ring, twist boat	-868.798803
	semi-open1: central ring, twist boat	-868.80048
	semi-open2: central ring, twist boat	-868.797615
	closed: central ring, twist boat	-868.798904
trans-**8**	open: central ring, chair	-868.798684
	semi-open1: central ring, chair	-868.801626
	semi-open2: central ring, chair	-868.796677
	closed: central ring, chair	-868.80088
	open, both me-eq: central ring, twist-boat	-868.799013
	semi-open, both me-eq: central ring, twist-boat	-868.798173
	closed, both me-eq: central ring, twist-boat	-868.800834
	open, both me-ax: central ring, twist boat	-868.79425
	semi-open, both me-ax: central ring, twist boat	-868.797767
**11** *anion*	open: oh, ax: reduced, eq anion	-791.563232
	open: oh, ax: reduced, ax anion	-791.556022
	semi-open: oh, ax: reduced, eq anion	-791.556573
	semi-open: oh, ax: reduced, ax anion	-791.557006
	closed: oh, ax: reduced, eq anion	-791.548618
	closed: oh, ax: reduced, ax anion	-791.555392
	open: oh, eq: reduced, eq anion	-791.569337
	open: oh, eq: reduced, ax anion	-791.560594
	semi-open: oh, eq: reduced, eq anion	-791.562185

	semi-open: oh, eq: reduced, ax anion	-791.562375
	closed: oh, eq: reduced, eq anion	-791.553801
	closed: oh, eq: reduced, ax anion	-791.560824
cis,cis-**9** *anion*	open, oh ax: reduced, eq anion	-870.058556
	open, oh ax: reduced, ax anion	-870.050292
	semi-open, oh ax: reduced, eq anion	-870.055064
	semi-open, oh ax: reduced, ax anion	-870.05661
	closed, oh ax: reduced, eq anion	-870.050376
	closed, oh ax: reduced, ax anion	-870.060497
	open, oh eq: reduced, eq anion	-870.064448
	open, oh eq: reduced, ax anion	-870.052879
	semi-open, oh eq: reduced, eq anion	-870.055444
	semi-open, oh eq: reduced, ax anion	-870.053594
	closed, oh eq: reduced, eq anion	-870.047306
	closed, oh eq: reduced, ax anion	-870.053251
**12** *anion*	oh, ax: reduced, eq anion	-558.398045
	oh, ax: reduced, ax anion	-558.392362
	oh, eq: reduced, eq anion	-558.403769
	oh, eq: reduced, ax anion	-558.398045
**13** *anion*	oh, ax: reduced, eq anion	-715.389947
	oh, ax: reduced, ax anion	-715.384729
	oh, eq: reduced, eq anion 1	-715.395692
	oh, eq: reduced, eq anion 2	-715.395968
	oh, eq: reduced, eq anion 3	-715.408494
	oh, eq: reduced, eq anion 4	-715.395757
	oh, eq: reduced, eq anion 5	-715.386475
	oh, eq: reduced, ax anion	-715.393529
**10** *anion*	open, chair: reduced, eq anion	-790.393157
	open, chair: reduced, ax anion	-790.390113
	semi-open, chair: reduced, eq anion	-790.386307
	semi-open, chair: reduced, ax anion	-790.390698
	closed, chair: reduced, eq anion	-790.377776
	closed, chair: reduced, ax anion	-790.390916
	open, twist-boat: reduced, eq anion	-790.392228
	open, twist-boat: reduced, ax anion	-790.40616
	semi-open, twist-boat: reduced, ax anion	-790.38445
**5** *anion*	open, me eq, chair: reduced, eq anion	-829.641487
	open, me eq, chair: reduced, ax anion	-829.638338
	semi-open 1, me eq, chair: reduced, eq anion	-829.638757
	semi-open 2, me eq, chair: reduced, eq anion	-829.634406
	semi-open 1, me eq, chair: reduced, ax anion	-829.644952
	semi-open 2, me eq, chair: reduced, ax anion	-829.640262
	closed, me eq, chair: reduced, eq anion	-829.629239
	closed, me eq, chair: reduced, ax anion	-829.643125
	open, me ax, chair: reduced, eq anion	-829.644584
	open, me ax, chair: reduced, ax anion	-829.638266
	semi-open 1, me ax, chair: reduced, eq anion	-829.636918
	semi-open 2, me ax, chair: reduced, eq anion	-829.636814
	semi-open 1, me ax, chair: reduced, ax anion	-829.639087
	semi-open 2, me ax, chair: reduced, ax anion	-829.63896
	closed, me ax, chair: reduced, eq anion	-829.630273
	closed, me ax, chair: reduced, ax anion	-829.638206
	open, me eq, twist-boat: reduced, eq anion	-829.641033

	semi-open 1, me eq, twist-boat: reduced, eq anion	-829.634595
	semi-open 2, me eq, twist-boat: reduced, eq anion	-829.633778
	semi-open 1, me eq, twist-boat: reduced, ax anion	-829.636129
	semi-open 2, me eq, twist-boat: reduced, ax anion	-829.655159
	open, me ax, twist-boat: reduced, eq anion	-829.640536
	open, me ax, twist-boat: reduced, ax anion	-829.658109
	semi-open, me ax, twist-boat: reduced, ax anion	-829.631344
	closed, me ax, twist-boat: reduced, eq anion	-829.631283
	closed, me ax, twist-boat: reduced, ax anion	-829.654701
cis-**8** *anion*	open, chair, me eq: reduced, eq anion	-868.889982
	open, chair, me eq: reduced, ax anion	-868.885867
	semi-open, chair, me eq: reduced, eq anion	-868.885897
	semi-open, chair, me eq: reduced, ax anion	-868.893059
	closed, chair, me eq: reduced, eq anion	-868.881017
	closed, chair, me eq: reduced, ax anion	-868.896563
	open, chair, me ax: reduced, eq anion	-868.891621
	open, chair, me ax: reduced, ax anion	-868.882685
	semi-open, chair, me ax: reduced, eq anion	-868.883167
	semi-open, chair, me ax: reduced, ax anion	-868.88355
	closed, chair, me ax: reduced, eq anion	-868.874577
	closed, chair, me ax: reduced, ax anion	-868.881019
	open, twist-boat: reduced, eq anion	-868.88872
	open, twist-boat: reduced, ax anion	-868.905701
	closed, twist-boat: reduced, eq anion	-868.879633
	closed, twist-boat: reduced, ax anion	-868.907372
trans-**8** *anion*	open, chair: reduced, eq anion	-868.893523
	open, chair: reduced, ax anion	-868.886595
	semi-open 1, chair: reduced, eq anion	-868.889205
	semi-open 2, chair: reduced, eq anion	-868.884807
	semi-open 1, chair: reduced, ax anion	-868.89244
	semi-open 2, chair: reduced, ax anion	-868.887998
	closed, chair: reduced, eq anion	-868.880573
	closed, chair: reduced, ax anion	-868.891697

6. Supplementary References

1 Sakai, K. *et al.* Effective 2,6-substitution of piperidine nitroxyl radical by carbonyl compound. *Tetrahedron* **66**, 2311-2315 (2010). https://doi.org:https://doi.org/10.1016/j.tet.2010.02.004

2 Paletta, J. T., Pink, M., Foley, B., Rajca, S. & Rajca, A. Synthesis and Reduction Kinetics of Sterically Shielded Pyrrolidine Nitroxides. *Org. Lett.* **14**, 5322-5325 (2012). https://doi.org:10.1021/ol302506f

3 Wetter, C. *et al.* Steric and Electronic Effects in Cyclic Alkoxyamines—Synthesis and Applications as Regulators for Controlled/Living Radical Polymerization. *Chem. - Eur. J.* **10**, 1156-1166 (2004). https://doi.org:10.1002/chem.200305427

4 Jagtap, A. P. *et al.* Sterically shielded spin labels for in-cell EPR spectroscopy: Analysis of stability in reducing environment. *Free Radic. Res.* **49**, 78-85 (2015). https://doi.org:10.3109/10715762.2014.979409

5 M. J. Frisch, G. W. Trucks, H. B. Schlegel, G. E. Scuseria, M. A. Robb, J. R. Cheeseman, G. Scalmani, V. Barone, G. A. Petersson, H. Nakatsuji, X. Li, M. Caricato, A. V. Marenich, J. Bloino, B. G. Janesko, R. Gomperts, B. Mennucci, H. P.

Hratchian, J. V. Ortiz, A. F. Izmaylov, J. L. Sonnenberg, Williams, F. Ding, F. Lipparini, F. Egidi, J. Goings, B. Peng, A. Petrone, T. Henderson, D. Ranasinghe, V. G. Zakrzewski, J. Gao, N. Rega, G. Zheng, W. Liang, M. Hada, M. Ehara, K. Toyota, R. Fukuda, J. Hasegawa, M. Ishida, T. Nakajima, Y. Honda, O. Kitao, H. Nakai, T. Vreven, K. Throssell, J. A. Montgomery Jr., J. E. Peralta, F. Ogliaro, M. J. Bearpark, J. J. Heyd, E. N. Brothers, K. N. Kudin, V. N. Staroverov, T. A. Keith, R. Kobayashi, J. Normand, K. Raghavachari, A. P. Rendell, J. C. Burant, S. S. Iyengar, J. Tomasi, M. Cossi, J. M. Millam, M. Klene, C. Adamo, R. Cammi, J. W. Ochterski, R. L. Martin, K. Morokuma, O. Farkas, J. B. Foresman and D. J. Fox, *Gaussian, Inc.*, Wallingford CT, 2016.

6 Perdew, J. P., Burke, K. & Ernzerhof, M. Generalized Gradient Approximation Made Simple. *Phys. Rev. Lett.* **77**, 3865-3868 (1996). https://doi.org:10.1103/physrevlett.77.3865

7 Adamo, C. & Barone, V. Toward reliable density functional methods without adjustable parameters: The PBE0 model. *J. Chem. Phys.* **110**, 6158-6170 (1999). https://doi.org:10.1063/1.478522

8 Grimme, S., Ehrlich, S. & Goerigk, L. Effect of the damping function in dispersion corrected density functional theory. *J. Comput. Chem.* **32**, 1456-1465 (2011). https://doi.org:10.1002/jcc.21759

9 Becke, A. D. Density-functional thermochemistry. III. The role of exact exchange. *J. Chem. Phys.* **98**, 5648-5652 (1993). https://doi.org:10.1063/1.464913

10 Weigend, F. & Ahlrichs, R. Balanced basis sets of split valence, triple zeta valence and quadruple zeta valence quality for H to Rn: Design and assessment of accuracy. *Phys. Chem. Chem. Phys.* **7**, 3297 (2005). https://doi.org:10.1039/b508541a

11 Schäfer, A., Horn, H. & Ahlrichs, R. Fully optimized contracted Gaussian basis sets for atoms Li to Kr. *J. Chem. Phys.* **97**, 2571-2577 (1992). https://doi.org:10.1063/1.463096

12 Schäfer, A., Huber, C. & Ahlrichs, R. Fully optimized contracted Gaussian basis sets of triple zeta valence quality for atoms Li to Kr. *J. Chem. Phys.* **100**, 5829-5835 (1994). https://doi.org:10.1063/1.467146

13 Weigend, F. Accurate Coulomb-fitting basis sets for H to Rn. *Phys. Chem. Chem. Phys.* **8**, 1057 (2006). https://doi.org:10.1039/b515623h

14 Scalmani, G. & Frisch, M. J. Continuous surface charge polarizable continuum models of solvation. I. General formalism. *J. Chem. Phys.* **132**, 114110 (2010). https://doi.org:10.1063/1.3359469
