# Supplementary material for: Conformational tuning improves the stability of spirocyclic nitroxides with long paramagnetic relaxation times
Source: Commun Chem. 2023 Jun 5;6:111. doi: 10.1038/s42004-023-00912-7 (PMC10241799; doi:10.1038/s42004-023-00912-7)
Supplement: Supplementary file 3 — Description of Additional Supplementary Files [file 42004_2023_912_MOESM3_ESM.pdf]

Description of Additional Supplementary Files

File name: Supplementary Data 1
Description: NMR spectra

File name: Supplementary Data 2
Description: CIF file of nitroxide 5 (CCDC no. 2214626)

File name: Supplementary Data 3
Description: SCXRD report of nitroxide 5 (CCDC no. 2214626)

File name: Supplementary Data 4
Description: CIF file of nitroxide trans-8 (CCDC no. 2214625)

File name: Supplementary Data 5
Description: SCXRD report of nitroxide trans-8 (CCDC no. 2214625)

File name: Supplementary Data 6
Description: CIF file of nitroxide cis,cis-9 (CCDC no. 2214627)

File name: Supplementary Data 7
Description: SCXRD report of nitroxide cis,cis-9 (CCDC no. 2214627)

File name: Supplementary Data 8
Description: XYZ file containing coordinates and energies of all calculated conformers
