# Supplementary material for: Conformational tuning improves the stability of spirocyclic nitroxides with long paramagnetic relaxation times
Source: Commun Chem. 2023 Jun 5;6:111. doi: 10.1038/s42004-023-00912-7 (PMC10241799; doi:10.1038/s42004-023-00912-7)
Supplement: Supplementary file 4 — Supplementary Data 1 [file 42004_2023_912_MOESM4_ESM.pdf]

Supplementary Data 1
NMR spectra

Conformational tuning improves the stability of spirocyclic nitroxides with long paramagnetic relaxation times

Mateusz P. Sowiński,[a] Sahil Gahlawat,[a,b] Anna-Luisa Warnke,[a†] Bjarte A. Lund,[a†] Kathrin H. Hopmann,[a] Janet E. Lovett,[c] Marius M. Haugland [a]*

[†] These authors contributed equally
** e-mail: marius.m.haugland@uit.no*

[a] Department of Chemistry, UiT The Arctic University of Norway, 9037 Tromsø, Norway

[b] Hylleraas Center for Quantum Molecular Sciences, UiT The Arctic University of Norway, 9037 Tromsø, Norway

[c] SUPA, School of Physics and Astronomy and BSRC, University of St Andrews, North Haugh, St Andrews, KY16 9SS, UK

3

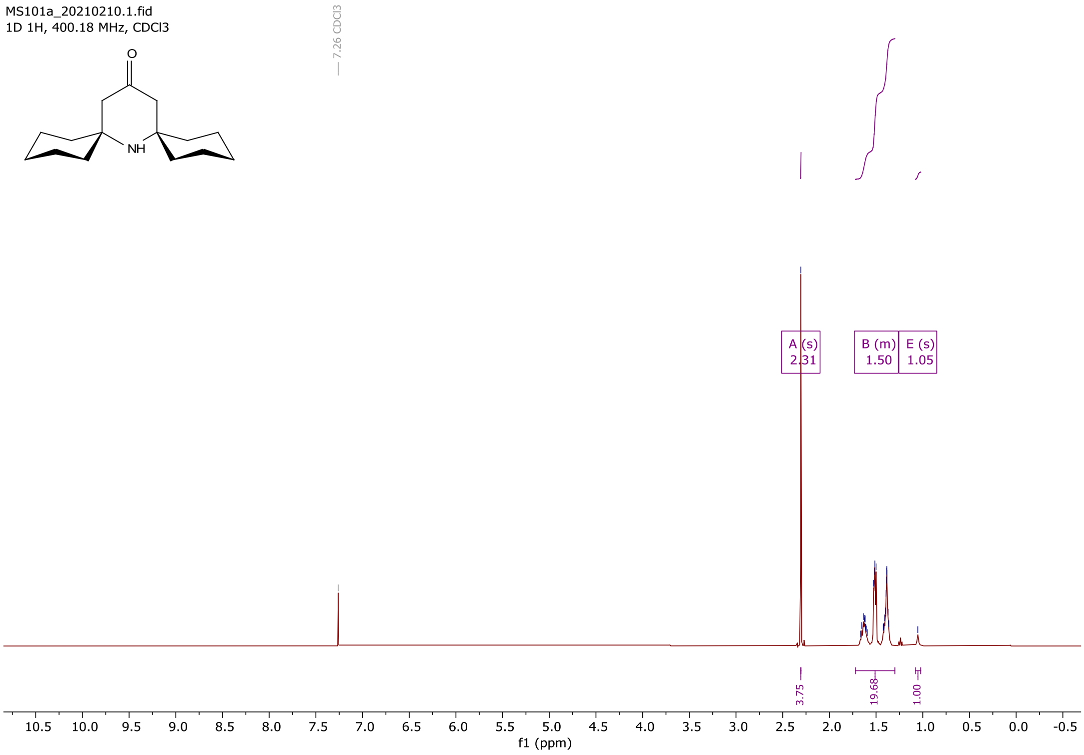

^{1}H NMR (400 MHz, $CDCl_3$) δ 2.31 (4H, s), 1.72–1.30 (20H, m), 1.05 (1H, s).

*Figure S19. ^{1}H NMR spectrum of **3**.*

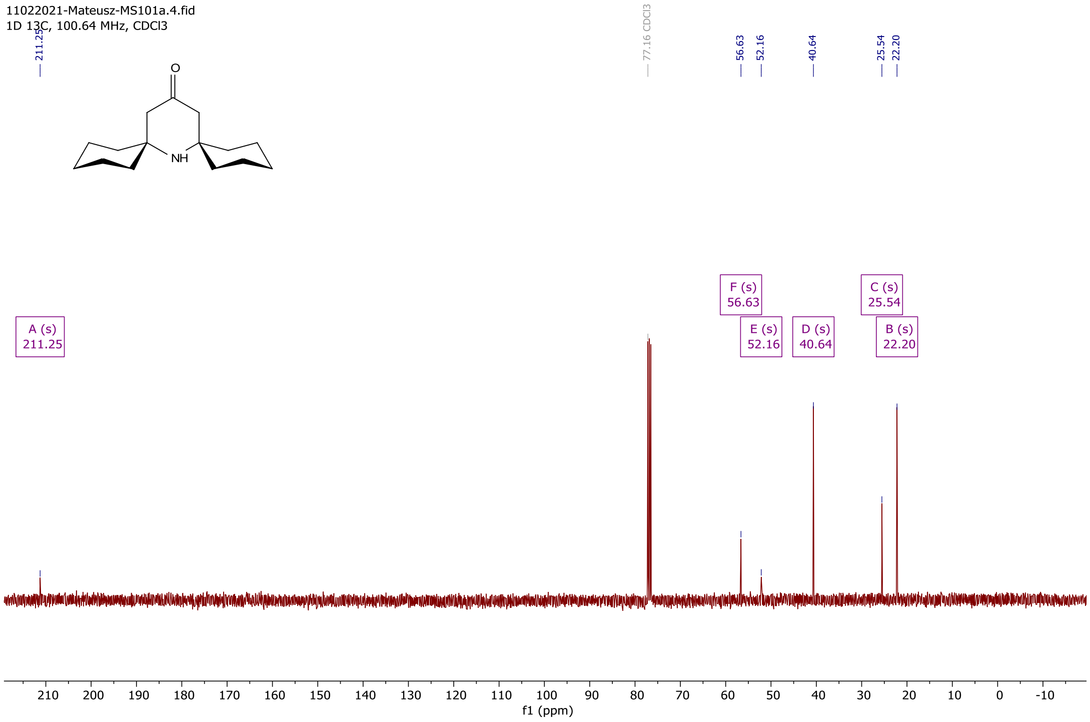

^{13}C NMR (101 MHz, $CDCl_3$) δ 211.25, 56.63, 52.16, 40.64, 25.54, 22.20.

*Figure S20. ^{13}C NMR spectrum of **3**.*

4

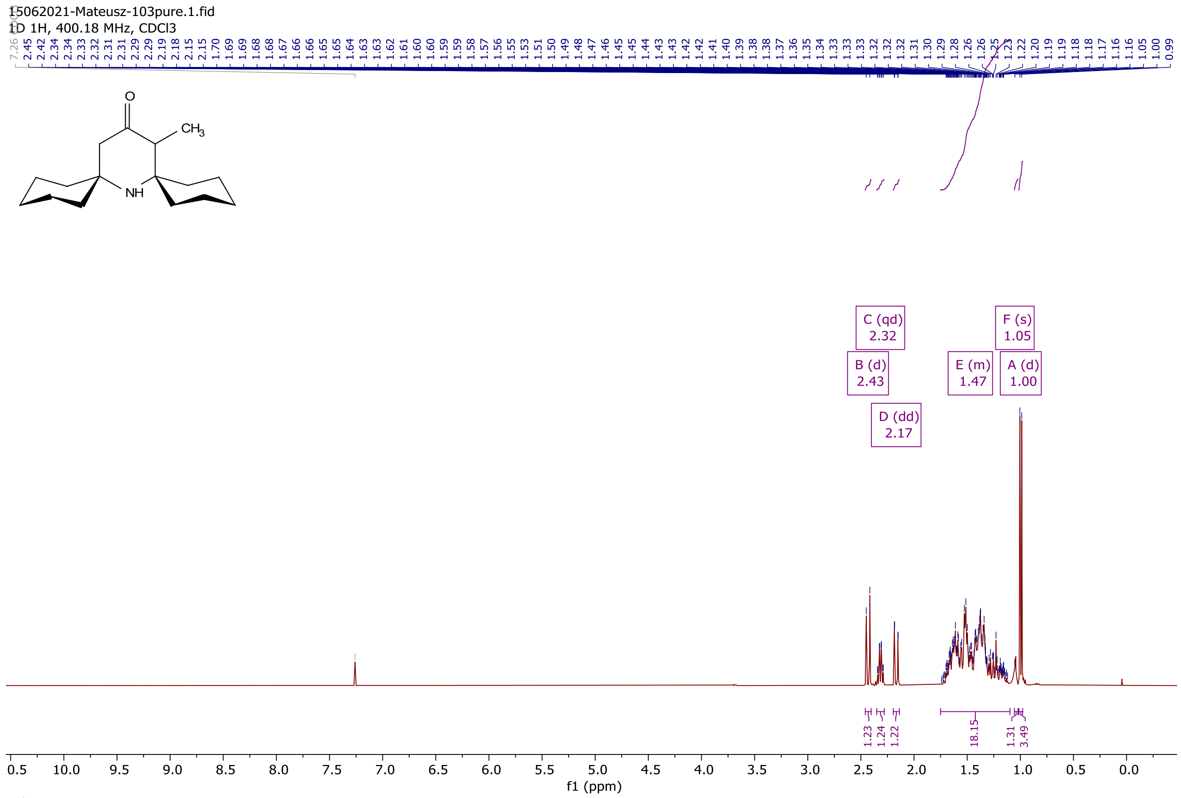

^{1}H NMR (400 MHz, $CDCl_3$) δ 2.43 (1H, d, J = 13.3 Hz), 2.32 (1H, qd, J = 6.9, 1.2 Hz), 2.17 (1H, dd, J = 13.3, 1.2 Hz), 1.75–1.10 (18H, m), 1.05 (1H, s), 1.00 (3H, d, J = 6.9 Hz).

*Figure S21. ^{1}H NMR spectrum of **4**.*

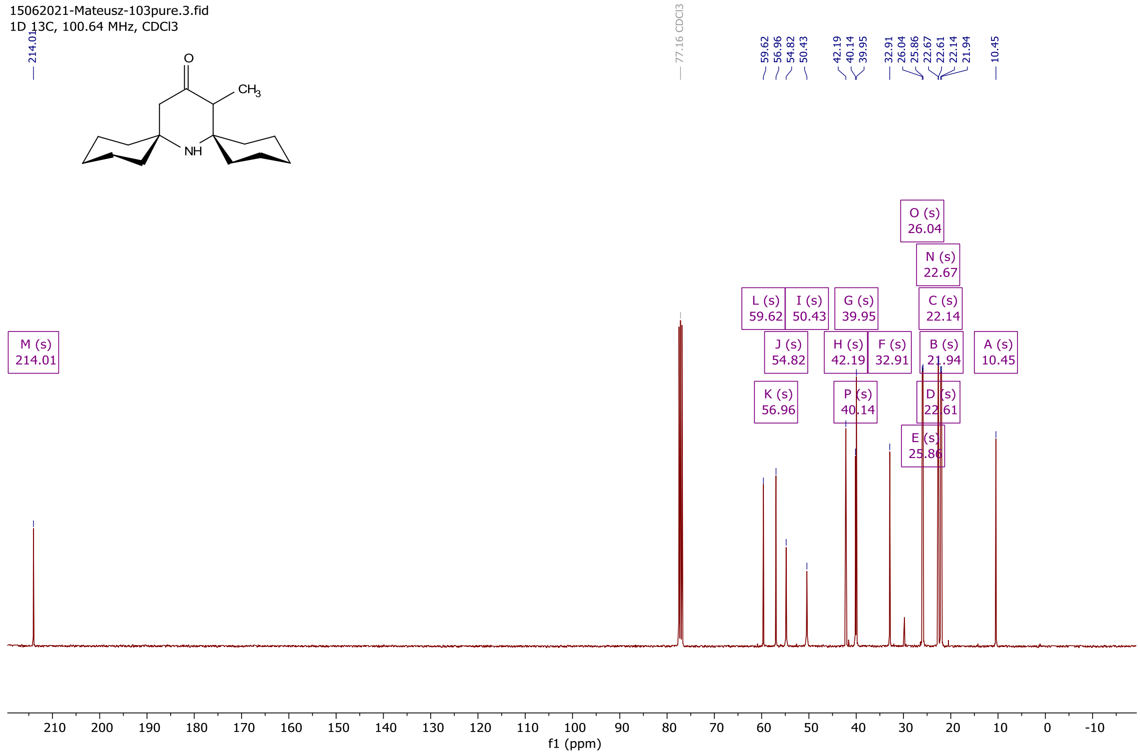

^{13}C NMR (101 MHz, $CDCl_3$) δ 214.01, 59.62, 56.96, 54.82, 50.43, 42.19, 40.14, 39.95, 32.91, 26.04, 25.86, 22.67, 22.61, 22.14, 21.94, 10.45.

*Figure S22. ^{13}C NMR spectrum of **4**.*

5’

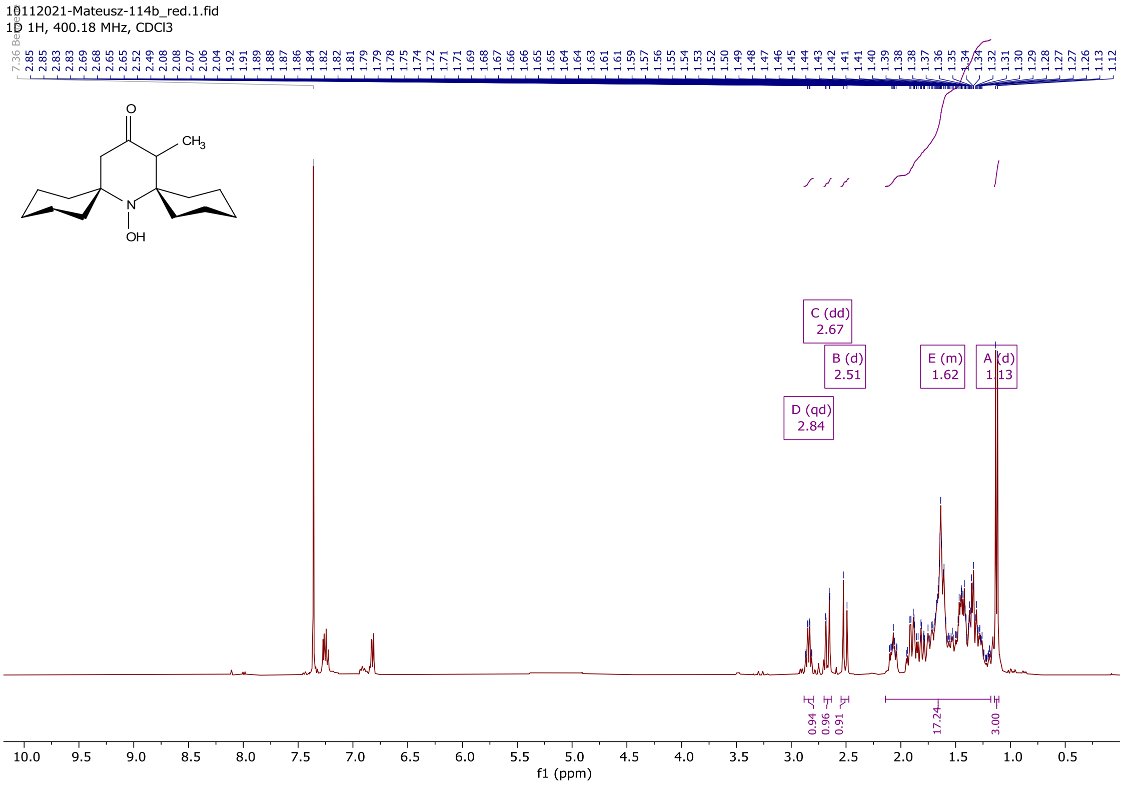

^{1}**H NMR** (400 MHz, $CDCl_3$) δ 2.84 (1H, qd, *J* = 7.0, 1.7 Hz), 2.67 (1H, dd, *J* = 13.4, 1.8 Hz), 2.51 (1H, d, *J* = 13.4 Hz), 2.14–1.18 (17H, m), 1.13 (3H, d, *J* = 7.0 Hz).

*Figure S23. ^{1}H NMR spectrum of **5’**.*

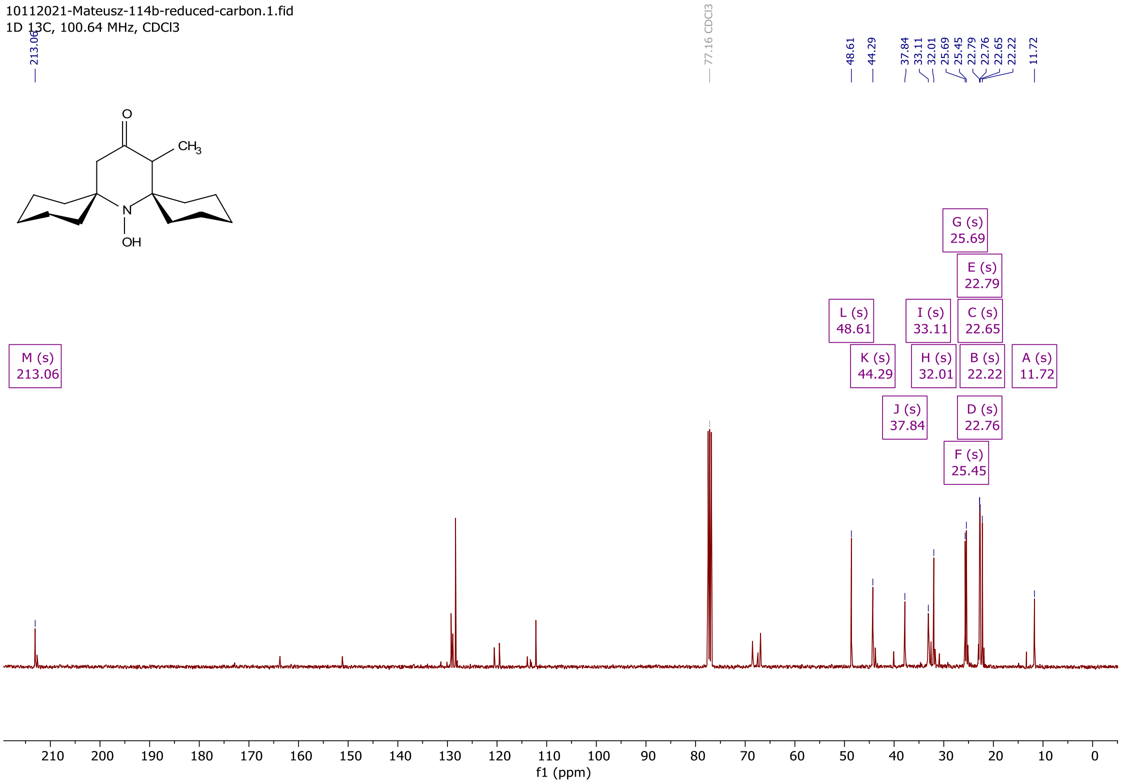

^{13}**C NMR** (101 MHz, $CDCl_3$) δ 213.06, 48.61, 44.29, 37.84, 33.11, 32.01, 25.69, 25.45, 22.79, 22.76, 22.65, 22.22, 11.72.

*Figure S24. ^{13}C NMR spectrum of **5’**.*

6

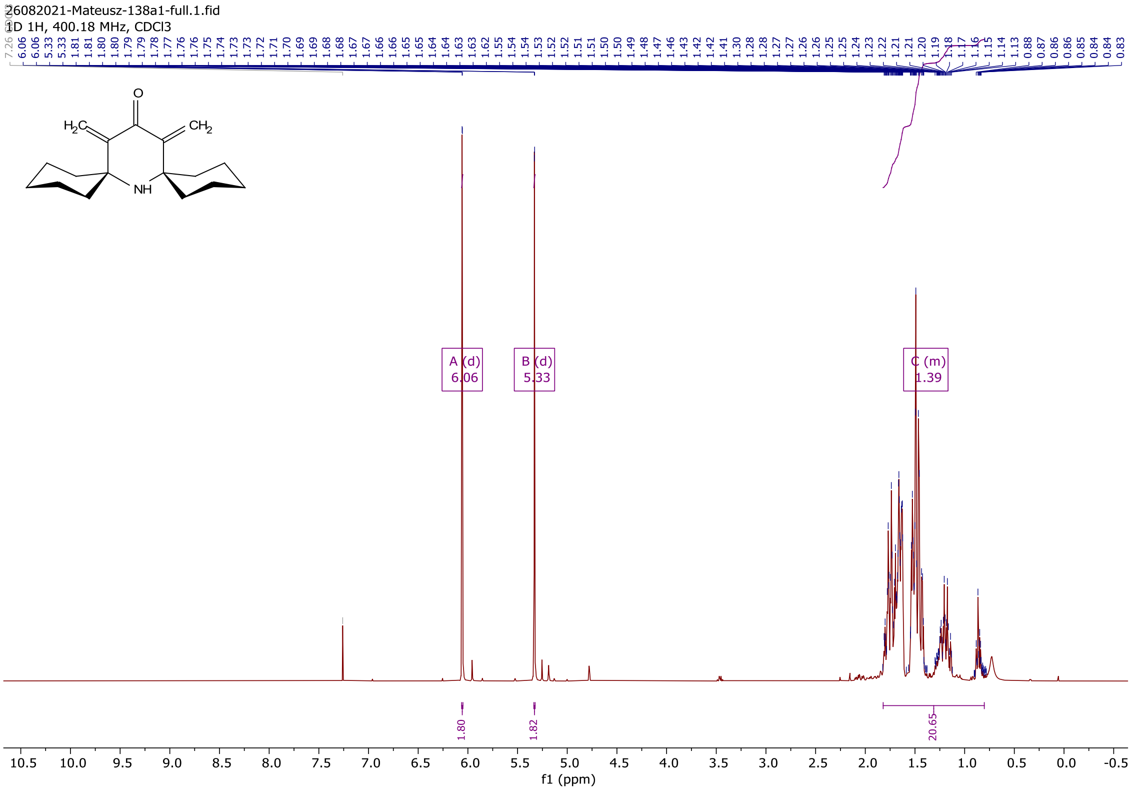

^{1}**H NMR** (400 MHz, $CDCl_3$) δ 6.06 (2H, d, *J* = 1.1 Hz), 5.33 (2H, d, *J* = 1.2 Hz), 1.82–0.80 (21H, m).

*Figure S25. ^{1}H NMR spectrum of **6**.*

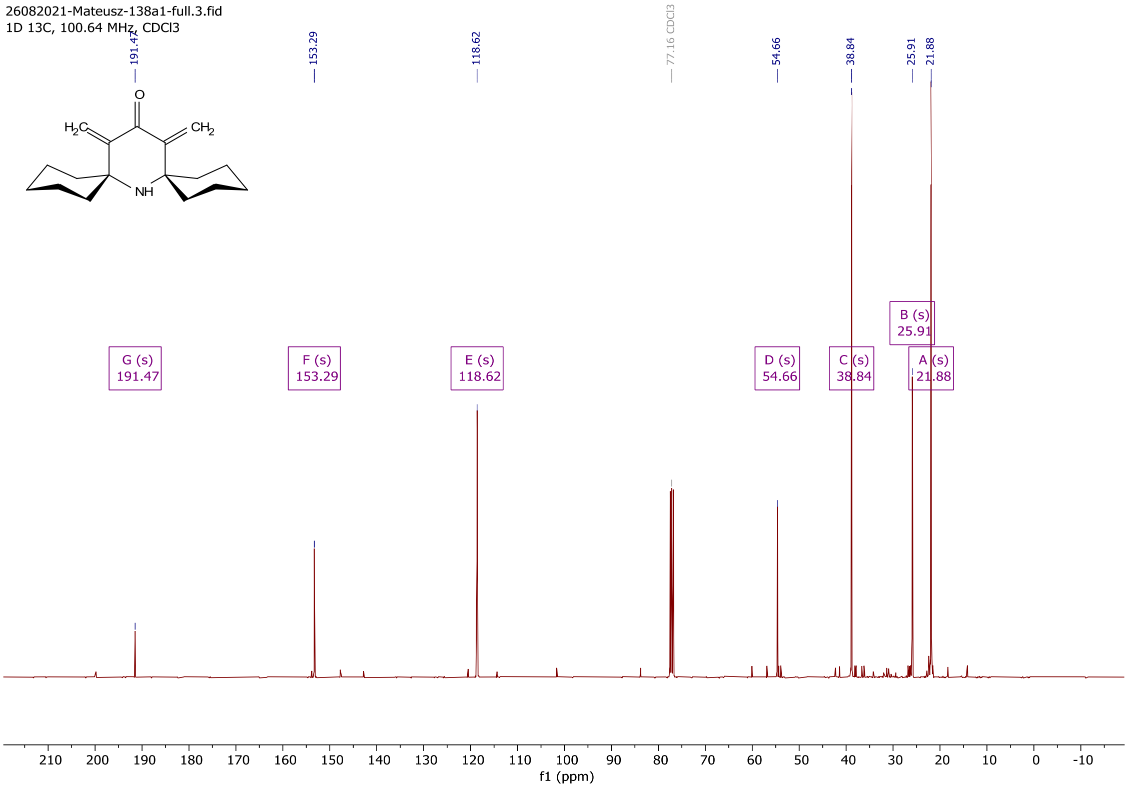

^{13}**C NMR** (101 MHz, $CDCl_3$) δ 191.47, 153.29, 118.62, 54.66, 38.84, 25.91, 21.88.

*Figure S26. ^{13}C NMR spectrum of **6**.*

7

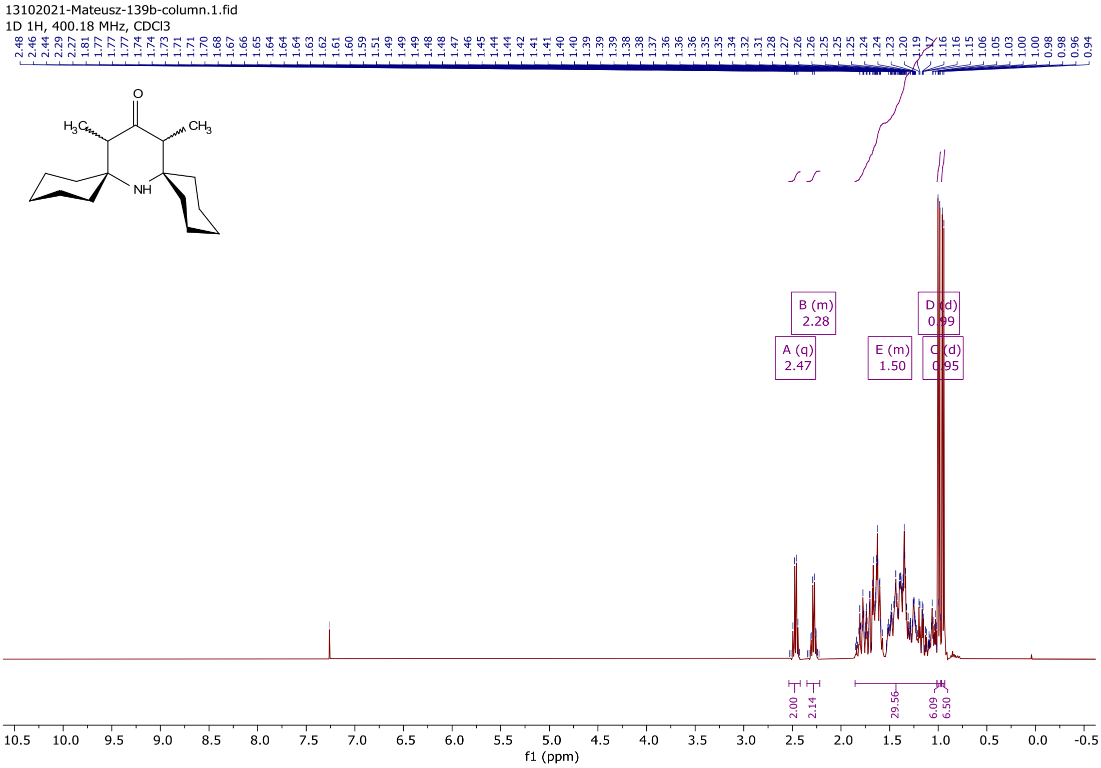

^{1}H NMR (400 MHz, $CDCl_3$) δ 2.47 (2H, q, J = 6.9 Hz), 2.35–2.22 (2H, m), 1.85–1.01 (30H, m), 0.99 (6H, d, J = 7.0 Hz), 0.95 (6H, d, J = 6.8 Hz).

*Figure S27. ^{1}H NMR spectrum of **7**.*

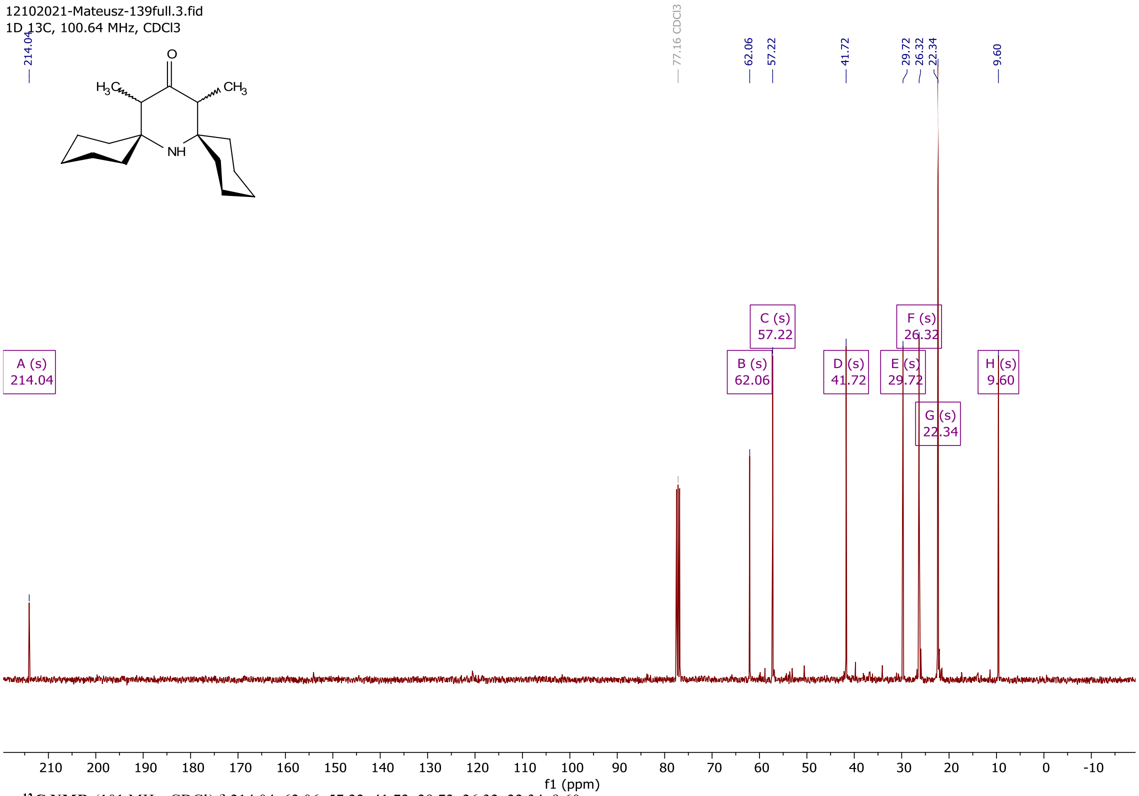

^{13}C NMR (101 MHz, $CDCl_3$) δ 214.04, 62.06, 57.22, 41.72, 29.72, 26.32, 22.34, 9.60.

*Figure S28. ^{13}C NMR spectrum of **7**.*

8'

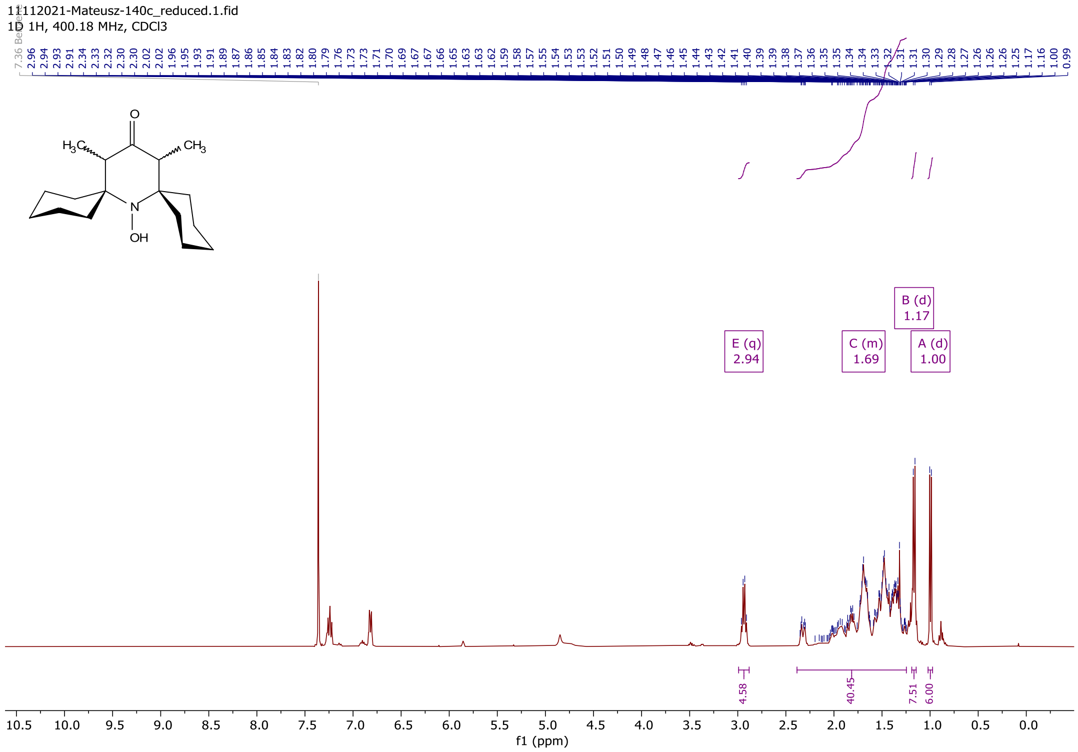

^{1}H NMR (400 MHz, $CDCl_3$) δ 2.94 (5H, q, J = 6.9 Hz), 2.38–1.25 (40H, m), 1.17 (8H, d, J = 7.1 Hz), 1.00 (6H, d, J = 6.8 Hz).

*Figure S29. ^{1}H NMR spectrum of **8'**.*

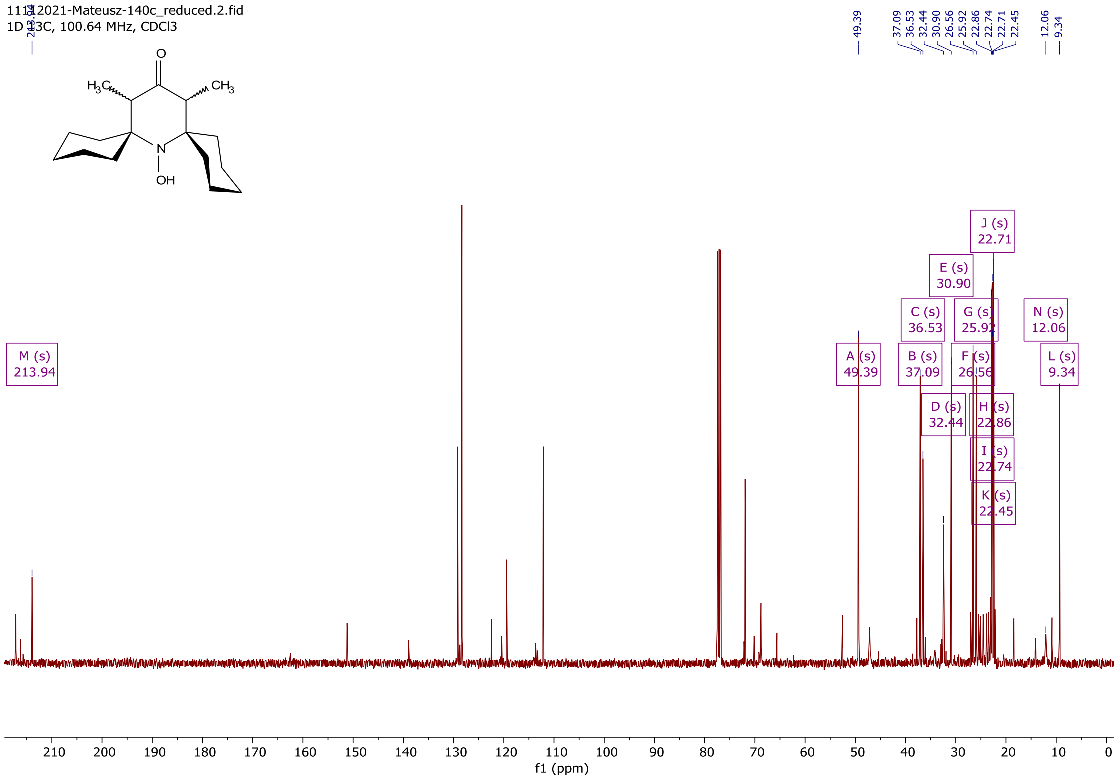

^{13}C NMR (101 MHz, $CDCl_3$) δ 213.94, 49.39, 37.09, 36.53, 32.44, 30.90, 26.56, 25.92, 22.86, 22.74, 22.71, 22.45, 12.06, 9.34.

*Figure S30. ^{13}C NMR spectrum of **8'***

9’

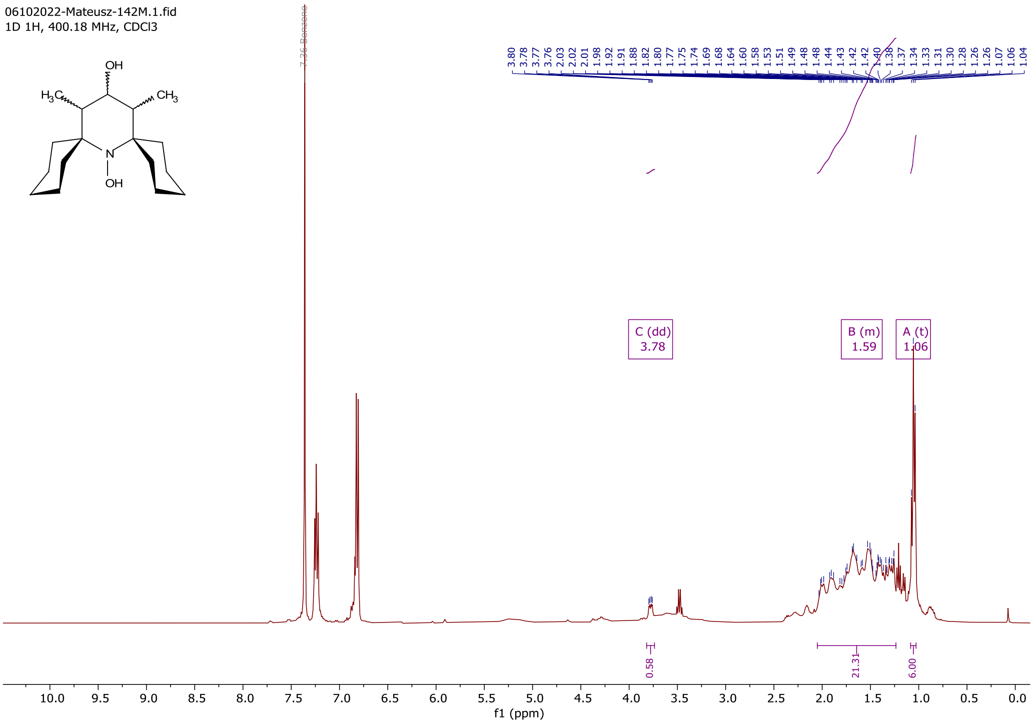

1H NMR (400 MHz, $CDCl_3$) δ 3.78 (1H, dd, *J* = 10.0, 4.4 Hz), 2.05–1.24 (21H, m), 1.06 (6H, t, *J* = 7.5 Hz).

*Figure S31. ¹H NMR spectrum of **9’**.*

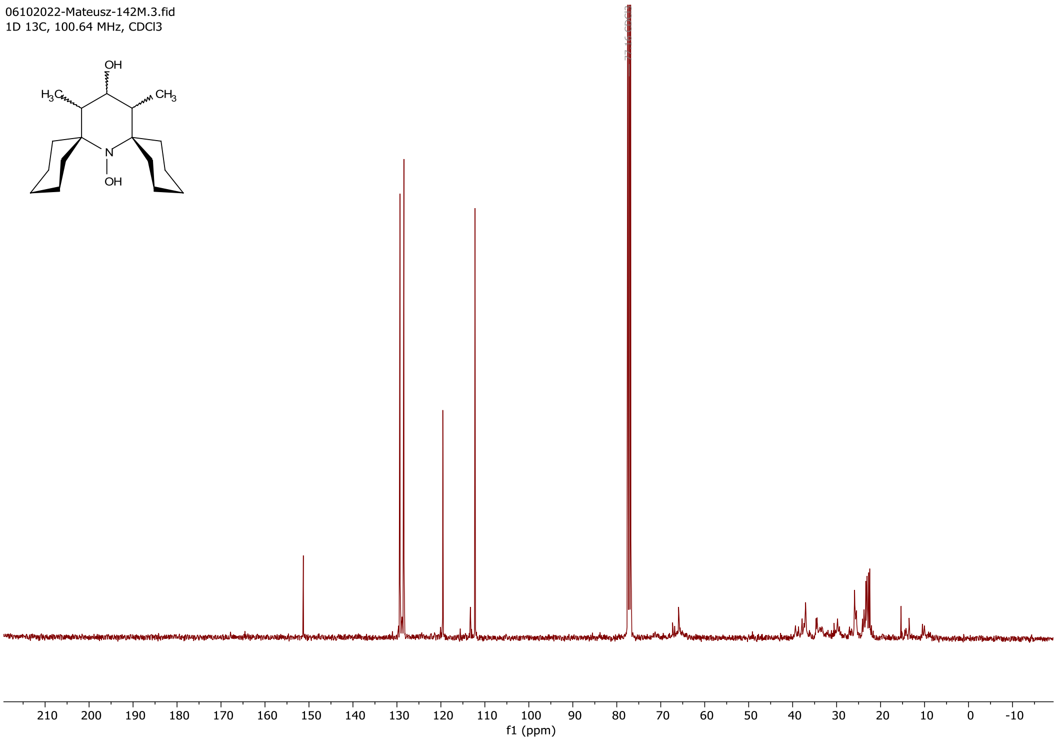

*Figure S32. ¹³C NMR spectrum of **9’**. Significant line-broadening is apparent in the NMR spectra of **9’**, presumably due to incomplete reduction.*

10’

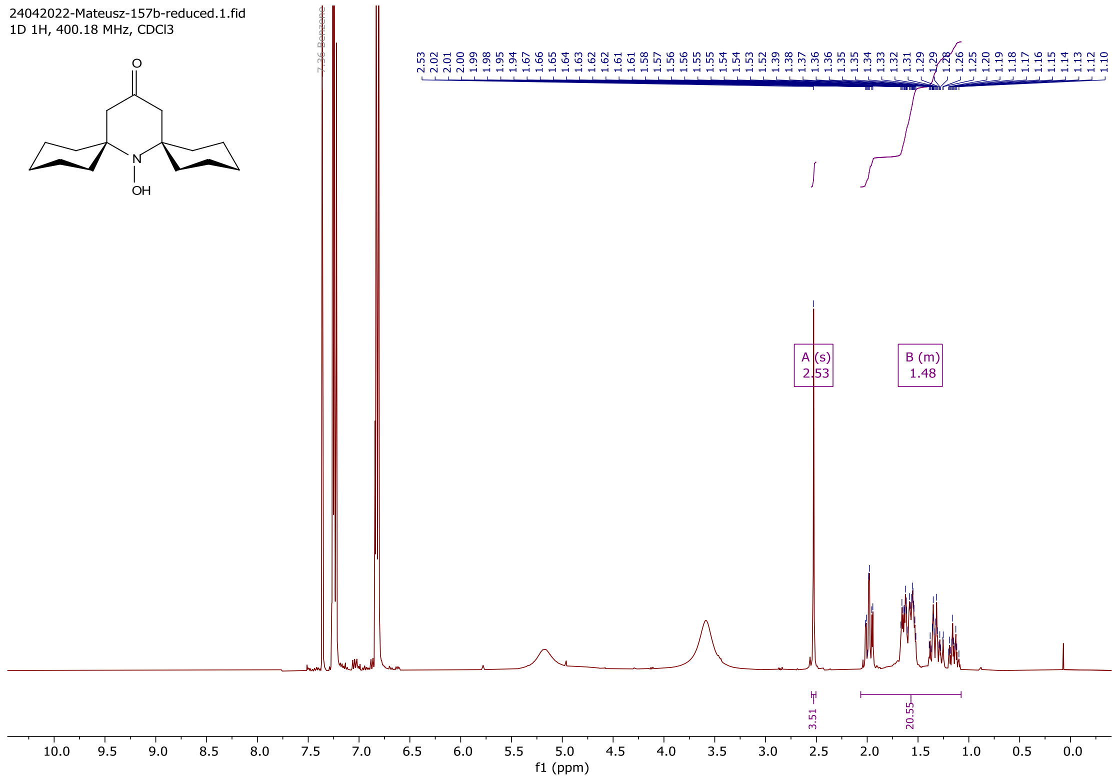

1H NMR (400 MHz, $CDCl_3$) δ 2.53 (4H, s), 2.06–1.08 (21H, m).

*Figure S33. 1H NMR spectrum of **10’**.*

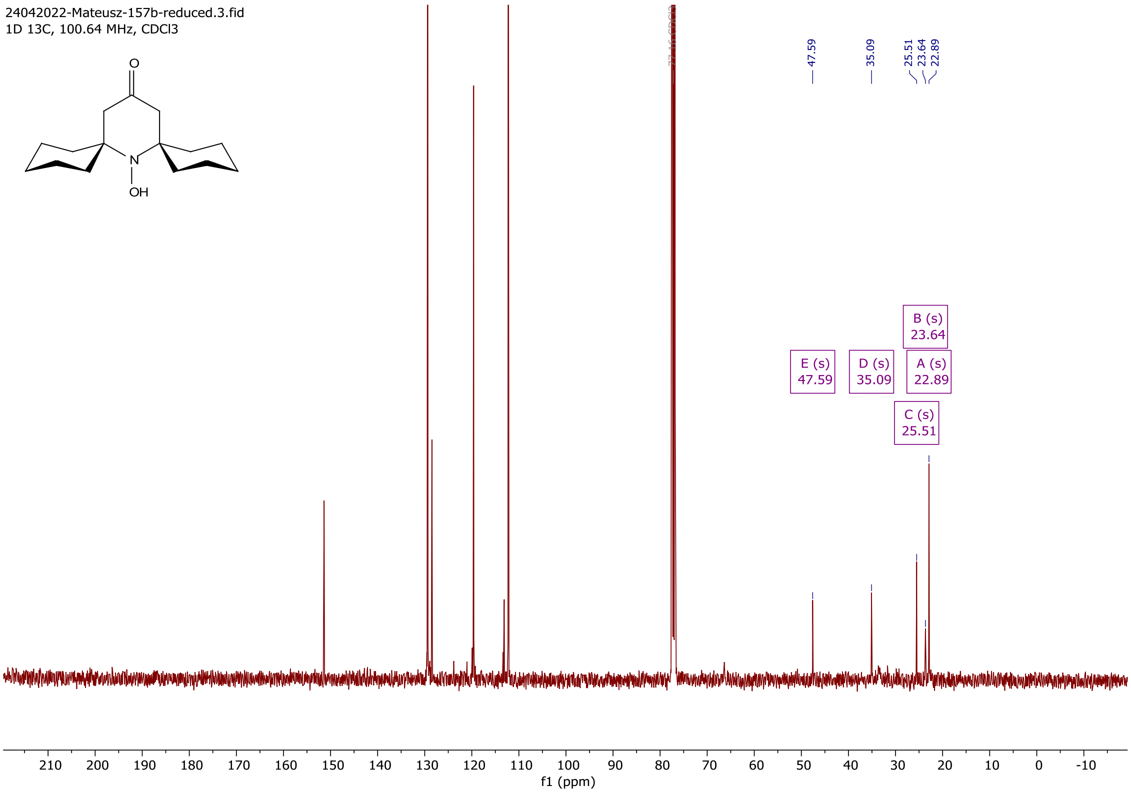

13C NMR (101 MHz, $CDCl_3$) δ 47.59, 35.09, 25.51, 23.64, 22.89.

*Figure S34. 13C NMR spectrum of **10’**.*

11’

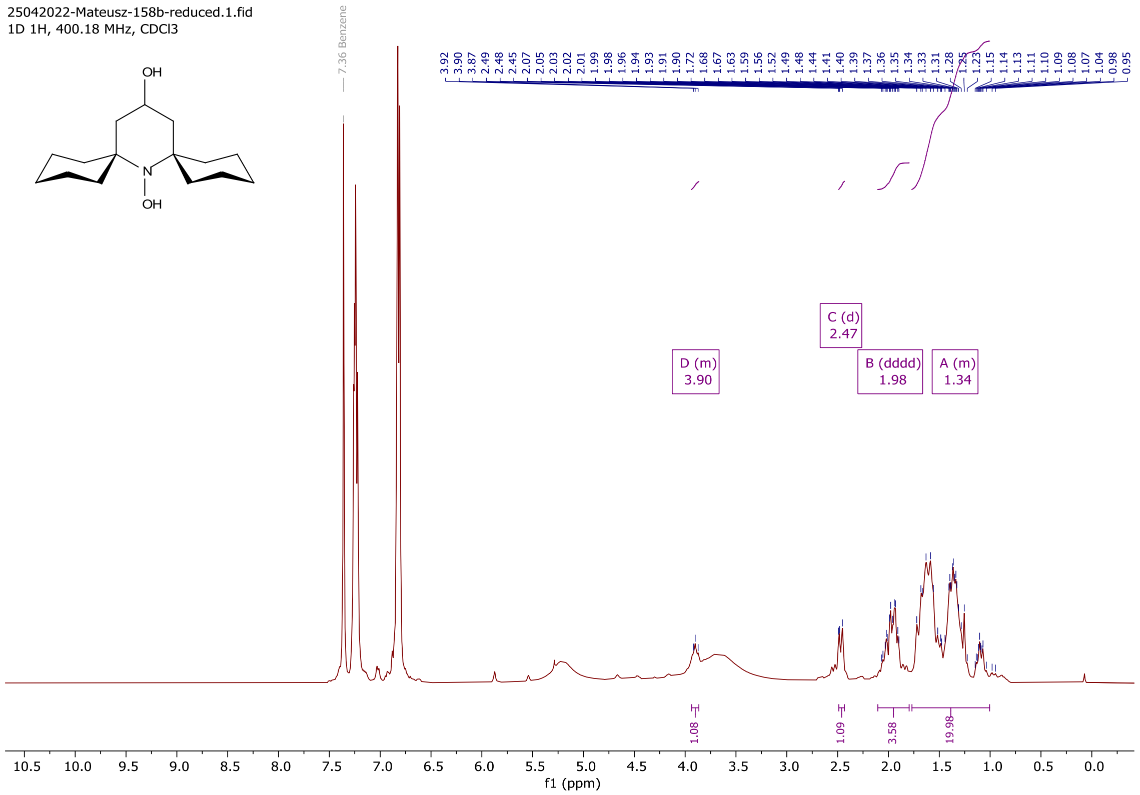

^{1}H NMR (400 MHz, $CDCl_3$) δ 3.94–3.87 (1H, m), 2.47 (1H, d, J = 11.1 Hz), 1.98 (4H, dddd, J = 32.7, 18.6, 13.5, 4.7 Hz), 1.77–1.01 (20H, m).

*Figure S35. ^{1}H NMR spectrum of **11’**.*

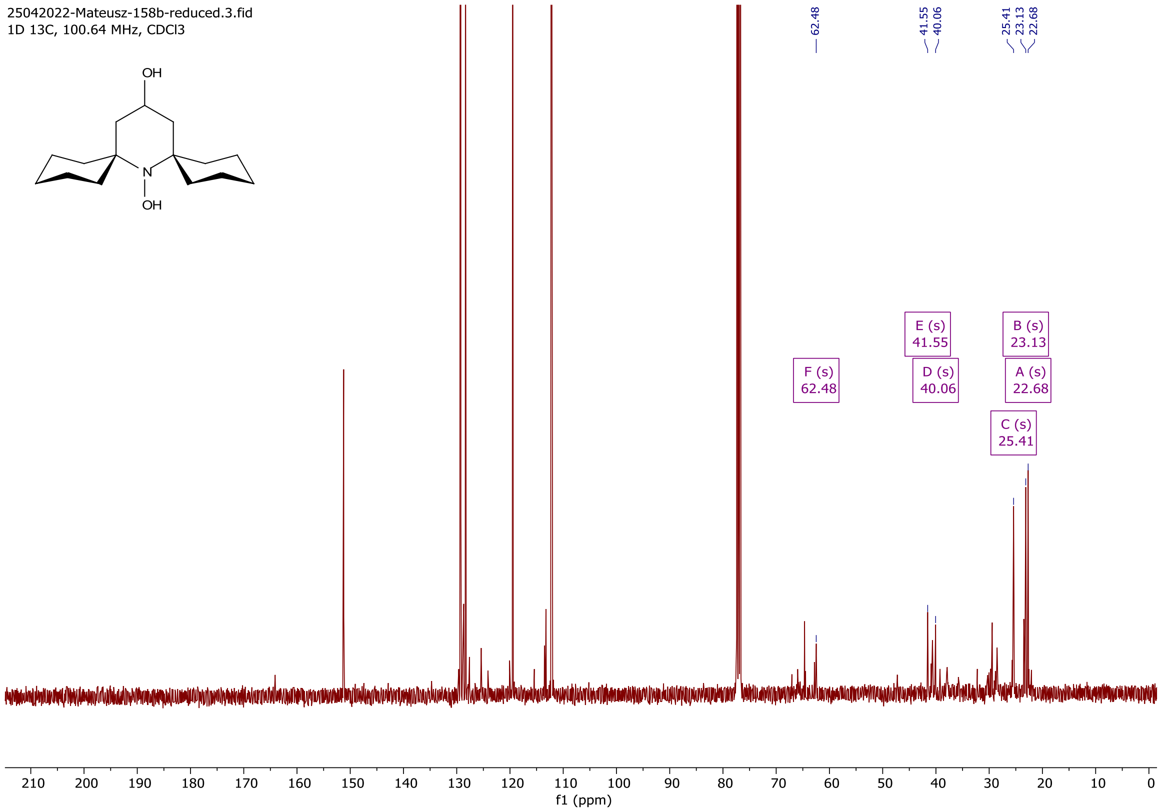

^{13}C NMR (101 MHz, $CDCl_3$) δ 62.48, 41.55, 40.06, 25.41, 23.13, 22.68.

*Figure S36. ^{13}C NMR spectrum of **11’**.*

14’

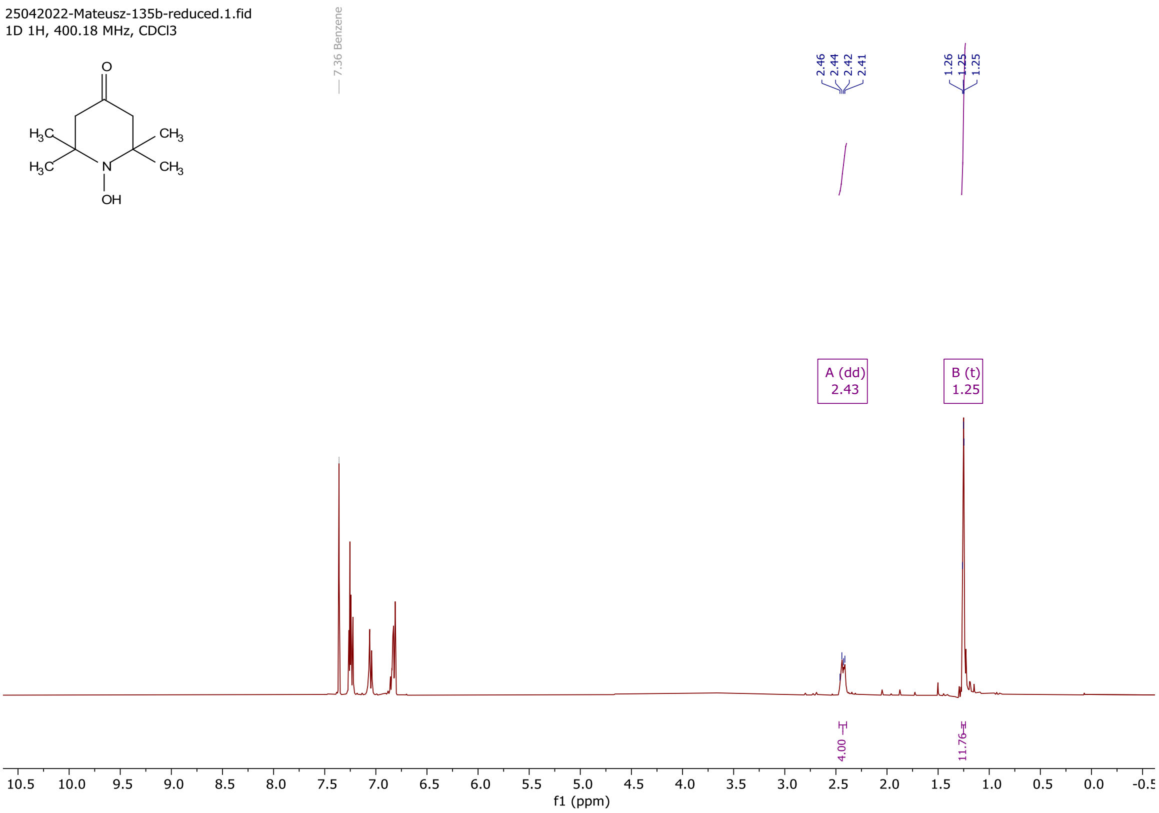

^{1}H NMR (400 MHz, CDCl$_3$) δ 2.43 (4H, dd, J = 13.0, 6.2 Hz), 1.25 (12H, t, J = 2.8 Hz).

*Figure S37. ^{1}H NMR spectrum of **14’**.*

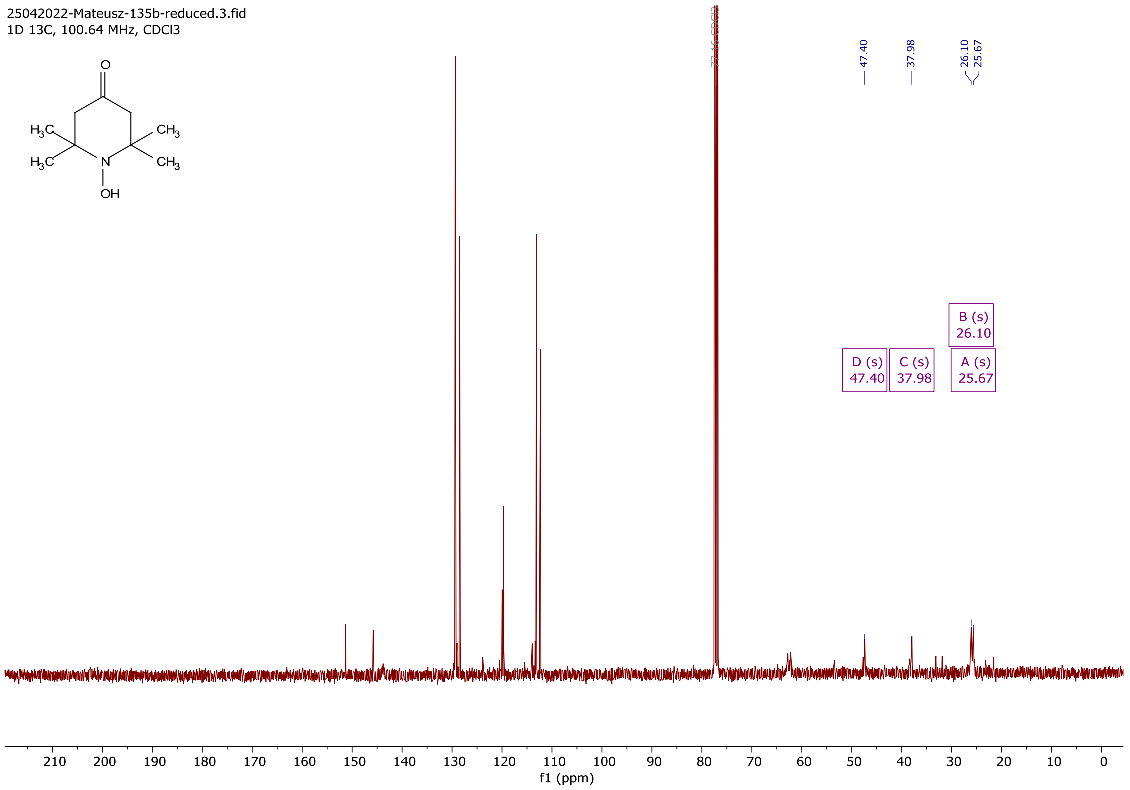

^{13}C NMR (101 MHz, CDCl$_3$) δ 47.40, 37.98, 26.10, 25.67.

*Figure S38. ^{13}C NMR spectrum of **14’**.*

12’

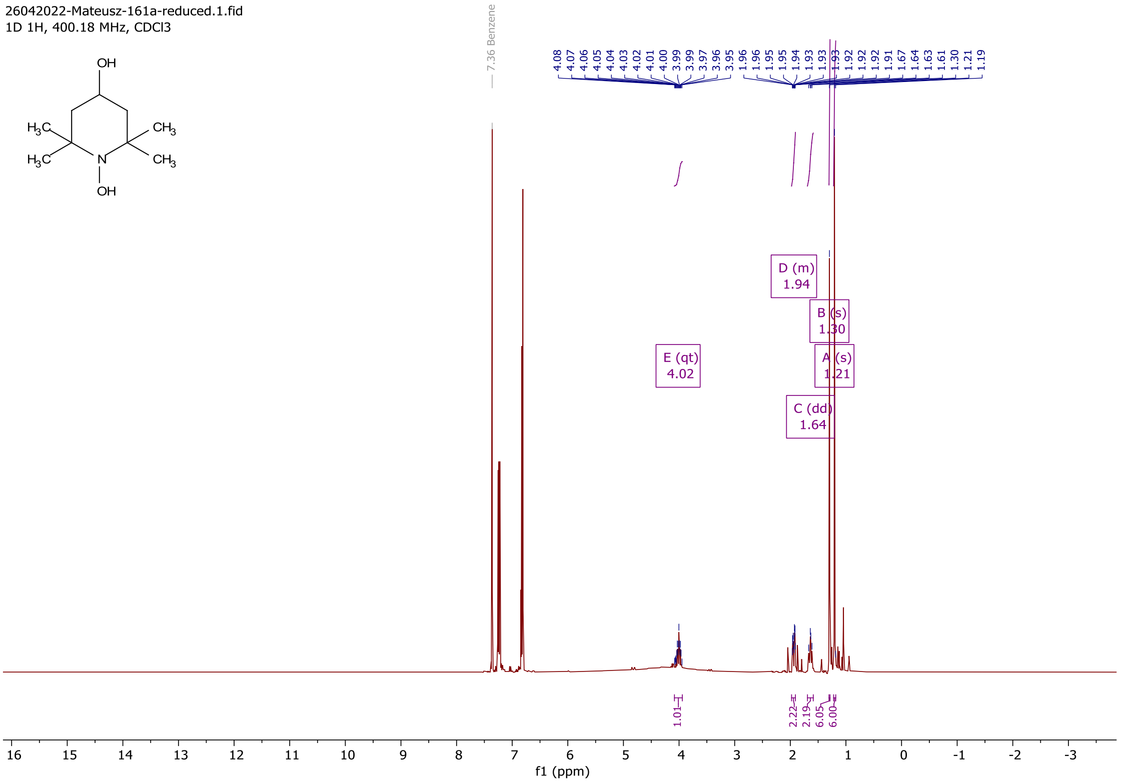

^{1}H NMR (400 MHz, $CDCl_3$) δ 4.02 (1H, qt, J = 11.4, 4.3 Hz), 1.98–1.91 (2H, m), 1.64 (2H, dd, J = 13.8, 10.1 Hz), 1.30 (6H, s), 1.21 (6H, s).

*Figure S39. ^{1}H NMR spectrum of **12’**.*

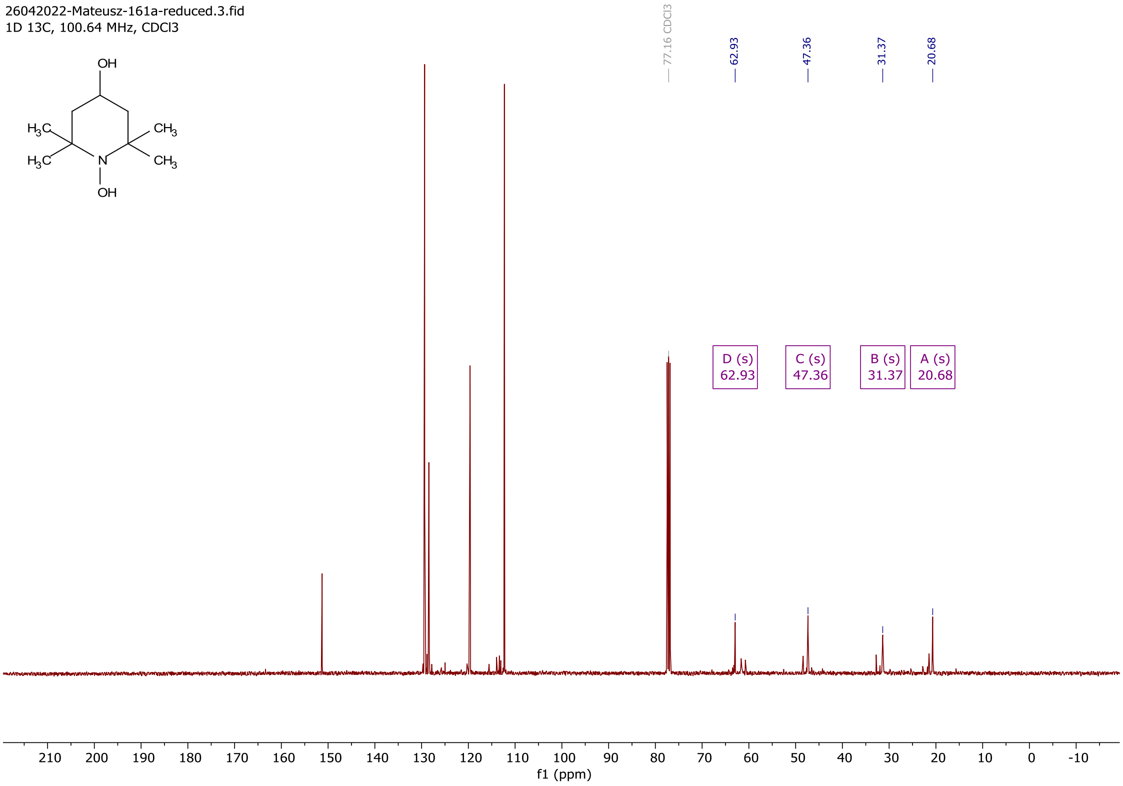

^{13}C NMR (101 MHz, $CDCl_3$) δ 62.93, 47.36, 31.37, 20.68.

*Figure S40. ^{13}C NMR spectrum of **12’**.*

15

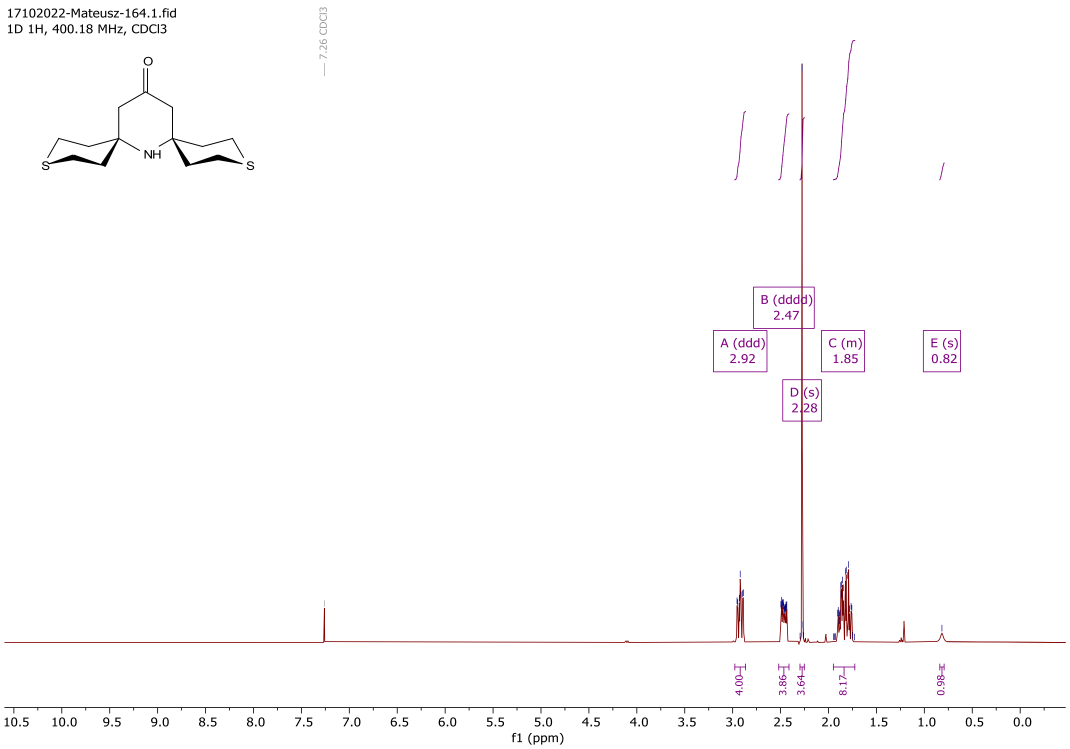

^{1}H NMR (400 MHz, $CDCl_3$) δ 2.92 (4H, ddd, *J* = 13.2, 9.6, 3.1 Hz), 2.47 (4H, dddd, *J* = 13.4, 6.7, 3.4, 1.1 Hz), 2.28 (4H, s), 1.95–1.73 (8H, m), 0.82 (1H, s).

*Figure S41. ^{1}H NMR spectrum of **15**.*

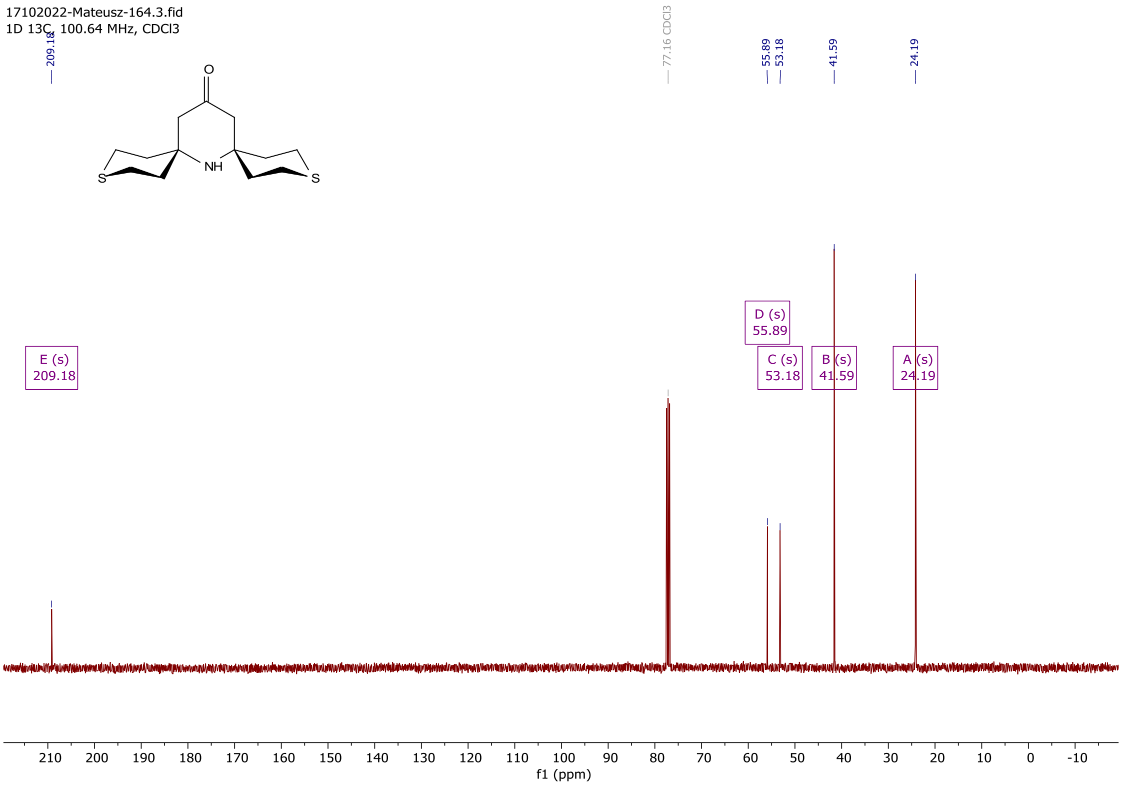

^{13}C NMR (101 MHz, $CDCl_3$) δ 209.18, 55.89, 53.18, 41.59, 24.19.

*Figure S42. ^{13}C NMR spectrum of **15**.*

16

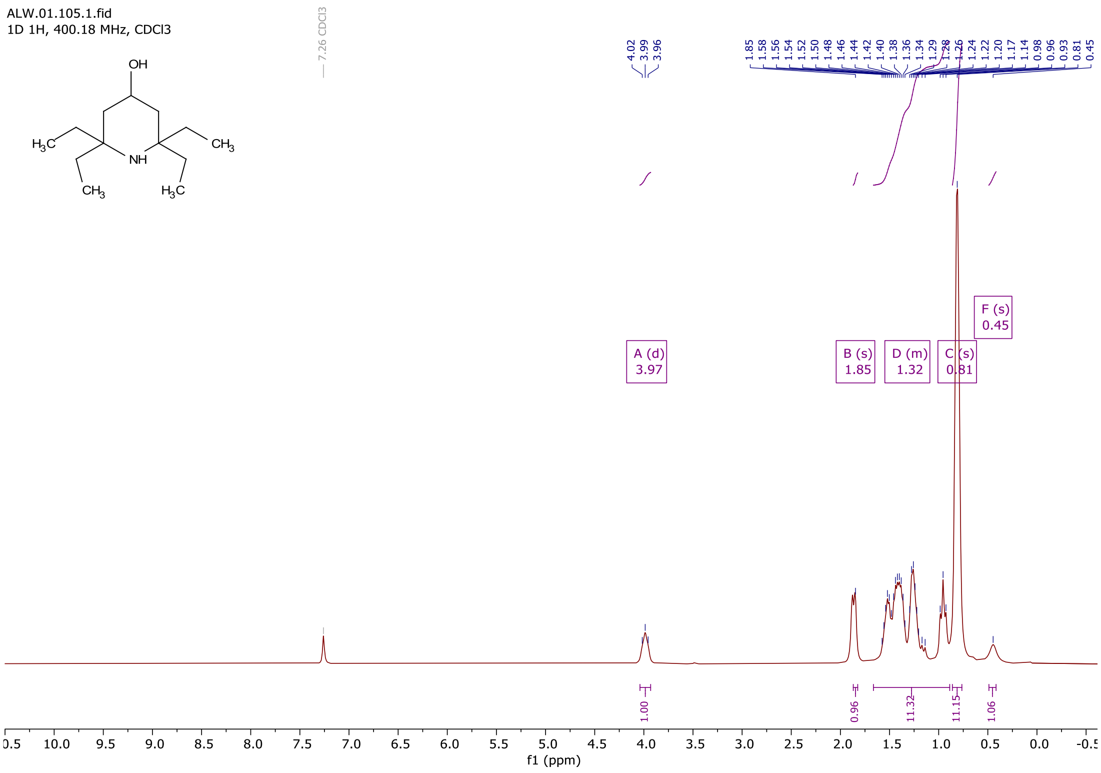

^{1}**H NMR** (400 MHz, $CDCl_3$) δ 3.97 (1H, d, *J* = 11.9 Hz), 1.85 (1H, s), 1.66–0.89 (11H, m), 0.81 (11H, s), 0.45 (1H, s).

*Figure S43. ^{1}H NMR spectrum of **16**.*

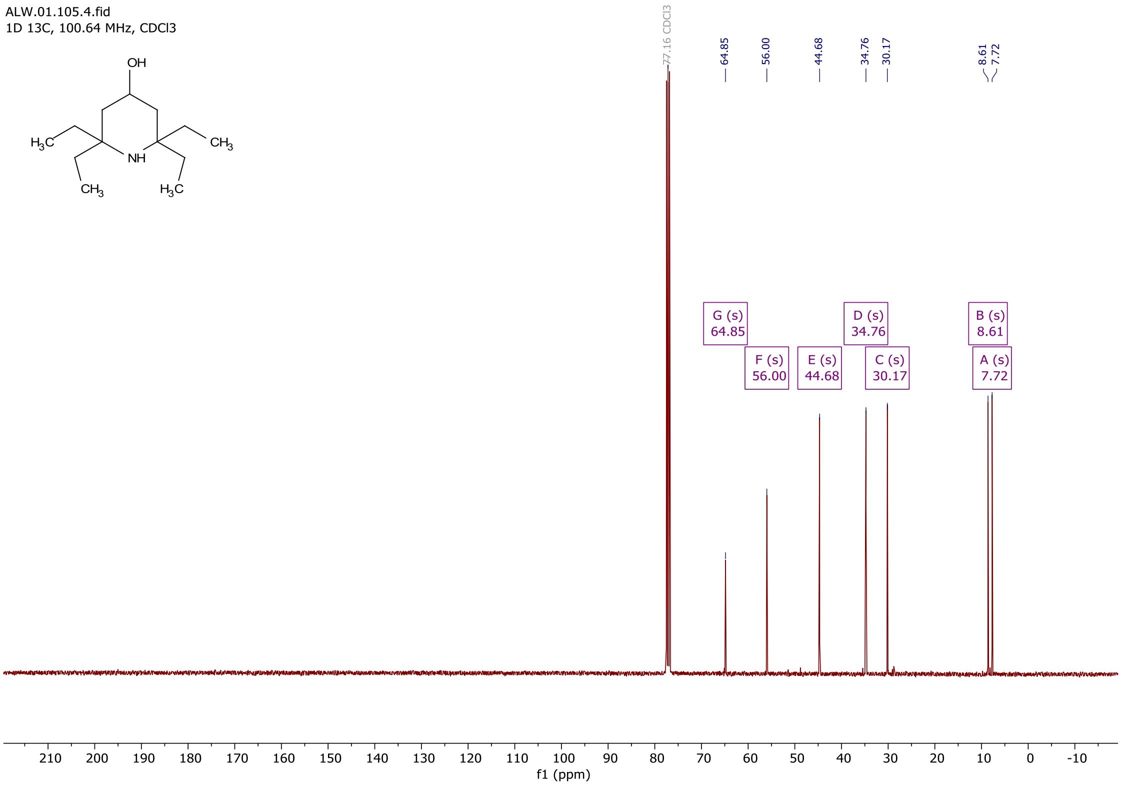

^{13}**C NMR** (101 MHz, $CDCl_3$) δ 64.85, 56.00, 44.68, 34.76, 30.17, 8.61, 7.72.

*Figure S44. ^{13}C NMR spectrum of **16**.*

13’

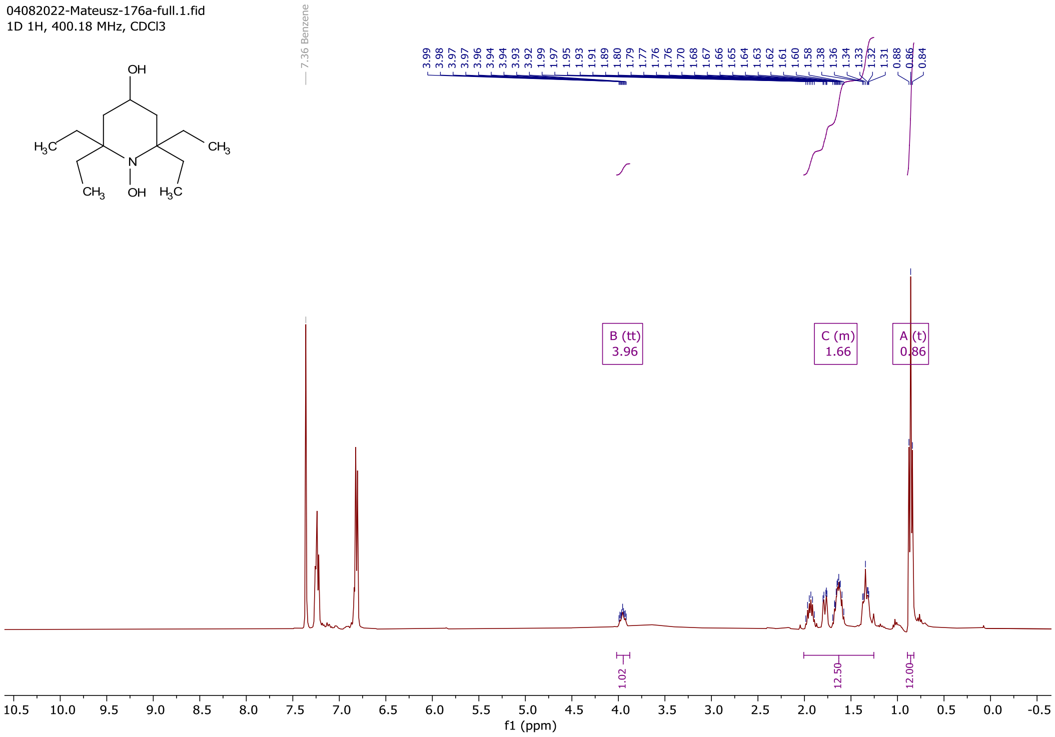

^{1}**H NMR** (400 MHz, $CDCl_3$) δ 3.96 (1H, tt, *J* = 11.5, 4.0 Hz), 2.01–1.25 (12H, m), 0.86 (12H, t, *J* = 7.5 Hz).

*Figure S45. ^{1}H NMR spectrum of **13’**.*

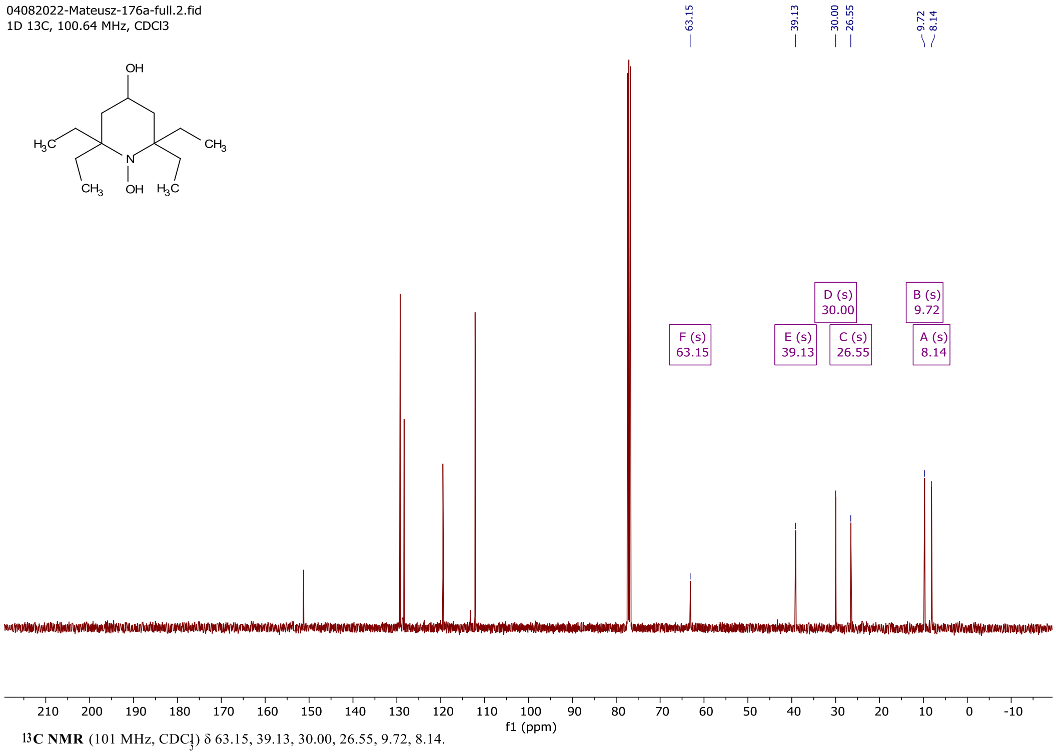

^{13}**C NMR** (101 MHz, $CDCl_3$) δ 63.15, 39.13, 30.00, 26.55, 9.72, 8.14.

*Figure S46. ^{13}C NMR spectrum of **13’**.*
