# Supplementary material for: Conformational tuning improves the stability of spirocyclic nitroxides with long paramagnetic relaxation times
Source: Commun Chem. 2023 Jun 5;6:111. doi: 10.1038/s42004-023-00912-7 (PMC10241799; doi:10.1038/s42004-023-00912-7)
Supplement: Supplementary file 6 — Supplementary Data 3 [file 42004_2023_912_MOESM6_ESM.pdf]

Crystal Structure Report for 211119MS114overweekend

A clear light orange plate-like specimen of $C_{2.13}H_{3.47}N_{0.13}O_{0.27}$, approximate dimensions 0.050 mm x 0.100 mm x 0.100 mm, was used for the X-ray crystallographic analysis. The X-ray intensity data were measured (λ = 1.54178 Å).

Table 1: Data collection details for 211119MS114overweekend.

Axis	dx/mm	2θ/°	ω/°	φ/°	χ/°	Width/°	Frames	Time/s	Wavelength/Å	Voltage/kV	Current/mA	Temperature/K
Phi	37.060	91.28	91.67	0.00	-44.50	1.90	189	95.40	1.54184	50	1.1	303
Omega	37.060	50.04	-37.97	51.00	65.50	1.90	49	60.00	1.54184	50	1.1	303
Omega	37.060	61.28	61.67	270.00	-44.50	1.90	49	65.70	1.54184	50	1.1	303
Omega	37.060	106.28	-9.01	0.00	65.50	1.90	51	120.00	1.54184	50	1.1	303
Phi	37.060	106.28	14.21	-182.60	22.00	1.90	163	120.00	1.54184	50	1.1	303
Omega	37.060	106.28	-20.52	120.00	80.00	1.90	47	120.00	1.54184	50	1.1	303
Omega	37.060	61.28	61.67	180.00	-44.50	1.90	49	65.70	1.54184	50	1.1	303
Omega	37.060	50.04	-37.97	-156.00	65.50	1.90	49	60.00	1.54184	50	1.1	303
Omega	37.060	106.28	-20.52	40.00	80.00	1.90	47	120.00	1.54184	50	1.1	303
Omega	37.060	106.28	-20.52	-160.00	80.00	1.90	47	120.00	1.54184	50	1.1	303
Omega	37.060	76.28	76.67	270.00	-44.50	1.90	41	77.70	1.54184	50	1.1	303
Omega	37.060	106.28	-20.52	160.00	80.00	1.90	47	120.00	1.54184	50	1.1	303
Omega	37.060	76.28	76.67	90.00	-44.50	1.90	41	77.70	1.54184	50	1.1	303
Omega	37.060	106.28	-20.52	80.00	80.00	1.90	47	120.00	1.54184	50	1.1	303
Phi	37.060	106.28	109.92	-20.00	-22.00	1.90	97	120.00	1.54184	50	1.1	303
Omega	37.060	106.28	-20.52	-120.00	80.00	1.90	47	120.00	1.54184	50	1.1	303
Omega	37.060	50.04	-37.97	153.00	65.50	1.90	49	60.00	1.54184	50	1.1	303
Omega	37.060	76.28	76.67	0.00	-44.50	1.90	41	77.70	1.54184	50	1.1	303
Omega	37.060	50.04	52.04	102.00	-44.50	1.90	53	60.00	1.54184	50	1.1	303
Omega	37.060	106.28	-20.52	-40.00	80.00	1.90	47	120.00	1.54184	50	1.1	303
Omega	37.060	76.28	76.67	180.00	-44.50	1.90	41	77.70	1.54184	50	1.1	303
Omega	37.060	50.04	-37.97	-105.00	65.50	1.90	49	60.00	1.54184	50	1.1	303
Omega	37.060	106.28	-9.01	120.00	65.50	1.90	51	120.00	1.54184	50	1.1	303
Omega	37.060	106.28	-20.52	-80.00	80.00	1.90	47	120.00	1.54184	50	1.1	303

A total of 1438 frames were collected. The total exposure time was 39.09 hours. The frames were integrated with the Bruker SAINT software package using a narrow-frame algorithm. The integration of the data using a monoclinic unit cell yielded a total of 26191 reflections to a maximum θ angle of 67.03° (0.84 Å resolution), of which 2617 were independent (average redundancy 10.008, completeness = 97.7%, R_{int} = 14.05%, R_{sig} = 6.01%) and 1744 (66.64%) were greater than $2\sigma(F^2)$. The final cell constants of a = 6.0349(8) Å, b = 21.053(3) Å, c = 12.0259(15) Å, β = 100.896(7)°, volume = 1500.4(3) Å^3, are based upon the refinement of the XYZ-centroids of 6749 reflections above 20 σ(I) with 8.585° < 2θ < 133.0°. Data were corrected for absorption effects using the Multi-Scan method (SADABS). The ratio of minimum to maximum apparent transmission was 0.726. The calculated minimum and maximum transmission coefficients (based on crystal size) are 0.9450 and 0.9720.

The structure was solved and refined using the Bruker SHELXTL Software Package, using the space group P 1 21/n 1, with Z = 30 for the formula unit, $C_{2.13}H_{3.47}N_{0.13}O_{0.27}$. The final anisotropic full-matrix least-squares refinement on F^2 with 176 variables converged at R1 = 9.98%, for the observed data and wR2 = 19.04% for all data. The goodness-of-fit was 1.230. The largest peak in the final difference electron density synthesis was 0.225 $e^-/Å^3$ and the largest hole was -0.212 $e^-/Å^3$ with an RMS deviation of 0.043 $e^-/Å^3$. On the basis of the final model, the calculated density was 1.170 g/cm^3 and F(000), 580 e^-.

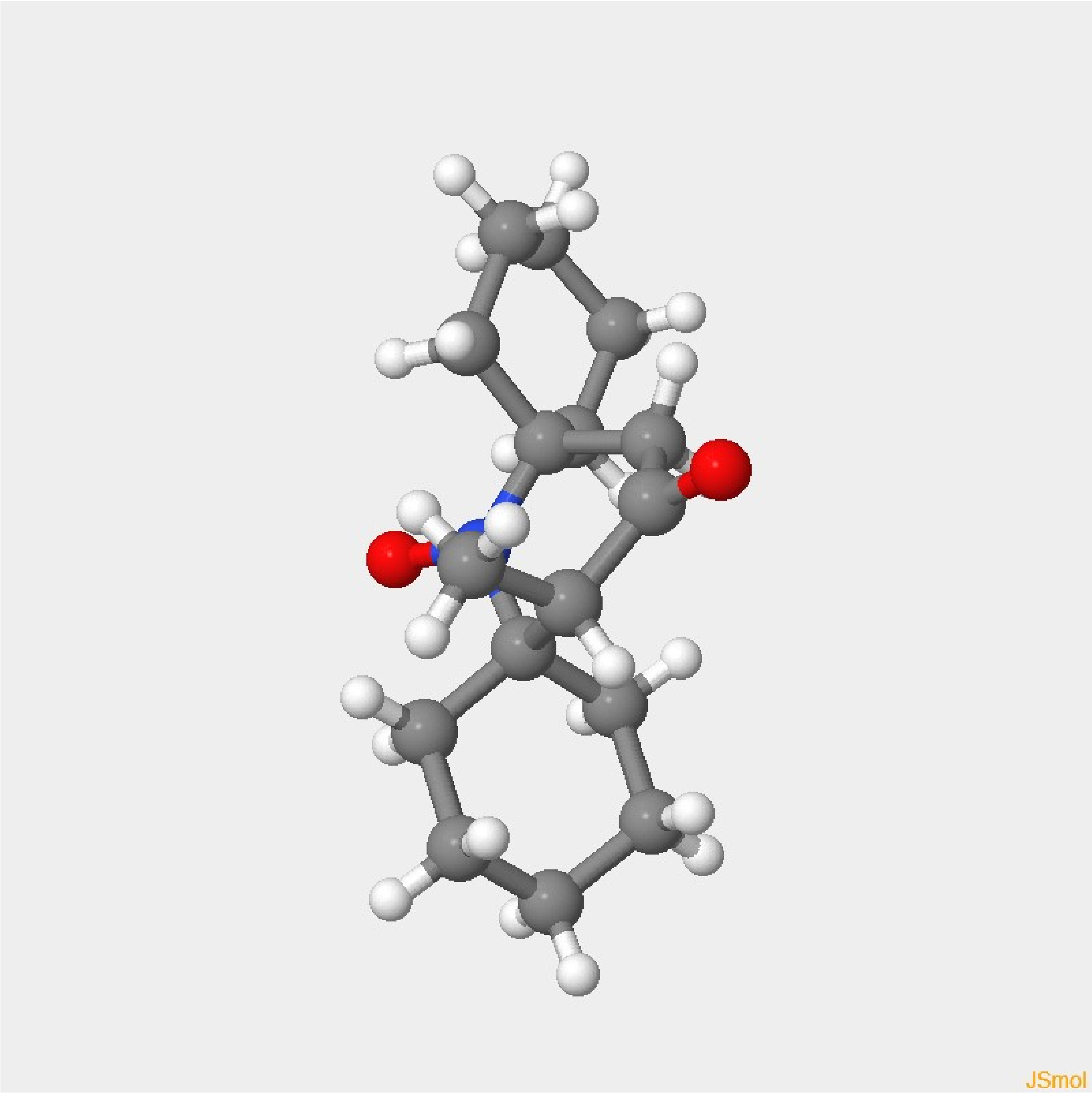
JSmol

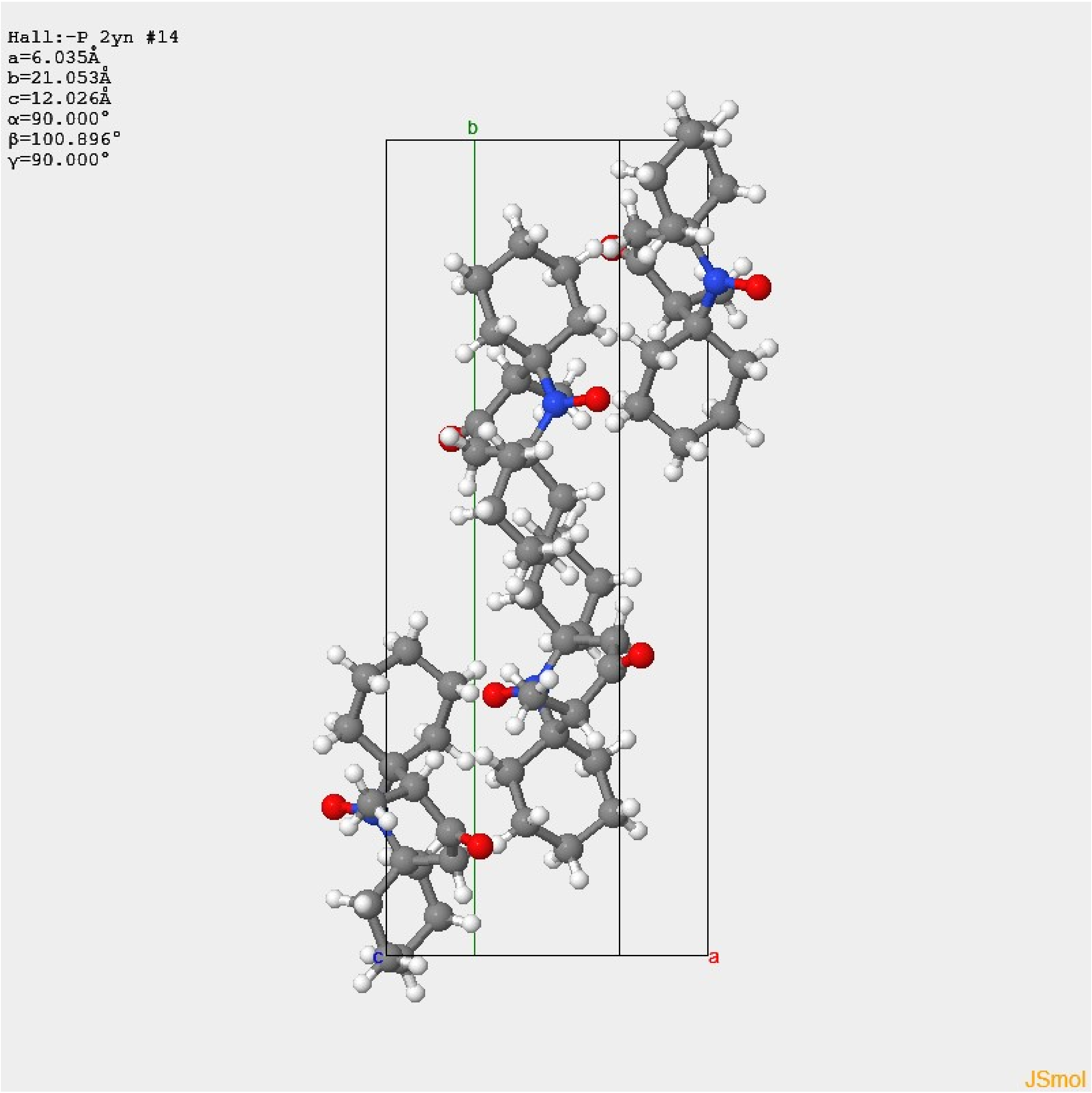

Table 2. Sample and crystal data for 211119MS114overweekend.

**Identification code**	211119MS114overweekend	
**Chemical formula**	$C_{2.13}H_{3.47}N_{0.13}O_{0.27}$	
**Formula weight**	35.25 g/mol	
**Temperature**	303(2) K	
**Wavelength**	1.54178 Å	
**Crystal size**	0.050 x 0.100 x 0.100 mm	
**Crystal habit**	clear light orange plate	
**Crystal system**	monoclinic	
**Space group**	P 1 21/n 1	
**Unit cell dimensions**	a = 6.0349(8) Å	α = 90°
	b = 21.053(3) Å	β = 100.896(7)°
	c = 12.0259(15) Å	γ = 90°
**Volume**	1500.4(3) $Å^3$	
**Z**	30	

**Density (calculated)**	1.170 g/cm^3
**Absorption coefficient**	0.572 mm^{-1}
**F(000)**	580

Table 3. Data collection and structure refinement for 211119MS114overweekend.

**Theta range for data collection**	4.29 to 67.03°	
**Index ranges**	-7<=h<=7, -24<=k<=25, -14<=l<=14	
**Reflections collected**	26191	
**Independent reflections**	2617 [R(int) = 0.1405]	
**Coverage of independent reflections**	97.7%	
**Absorption correction**	Multi-Scan	
**Max. and min. transmission**	0.9720 and 0.9450	
**Structure solution technique**	direct methods	
**Structure solution program**	SHELXT 2014/5 (Sheldrick, 2014)	
**Refinement method**	Full-matrix least-squares on F^2	
**Refinement program**	SHELXL-2018/3 (Sheldrick, 2018)	
**Function minimized**	Σ w(F$_o^2$ - F$_c^2$)^2	
**Data / restraints / parameters**	2617 / 0 / 176	
**Goodness-of-fit on F^2**	1.230	
**Final R indices**	1744 data; I>2σ(I)	R1 = 0.0998, wR2 = 0.1760
	all data	R1 = 0.1382, wR2 = 0.1904
**Weighting scheme**	w=1/[σ^2(F$_o^2$)+2.3739P]<br>where P=(F$_o^2$+2F$_c^2$)/3	
**Largest diff. peak and hole**	0.225 and -0.212 eÅ^{-3}	
**R.M.S. deviation from mean**	0.043 eÅ^{-3}	

Table 4. Atomic coordinates and equivalent isotropic atomic displacement parameters (Å^2) for 211119MS114overweekend.

U(eq) is defined as one third of the trace of the orthogonalized U$_{ij}$ tensor.

	x/a	y/b	z/c	U(eq)
O001	0.1956(5)	0.31822(16)	0.2717(3)	0.0695(13)
N1	0.3932(6)	0.32514(17)	0.3333(3)	0.0482(12)
O003	0.9328(6)	0.3668(2)	0.5756(3)	0.0906(14)
C004	0.5010(6)	0.26958(19)	0.3990(3)	0.0413(13)
C005	0.6316(7)	0.2955(2)	0.5128(3)	0.0472(14)
C006	0.7820(7)	0.3503(2)	0.4981(4)	0.0550(15)
C007	0.6552(7)	0.2356(2)	0.3294(3)	0.0508(16)
C008	0.3146(7)	0.2236(2)	0.4154(4)	0.0528(16)
C009	0.5128(7)	0.3855(2)	0.3156(3)	0.0481(14)
C00A	0.4070(8)	0.1614(2)	0.4708(4)	0.0602(16)
C00B	0.4819(8)	0.3161(3)	0.5957(4)	0.0692(17)
C00C	0.5244(9)	0.3902(2)	0.1902(4)	0.0666(17)
C00D	0.3712(9)	0.4398(2)	0.3482(4)	0.0656(18)
C00E	0.7438(8)	0.1722(2)	0.3799(4)	0.0601(16)
C00F	0.5565(8)	0.1285(2)	0.4002(4)	0.0644(16)
C00G	0.7508(8)	0.3842(2)	0.3878(4)	0.0649(17)
C00H	0.4422(11)	0.5049(2)	0.3127(5)	0.083(2)
C00I	0.6025(11)	0.4551(3)	0.1583(5)	0.084(2)
C00J	0.4503(11)	0.5065(3)	0.1873(5)	0.090(2)

Table 5. Bond lengths (Å) for 211119MS114overweekend.

O001-N1	1.288(5)	N1-C004	1.490(5)
N1-C009	1.496(5)	O003-C006	1.224(5)
C004-C008	1.525(5)	C004-C007	1.540(5)
C004-C005	1.544(5)	C005-C006	1.499(6)
C005-C00B	1.528(6)	C005-H005	0.98
C006-C00G	1.487(6)	C007-C00E	1.522(6)
C007-H00A	0.97	C007-H00B	0.97
C008-C00A	1.525(6)	C008-H00C	0.97
C008-H00D	0.97	C009-C00D	1.522(6)
C009-C00C	1.526(6)	C009-C00G	1.532(6)
C00A-C00F	1.518(6)	C00A-H00E	0.97
C00A-H00F	0.97	C00B-H00G	0.96
C00B-H00H	0.96	C00B-H00I	0.96
C00C-C00I	1.519(7)	C00C-H00J	0.97
C00C-H00K	0.97	C00D-C00H	1.520(7)
C00D-H00L	0.97	C00D-H00M	0.97
C00E-C00F	1.511(6)	C00E-H00N	0.97
C00E-H00O	0.97	C00F-H00P	0.97
C00F-H00Q	0.97	C00G-H00R	0.97
C00G-H00S	0.97	C00H-C00J	1.519(7)
C00H-H00T	0.97	C00H-H00U	0.97
C00I-C00J	1.502(7)	C00I-H00V	0.97
C00I-H00W	0.97	C00J-H00X	0.97
C00J-H00Y	0.97		

Table 6. Bond angles (°) for 211119MS114overweekend.

O001-N1-C004	118.7(3)	O001-N1-C009	115.7(3)
C004-N1-C009	124.6(3)	N1-C004-C008	108.0(3)
N1-C004-C007	108.9(3)	C008-C004-C007	108.2(3)
N1-C004-C005	106.9(3)	C008-C004-C005	112.0(3)
C007-C004-C005	112.7(3)	C006-C005-C00B	107.7(4)
C006-C005-C004	112.7(3)	C00B-C005-C004	114.4(3)
C006-C005-H005	107.2	C00B-C005-H005	107.2
C004-C005-H005	107.2	O003-C006-C00G	119.6(4)
O003-C006-C005	120.6(4)	C00G-C006-C005	119.9(4)
C00E-C007-C004	113.2(3)	C00E-C007-H00A	108.9
C004-C007-H00A	108.9	C00E-C007-H00B	108.9
C004-C007-H00B	108.9	H00A-C007-H00B	107.8
C004-C008-C00A	112.6(3)	C004-C008-H00C	109.1
C00A-C008-H00C	109.1	C004-C008-H00D	109.1
C00A-C008-H00D	109.1	H00C-C008-H00D	107.8
N1-C009-C00D	107.0(3)	N1-C009-C00C	108.0(3)
C00D-C009-C00C	109.8(4)	N1-C009-C00G	109.3(3)
C00D-C009-C00G	112.3(4)	C00C-C009-C00G	110.4(4)
C00F-C00A-C008	110.8(4)	C00F-C00A-H00E	109.5
C008-C00A-H00E	109.5	C00F-C00A-H00F	109.5
C008-C00A-H00F	109.5	H00E-C00A-H00F	108.1
C005-C00B-H00G	109.5	C005-C00B-H00H	109.5
H00G-C00B-H00H	109.5	C005-C00B-H00I	109.5
H00G-C00B-H00I	109.5	H00H-C00B-H00I	109.5
C00I-C00C-C009	112.5(4)	C00I-C00C-H00J	109.1

C009-C00C-H00J	109.1	C00I-C00C-H00K	109.1
C009-C00C-H00K	109.1	H00J-C00C-H00K	107.8
C00H-C00D-C009	113.8(4)	C00H-C00D-H00L	108.8
C009-C00D-H00L	108.8	C00H-C00D-H00M	108.8
C009-C00D-H00M	108.8	H00L-C00D-H00M	107.7
C00F-C00E-C007	112.4(4)	C00F-C00E-H00N	109.1
C007-C00E-H00N	109.1	C00F-C00E-H00O	109.1
C007-C00E-H00O	109.1	H00N-C00E-H00O	107.9
C00E-C00F-C00A	110.7(4)	C00E-C00F-H00P	109.5
C00A-C00F-H00P	109.5	C00E-C00F-H00Q	109.5
C00A-C00F-H00Q	109.5	H00P-C00F-H00Q	108.1
C006-C00G-C009	117.4(4)	C006-C00G-H00R	107.9
C009-C00G-H00R	107.9	C006-C00G-H00S	107.9
C009-C00G-H00S	107.9	H00R-C00G-H00S	107.2
C00J-C00H-C00D	111.2(4)	C00J-C00H-H00T	109.4
C00D-C00H-H00T	109.4	C00J-C00H-H00U	109.4
C00D-C00H-H00U	109.4	H00T-C00H-H00U	108.0
C00J-C00I-C00C	110.9(4)	C00J-C00I-H00V	109.5
C00C-C00I-H00V	109.5	C00J-C00I-H00W	109.5
C00C-C00I-H00W	109.5	H00V-C00I-H00W	108.0
C00I-C00J-C00H	110.5(5)	C00I-C00J-H00X	109.5
C00H-C00J-H00X	109.5	C00I-C00J-H00Y	109.5
C00H-C00J-H00Y	109.5	H00X-C00J-H00Y	108.1

Table 7. Anisotropic atomic displacement parameters (Å^2) for 211119MS114overweekend.

The anisotropic atomic displacement factor exponent takes the form: $-2\pi^2[h^2 a^{*2} U_{11} + ... + 2 h k a^* b^* U_{12}]$

	U_{11}	U_{22}	U_{33}	U_{23}	U_{13}	U_{12}
O001	0.048(2)	0.080(3)	0.070(2)	0.0130(18)	-0.0156(16)	-0.0049(16)
N1	0.043(2)	0.059(2)	0.042(2)	0.0051(17)	0.0075(16)	0.0003(17)
O003	0.073(3)	0.115(3)	0.072(3)	-0.002(2)	-0.016(2)	-0.022(2)
C004	0.038(2)	0.052(3)	0.034(2)	0.0023(18)	0.0069(17)	0.0000(18)
C005	0.046(3)	0.062(3)	0.034(2)	0.0022(19)	0.0062(18)	0.002(2)
C006	0.046(3)	0.076(3)	0.042(3)	-0.005(2)	0.005(2)	0.003(2)
C007	0.057(3)	0.056(3)	0.042(3)	0.001(2)	0.016(2)	-0.002(2)
C008	0.040(3)	0.070(3)	0.049(3)	0.007(2)	0.011(2)	-0.006(2)
C009	0.050(3)	0.056(3)	0.041(2)	0.0012(19)	0.014(2)	-0.001(2)
C00A	0.059(3)	0.067(3)	0.057(3)	0.012(2)	0.015(2)	-0.012(2)
C00B	0.083(4)	0.090(4)	0.040(3)	-0.004(2)	0.023(2)	-0.001(3)
C00C	0.089(4)	0.066(3)	0.051(3)	0.003(2)	0.029(3)	0.005(3)
C00D	0.074(4)	0.067(3)	0.059(3)	-0.004(2)	0.022(3)	0.009(3)
C00E	0.060(3)	0.060(3)	0.066(3)	0.000(2)	0.024(2)	0.002(2)
C00F	0.070(3)	0.058(3)	0.067(3)	0.004(2)	0.016(3)	-0.004(2)
C00G	0.054(3)	0.069(3)	0.070(3)	0.003(3)	0.010(2)	-0.005(2)
C00H	0.104(5)	0.060(3)	0.090(4)	-0.006(3)	0.027(4)	0.011(3)
C00I	0.117(5)	0.078(4)	0.066(4)	0.018(3)	0.041(3)	0.003(3)
C00J	0.125(5)	0.058(4)	0.088(4)	0.018(3)	0.024(4)	0.003(3)

Table 8. Hydrogen atomic coordinates and isotropic atomic displacement parameters (Å^2) for 211119MS114overweekend.

	x/a	y/b	z/c	U(eq)

	x/a	y/b	z/c	U(eq)
H005	0.7287	0.2611	0.5491	0.057
H00A	0.5718	0.2286	0.2532	0.061
H00B	0.7822	0.2630	0.3243	0.061
H00C	0.2210	0.2144	0.3423	0.063
H00D	0.2202	0.2437	0.4622	0.063
H00E	0.4934	0.1700	0.5458	0.072
H00F	0.2826	0.1337	0.4788	0.072
H00G	0.3675	0.3446	0.5583	0.104
H00H	0.5723	0.3372	0.6592	0.104
H00I	0.4119	0.2794	0.6215	0.104
H00J	0.6275	0.3581	0.1722	0.08
H00K	0.3763	0.3815	0.1454	0.08
H00L	0.2146	0.4327	0.3135	0.079
H00M	0.3815	0.4394	0.4296	0.079
H00N	0.8282	0.1515	0.3290	0.072
H00O	0.8463	0.1796	0.4512	0.072
H00P	0.6212	0.0906	0.4391	0.077
H00Q	0.4664	0.1158	0.3281	0.077
H00R	0.8509	0.3651	0.3430	0.078
H00S	0.7998	0.4278	0.4026	0.078
H00T	0.3361	0.5366	0.3290	0.1
H00U	0.5899	0.5153	0.3563	0.1
H00V	0.7555	0.4627	0.1983	0.101
H00W	0.6031	0.4562	0.0777	0.101
H00X	0.2995	0.5006	0.1434	0.108
H00Y	0.5053	0.5475	0.1679	0.108
