# Supplementary material for: Conformational tuning improves the stability of spirocyclic nitroxides with long paramagnetic relaxation times
Source: Commun Chem. 2023 Jun 5;6:111. doi: 10.1038/s42004-023-00912-7 (PMC10241799; doi:10.1038/s42004-023-00912-7)
Supplement: Supplementary file 8 — Supplementary Data 5 [file 42004_2023_912_MOESM8_ESM.pdf]

Crystal Structure Report for 211105MS140B

A clear intense orange rhombic-like specimen of $C_{2.19}H_{3.61}N_{0.13}O_{0.26}$, approximate dimensions 0.100 mm x 0.100 mm x 0.500 mm, was used for the X-ray crystallographic analysis. The X-ray intensity data were measured (λ = 1.54178 Å).

Table 1: Data collection details for 211105MS140B.

Axis	dx/mm	2θ/°	ω/°	φ/°	χ/°	Width/°	Frames	Time/s	Wavelength/Å	Voltage/kV	Current/mA	Temperature/K
Phi	37.022	91.28	91.67	0.00	-44.50	1.40	257	3.40	1.54184	50	1.1	302
Phi	37.022	106.28	14.21	-164.00	22.00	1.40	137	5.00	1.54184	50	1.1	302
Phi	37.022	50.04	317.96	-44.00	22.00	1.40	143	1.10	1.54184	50	1.1	302
Omega	37.022	106.28	-20.82	80.00	80.00	1.40	64	5.00	1.54184	50	1.1	302
Omega	37.022	50.04	-31.72	0.00	80.00	1.40	63	1.10	1.54184	50	1.1	302
Omega	37.022	106.28	-20.82	120.00	80.00	1.40	64	5.00	1.54184	50	1.1	302
Omega	37.022	106.28	-9.01	-40.00	65.50	1.40	69	5.00	1.54184	50	1.1	302
Omega	37.022	76.28	76.67	270.00	-44.50	1.40	56	2.20	1.54184	50	1.1	302
Phi	37.022	106.28	109.92	-112.25	-22.00	1.40	200	5.00	1.54184	50	1.1	302
Omega	37.022	-78.08	-77.94	48.00	-48.72	1.40	75	2.40	1.54184	50	1.1	302
Omega	37.022	106.28	-9.01	120.00	65.50	1.40	69	5.00	1.54184	50	1.1	302
Omega	37.022	106.28	-20.82	0.00	80.00	1.40	64	5.00	1.54184	50	1.1	302
Omega	37.022	106.28	-20.82	-160.00	80.00	1.40	64	5.00	1.54184	50	1.1	302
Phi	37.022	76.28	76.67	0.00	-44.50	1.40	257	2.20	1.54184	50	1.1	302
Omega	37.022	106.28	-9.01	-80.00	65.50	1.40	69	5.00	1.54184	50	1.1	302
Omega	37.022	50.04	-38.67	-54.00	65.50	1.40	67	1.10	1.54184	50	1.1	302
Omega	37.022	61.28	61.67	270.00	-44.50	1.40	67	1.50	1.54184	50	1.1	302
Omega	37.022	106.28	-20.82	40.00	80.00	1.40	64	5.00	1.54184	50	1.1	302
Omega	37.022	61.28	61.67	180.00	-44.50	1.40	67	1.50	1.54184	50	1.1	302
Omega	37.022	106.28	-20.82	160.00	80.00	1.40	64	5.00	1.54184	50	1.1	302
Omega	37.022	106.28	-20.82	-40.00	80.00	1.40	64	5.00	1.54184	50	1.1	302
Omega	37.022	76.28	76.67	180.00	-44.50	1.40	56	2.20	1.54184	50	1.1	302
Omega	37.022	106.28	-9.01	80.00	65.50	1.40	69	5.00	1.54184	50	1.1	302
Omega	37.022	50.04	-38.67	0.00	65.50	1.40	67	1.10	1.54184	50	1.1	302
Phi	37.022	106.28	106.67	-88.25	-44.50	1.40	166	5.00	1.54184	50	1.1	302

A total of 2402 frames were collected. The total exposure time was 2.38 hours. The frames were integrated with the Bruker SAINT software package using a narrow-frame algorithm. The integration of the data using a monoclinic unit cell yielded a total of 27477 reflections to a maximum θ angle of 68.58° (0.83 Å resolution), of which 2793 were independent (average redundancy 9.838, completeness = 99.1%, R_{int} = 23.19%, R_{sig} = 11.31%) and 1823 (65.27%) were greater than 2σ(F^2). The final cell constants of a = 19.000(4) Å, b = 6.0889(12) Å, c = 27.040(5) Å, β = 101.415(8)°, volume = 3066.4(10) $Å^3$, are based upon the refinement of the XYZ-centroids of 9716 reflections above 20 σ(I) with 10.47° < 2θ < 136.1°. Data were corrected for absorption effects using the Multi-Scan method (SADABS). The ratio of minimum to maximum apparent transmission was 0.503. The calculated minimum and maximum transmission coefficients (based on crystal size) are 0.7510 and 0.9420.

The structure was solved and refined using the Bruker SHELXTL Software Package, using the space group C 1 2/c 1, with Z = 62 for the formula unit, $C_{2.19}H_{3.61}N_{0.13}O_{0.26}$. The final anisotropic full-matrix least-squares refinement on F^2 with 186 variables converged at R1 = 7.03%, for the observed data and wR2 = 23.33% for all data. The goodness-of-fit was 1.034. The largest peak in the final difference electron density synthesis was 0.621 $e^-/Å^3$ and the largest hole was -0.611 $e^-/Å^3$ with an RMS deviation of 0.204 $e^-/Å^3$. On the basis of the final model, the calculated density was 1.206 g/cm^3 and F(000), 1224 e^-.

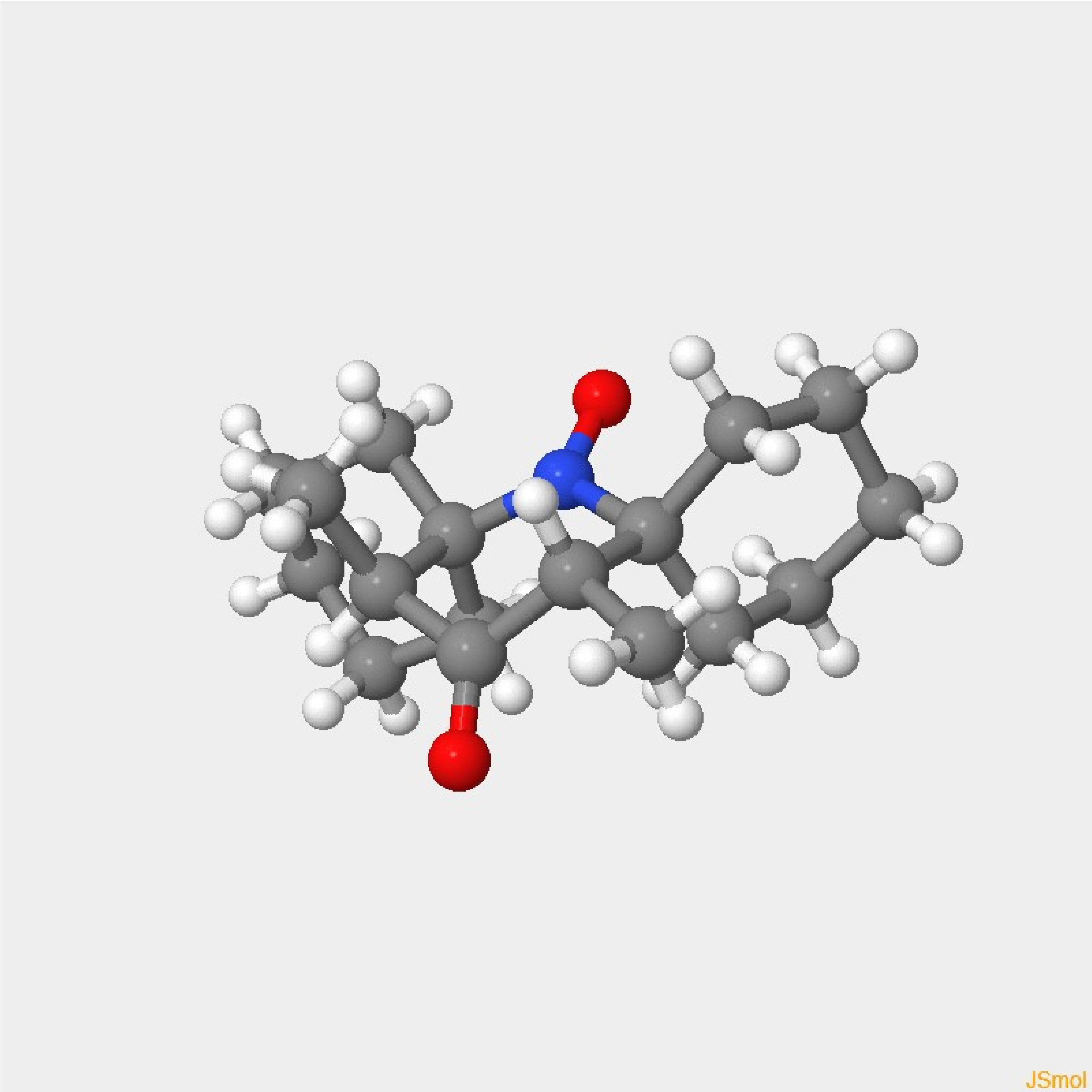
JSmol

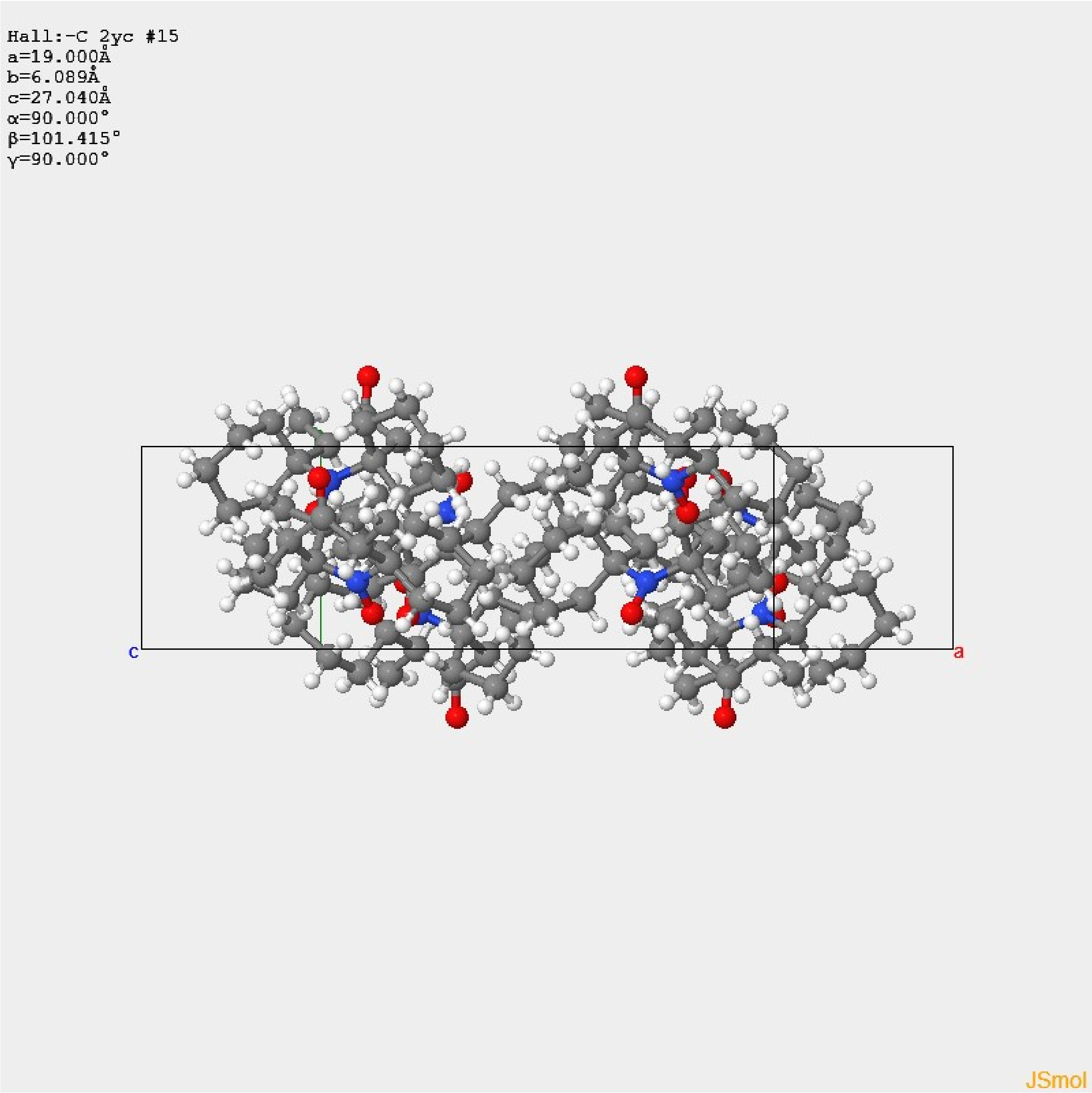

Table 2. Sample and crystal data for 211105MS140B.

**Identification code**	211105MS140B	
**Chemical formula**	$C_{2.19}H_{3.61}N_{0.13}O_{0.26}$	
**Formula weight**	35.92 g/mol	
**Temperature**	302(2) K	
**Wavelength**	1.54178 Å	
**Crystal size**	0.100 x 0.100 x 0.500 mm	
**Crystal habit**	clear intense orange rhombic	
**Crystal system**	monoclinic	
**Space group**	C 1 2/c 1	
**Unit cell dimensions**	a = 19.000(4) Å	α = 90°
	b = 6.0889(12) Å	β = 101.415(8)°
	c = 27.040(5) Å	γ = 90°
**Volume**	3066.4(10) $Å^3$	
**Z**	62	
**Density (calculated)**	1.206 g/cm^3	

**Absorption coefficient**	0.607 mm^{-1}
**F(000)**	1224

Table 3. Data collection and structure refinement for 211105MS140B.

**Theta range for data collection**	3.33 to 68.58°	
**Index ranges**	-22<=h<=22, -7<=k<=7, -32<=l<=32	
**Reflections collected**	27477	
**Independent reflections**	2793 [R(int) = 0.2319]	
**Coverage of independent reflections**	99.1%	
**Absorption correction**	Multi-Scan	
**Max. and min. transmission**	0.9420 and 0.7510	
**Structure solution technique**	direct methods	
**Structure solution program**	SHELXT 2014/5 (Sheldrick, 2014)	
**Refinement method**	Full-matrix least-squares on F^2	
**Refinement program**	SHELXL-2018/3 (Sheldrick, 2018)	
**Function minimized**	$\Sigma w(F_o^2 - F_c^2)^2$	
**Data / restraints / parameters**	2793 / 0 / 186	
**Goodness-of-fit on F^2**	1.034	
**Final R indices**	1823 data; I>2σ(I)	R1 = 0.0703, wR2 = 0.1963
	all data	R1 = 0.1386, wR2 = 0.2333
**Weighting scheme**	$w=1/[\sigma^2(F_o^2)+(0.1562P)^2]$<br>where $P=(F_o^2+2F_c^2)/3$	
**Largest diff. peak and hole**	0.621 and -0.611 eÅ^{-3}	
**R.M.S. deviation from mean**	0.204 eÅ^{-3}	

Table 4. Atomic coordinates and equivalent isotropic atomic displacement parameters (Å^2) for 211105MS140B.

U(eq) is defined as one third of the trace of the orthogonalized U_{ij} tensor.

	x/a	y/b	z/c	U(eq)
O001	0.26509(12)	0.1620(4)	0.44273(6)	0.0596(6)
N1	0.30424(10)	0.6726(3)	0.36841(6)	0.0344(5)
O002	0.32000(11)	0.8285(4)	0.34106(7)	0.0635(6)
C004	0.18084(12)	0.7517(5)	0.32639(8)	0.0426(6)
C005	0.23094(11)	0.5694(4)	0.35169(7)	0.0326(5)
C006	0.42089(13)	0.7583(5)	0.42369(9)	0.0467(7)
C007	0.23525(12)	0.3871(4)	0.31272(7)	0.0386(6)
C008	0.36516(11)	0.5763(4)	0.40600(7)	0.0362(6)
C009	0.44126(14)	0.4606(6)	0.34267(8)	0.0511(7)
C00A	0.20624(11)	0.4722(5)	0.39900(7)	0.0381(6)
C00B	0.16144(13)	0.3084(5)	0.28440(8)	0.0461(7)
C00C	0.17503(15)	0.6413(5)	0.43084(9)	0.0515(7)
C00D	0.26844(13)	0.3536(5)	0.43217(7)	0.0411(6)
C00E	0.40159(12)	0.3864(5)	0.38342(8)	0.0408(6)
C00F	0.47017(15)	0.8193(6)	0.38738(11)	0.0585(8)
C00G	0.33301(12)	0.4970(5)	0.45193(7)	0.0426(7)
C00H	0.50026(14)	0.6227(6)	0.36461(10)	0.0605(9)
C00I	0.11407(14)	0.4993(6)	0.26090(9)	0.0541(8)
C00J	0.10656(13)	0.6661(5)	0.30075(9)	0.0500(7)
C00K	0.38564(15)	0.3801(6)	0.49424(8)	0.0611(9)

Table 5. Bond lengths (Å) for 211105MS140B.

O001-C00D	1.206(3)	N1-O002	1.274(3)
N1-C008	1.501(3)	N1-C005	1.513(3)
C004-C005	1.532(3)	C004-C00J	1.535(3)
C004-H00A	0.97	C004-H00B	0.97
C005-C007	1.544(3)	C005-C00A	1.564(3)
C006-C00F	1.531(4)	C006-C008	1.542(3)
C006-H00C	0.97	C006-H00D	0.97
C007-C00B	1.536(3)	C007-H00E	0.97
C007-H00F	0.97	C008-C00E	1.535(3)
C008-C00G	1.565(3)	C009-C00E	1.522(3)
C009-C00H	1.523(4)	C009-H00G	0.97
C009-H00H	0.97	C00A-C00D	1.518(3)
C00A-C00C	1.534(3)	C00A-H00I	0.98
C00B-C00I	1.529(4)	C00B-H00J	0.97
C00B-H00K	0.97	C00C-H00L	0.96
C00C-H00M	0.96	C00C-H00N	0.96
C00D-C00G	1.514(3)	C00E-H00O	0.97
C00E-H00P	0.97	C00F-C00H	1.509(5)
C00F-H00Q	0.97	C00F-H00R	0.97
C00G-C00K	1.537(3)	C00G-H00S	0.98
C00H-H00T	0.97	C00H-H00U	0.97
C00I-C00J	1.508(4)	C00I-H00V	0.97
C00I-H00W	0.97	C00J-H00X	0.97
C00J-H00Y	0.97	C00K-H00Z	0.96
C00K-H010	0.96	C00K-H011	0.96

Table 6. Bond angles (°) for 211105MS140B.

O002-N1-C008	116.47(18)	O002-N1-C005	116.35(16)
C008-N1-C005	125.52(18)	C005-C004-C00J	113.0(2)
C005-C004-H00A	109.0	C00J-C004-H00A	109.0
C005-C004-H00B	109.0	C00J-C004-H00B	109.0
H00A-C004-H00B	107.8	N1-C005-C004	106.63(19)
N1-C005-C007	109.43(17)	C004-C005-C007	109.17(16)
N1-C005-C00A	108.68(14)	C004-C005-C00A	112.26(18)
C007-C005-C00A	110.6(2)	C00F-C006-C008	116.4(2)
C00F-C006-H00C	108.2	C008-C006-H00C	108.2
C00F-C006-H00D	108.2	C008-C006-H00D	108.2
H00C-C006-H00D	107.3	C00B-C007-C005	113.47(19)
C00B-C007-H00E	108.9	C005-C007-H00E	108.9
C00B-C007-H00F	108.9	C005-C007-H00F	108.9
H00E-C007-H00F	107.7	N1-C008-C00E	111.91(15)
N1-C008-C006	108.6(2)	C00E-C008-C006	109.2(2)
N1-C008-C00G	106.76(16)	C00E-C008-C00G	111.1(2)
C006-C008-C00G	109.12(17)	C00E-C009-C00H	110.0(2)
C00E-C009-H00G	109.7	C00H-C009-H00G	109.7
C00E-C009-H00H	109.7	C00H-C009-H00H	109.7
H00G-C009-H00H	108.2	C00D-C00A-C00C	109.26(17)
C00D-C00A-C005	109.82(17)	C00C-C00A-C005	114.7(2)
C00D-C00A-H00I	107.6	C00C-C00A-H00I	107.6
C005-C00A-H00I	107.6	C00I-C00B-C007	112.0(2)
C00I-C00B-H00J	109.2	C007-C00B-H00J	109.2
C00I-C00B-H00K	109.2	C007-C00B-H00K	109.2
H00J-C00B-H00K	107.9	C00A-C00C-H00L	109.5
C00A-C00C-H00M	109.5	H00L-C00C-H00M	109.5

C00A-C00C-H00N	109.5	H00L-C00C-H00N	109.5
H00M-C00C-H00N	109.5	O001-C00D-C00G	123.4(2)
O001-C00D-C00A	121.9(2)	C00G-C00D-C00A	114.6(2)
C009-C00E-C008	113.0(2)	C009-C00E-H00O	109.0
C008-C00E-H00O	109.0	C009-C00E-H00P	109.0
C008-C00E-H00P	109.0	H00O-C00E-H00P	107.8
C00H-C00F-C006	113.5(3)	C00H-C00F-H00Q	108.9
C006-C00F-H00Q	108.9	C00H-C00F-H00R	108.9
C006-C00F-H00R	108.9	H00Q-C00F-H00R	107.7
C00D-C00G-C00K	111.0(2)	C00D-C00G-C008	108.31(16)
C00K-C00G-C008	115.8(2)	C00D-C00G-H00S	107.1
C00K-C00G-H00S	107.1	C008-C00G-H00S	107.1
C00F-C00H-C009	111.3(2)	C00F-C00H-H00T	109.4
C009-C00H-H00T	109.4	C00F-C00H-H00U	109.4
C009-C00H-H00U	109.4	H00T-C00H-H00U	108.0
C00J-C00I-C00B	110.26(19)	C00J-C00I-H00V	109.6
C00B-C00I-H00V	109.6	C00J-C00I-H00W	109.6
C00B-C00I-H00W	109.6	H00V-C00I-H00W	108.1
C00I-C00J-C004	110.0(2)	C00I-C00J-H00X	109.7
C004-C00J-H00X	109.7	C00I-C00J-H00Y	109.7
C004-C00J-H00Y	109.7	H00X-C00J-H00Y	108.2
C00G-C00K-H00Z	109.5	C00G-C00K-H010	109.5
H00Z-C00K-H010	109.5	C00G-C00K-H011	109.5
H00Z-C00K-H011	109.5	H010-C00K-H011	109.5

Table 7. Anisotropic atomic displacement parameters (Å^2) for 211105MS140B.

The anisotropic atomic displacement factor exponent takes the form: $-2\pi^2[h^2 a^{*2} U_{11} + ... + 2 h k a^* b^* U_{12}]$

	U_{11}	U_{22}	U_{33}	U_{23}	U_{13}	U_{12}
O001	0.0702(13)	0.0485(15)	0.0603(10)	0.0146(9)	0.0133(9)	-0.0043(10)
N1	0.0334(10)	0.0352(13)	0.0361(8)	0.0050(7)	0.0102(7)	-0.0013(8)
O002	0.0488(10)	0.0658(15)	0.0736(11)	0.0366(10)	0.0063(9)	-0.0096(10)
C004	0.0408(12)	0.0402(17)	0.0472(11)	0.0038(10)	0.0100(9)	0.0060(12)
C005	0.0322(10)	0.0336(14)	0.0325(9)	0.0014(8)	0.0075(7)	0.0022(10)
C006	0.0432(13)	0.0443(17)	0.0495(11)	-0.0024(11)	0.0020(10)	-0.0047(12)
C007	0.0372(11)	0.0444(16)	0.0348(9)	-0.0033(9)	0.0085(8)	0.0037(11)
C008	0.0351(10)	0.0387(15)	0.0343(9)	0.0018(9)	0.0053(8)	0.0011(10)
C009	0.0448(12)	0.060(2)	0.0506(12)	-0.0004(12)	0.0154(10)	0.0099(13)
C00A	0.0351(10)	0.0457(16)	0.0352(9)	0.0005(9)	0.0111(8)	-0.0039(11)
C00B	0.0431(13)	0.0515(18)	0.0419(10)	-0.0103(10)	0.0038(10)	-0.0013(12)
C00C	0.0545(14)	0.059(2)	0.0471(11)	-0.0038(11)	0.0245(11)	0.0046(13)
C00D	0.0467(13)	0.0474(18)	0.0324(9)	0.0034(9)	0.0153(9)	-0.0004(12)
C00E	0.0353(11)	0.0437(17)	0.0422(10)	0.0019(10)	0.0048(9)	0.0048(11)
C00F	0.0415(13)	0.061(2)	0.0718(15)	0.0071(14)	0.0086(12)	-0.0139(13)
C00G	0.0447(12)	0.0510(18)	0.0326(9)	0.0012(10)	0.0086(9)	-0.0007(12)
C00H	0.0396(13)	0.074(2)	0.0717(16)	0.0122(15)	0.0203(12)	0.0007(14)
C00I	0.0414(12)	0.071(2)	0.0450(11)	-0.0001(12)	-0.0031(10)	0.0018(13)
C00J	0.0350(12)	0.059(2)	0.0542(13)	0.0073(12)	0.0048(10)	0.0118(12)
C00K	0.0529(14)	0.087(3)	0.0406(11)	0.0169(13)	0.0018(11)	-0.0010(16)

Table 8. Hydrogen atomic coordinates and isotropic atomic displacement parameters (Å^2) for 211105MS140B.

	x/a	y/b	z/c	U(eq)

	x/a	y/b	z/c	U(eq)
H00A	0.2029	0.8244	0.3014	0.051
H00B	0.1750	0.8599	0.3516	0.051
H00C	0.3954	0.8894	0.4304	0.056
H00D	0.4507	0.7123	0.4554	0.056
H00E	0.2623	0.4415	0.2884	0.046
H00F	0.2612	0.2629	0.3299	0.046
H00G	0.4078	0.5296	0.3153	0.061
H00H	0.4622	0.3342	0.3291	0.061
H00I	0.1688	0.3633	0.3871	0.046
H00J	0.1679	0.2068	0.2580	0.055
H00K	0.1375	0.2302	0.3076	0.055
H00L	0.2062	0.7667	0.4367	0.077
H00M	0.1710	0.5765	0.4625	0.077
H00N	0.1284	0.6863	0.4132	0.077
H00O	0.4353	0.3147	0.4102	0.049
H00P	0.3654	0.2792	0.3692	0.049
H00Q	0.4435	0.9089	0.3604	0.07
H00R	0.5097	0.9071	0.4054	0.07
H00S	0.3154	0.6276	0.4668	0.051
H00T	0.5353	0.5500	0.3903	0.073
H00U	0.5245	0.6714	0.3382	0.073
H00V	0.1352	0.5684	0.2350	0.065
H00W	0.0670	0.4444	0.2452	0.065
H00X	0.0831	0.5992	0.3257	0.06
H00Y	0.0771	0.7876	0.2854	0.06
H00Z	0.4015	0.2455	0.4816	0.092
H010	0.3620	0.3486	0.5217	0.092
H011	0.4263	0.4733	0.5059	0.092
