# Supplementary material for: Conformational tuning improves the stability of spirocyclic nitroxides with long paramagnetic relaxation times
Source: Commun Chem. 2023 Jun 5;6:111. doi: 10.1038/s42004-023-00912-7 (PMC10241799; doi:10.1038/s42004-023-00912-7)
Supplement: Supplementary file 10 — Supplementary Data 7 [file 42004_2023_912_MOESM10_ESM.pdf]

Crystal Structure Report for 211108MS142

A clear intense orange box-like specimen of $C_{2.12}H_{3.62}N_{0.12}O_{0.25}$, approximate dimensions 0.200 mm x 0.200 mm x 0.400 mm, was used for the X-ray crystallographic analysis. The X-ray intensity data were measured (λ = 1.54178 Å).

Table 1: Data collection details for 211108MS142.

Axis	dx/mm	2θ/°	ω/°	φ/°	χ/°	Width/°	Frames	Time/s	Wavelength/Å	Voltage/kV	Current/mA	Temperature/K
Omega	37.039	106.28	-9.01	-120.00	65.50	2.00	48	5.00	1.54184	50	1.1	302
Phi	37.039	91.28	91.67	0.00	-44.50	2.00	180	3.70	1.54184	50	1.1	302
Omega	37.039	20.04	20.74	90.00	-44.50	2.00	51	1.50	1.54184	50	1.1	302
Phi	37.039	106.28	13.26	-190.60	24.00	2.00	151	5.00	1.54184	50	1.1	302
Omega	37.039	61.28	61.67	270.00	-44.50	2.00	47	2.10	1.54184	50	1.1	302
Omega	37.039	106.28	-19.22	-80.00	80.00	2.00	44	5.00	1.54184	50	1.1	302
Omega	37.039	106.28	-9.01	80.00	65.50	2.00	48	5.00	1.54184	50	1.1	302
Omega	37.039	61.28	61.67	180.00	-44.50	2.00	47	2.10	1.54184	50	1.1	302
Phi	37.039	106.28	110.24	-60.00	-24.00	2.00	120	5.00	1.54184	50	1.1	302
Omega	37.039	50.04	-38.87	-105.00	65.50	2.00	47	1.80	1.54184	50	1.1	302
Omega	37.039	106.28	-9.01	-160.00	65.50	2.00	48	5.00	1.54184	50	1.1	302
Omega	37.039	106.28	-19.22	-40.00	80.00	2.00	44	5.00	1.54184	50	1.1	302
Omega	37.039	76.28	76.67	90.00	-44.50	2.00	39	2.80	1.54184	50	1.1	302
Omega	37.039	76.28	76.67	0.00	-44.50	2.00	39	2.80	1.54184	50	1.1	302
Omega	37.039	106.28	-19.22	-120.00	80.00	2.00	44	5.00	1.54184	50	1.1	302
Omega	37.039	35.04	35.74	90.00	-44.50	2.00	51	1.60	1.54184	50	1.1	302
Omega	37.039	106.28	-19.22	40.00	80.00	2.00	44	5.00	1.54184	50	1.1	302
Omega	37.039	50.04	-38.87	102.00	65.50	2.00	47	1.80	1.54184	50	1.1	302
Omega	37.039	106.28	-19.22	160.00	80.00	2.00	44	5.00	1.54184	50	1.1	302
Phi	37.039	106.28	106.67	0.00	-44.50	2.00	180	5.00	1.54184	50	1.1	302
Omega	37.039	106.28	-9.01	-40.00	65.50	2.00	48	5.00	1.54184	50	1.1	302
Omega	37.039	61.28	61.67	90.00	-44.50	2.00	47	2.10	1.54184	50	1.1	302

A total of 1458 frames were collected. The total exposure time was 1.62 hours. The frames were integrated with the Bruker SAINT software package using a narrow-frame algorithm. The integration of the data using a monoclinic unit cell yielded a total of 34904 reflections to a maximum θ angle of 70.11° (0.82 Å resolution), of which 2996 were independent (average redundancy 11.650, completeness = 99.5%, R_{int} = 5.92%, R_{sig} = 2.80%) and 2597 (86.68%) were greater than 2σ(F^2). The final cell constants of a = 9.3266 (5) Å, b = 13.0922(7) Å, c = 13.6536(7) Å, β = 108.023(2)°, volume = 1585.38(15) $Å^3$, are based upon the refinement of the XYZ-centroids of 9921 reflections above 20 σ(I) with 9.593° < 2θ < 140.2°. Data were corrected for absorption effects using the Multi-Scan method (SADABS). The ratio of minimum to maximum apparent transmission was 0.738. The calculated minimum and maximum transmission coefficients (based on crystal size) are 0.7990 and 0.8920.

The structure was solved and refined using the Bruker SHELXTL Software Package, using the space group P 1 21/n 1, with Z = 32 for the formula unit, $C_{2.12}H_{3.62}N_{0.12}O_{0.25}$. The final anisotropic full-matrix least-squares refinement on F^2 with 183 variables converged at R1 = 4.96%, for the observed data and wR2 = 14.20% for all data. The goodness-of-fit was 1.048. The largest peak in the final difference electron density synthesis was 0.491 $e^-/Å^3$ and the largest hole was -0.201 $e^-/Å^3$ with an RMS deviation of 0.036 $e^-/Å^3$. On the basis of the final model, the calculated density was 1.171 g/cm^3 and F(000), 616 e^-.

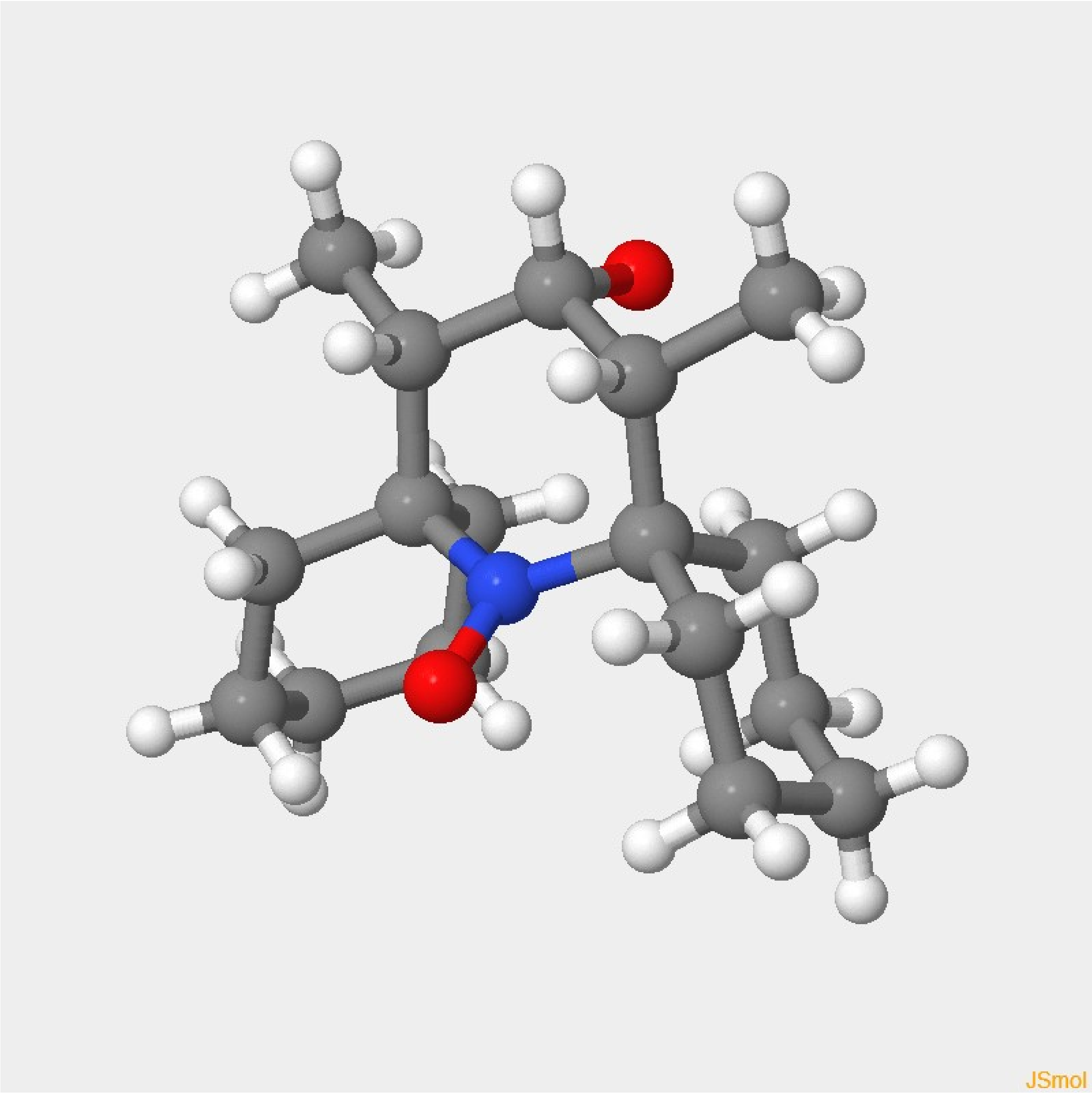
JSmol

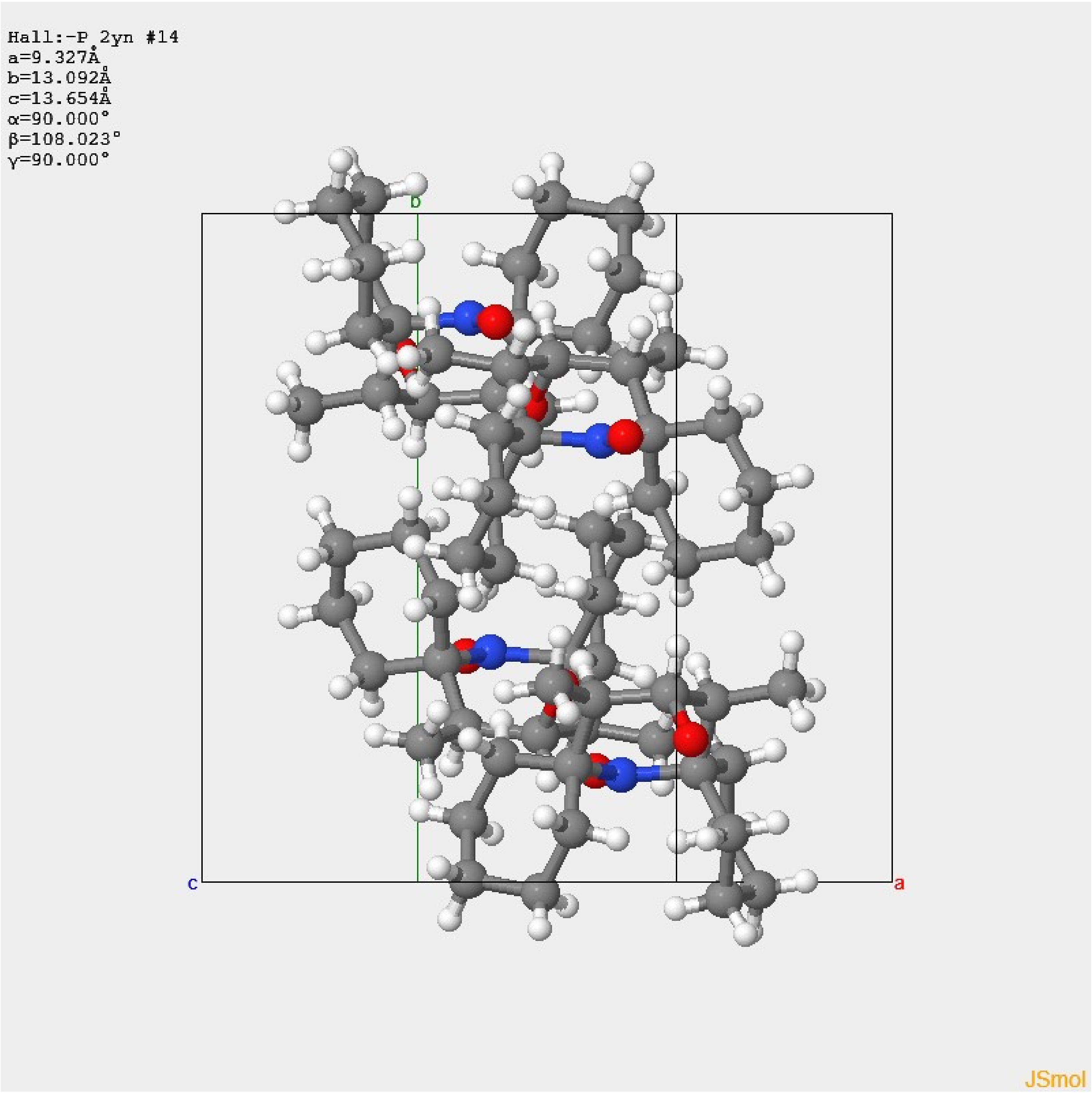

Table 2. Sample and crystal data for 211108MS142.

**Identification code**	211108MS142	
**Chemical formula**	$C_{2.12}H_{3.62}N_{0.12}O_{0.25}$	
**Formula weight**	34.93 g/mol	
**Temperature**	302(2) K	
**Wavelength**	1.54178 Å	
**Crystal size**	0.200 x 0.200 x 0.400 mm	
**Crystal habit**	clear intense orange box	
**Crystal system**	monoclinic	
**Space group**	P 1 21/n 1	
**Unit cell dimensions**	a = 9.3266(5) Å	α = 90°
	b = 13.0922(7) Å	β = 108.023(2)°
	c = 13.6536(7) Å	γ = 90°
**Volume**	1585.38(15) $Å^3$	
**Z**	32	
**Density (calculated)**	1.171 g/cm^3	

**Absorption coefficient**	0.587 mm^{-1}
**F(000)**	616

Table 3. Data collection and structure refinement for 211108MS142.

**Theta range for data collection**	4.80 to 70.11°	
**Index ranges**	-11<=h<=11, -15<=k<=15, -16<=l<=16	
**Reflections collected**	34904	
**Independent reflections**	2996 [R(int) = 0.0592]	
**Coverage of independent reflections**	99.5%	
**Absorption correction**	Multi-Scan	
**Max. and min. transmission**	0.8920 and 0.7990	
**Structure solution technique**	direct methods	
**Structure solution program**	SHELXT 2014/5 (Sheldrick, 2014)	
**Refinement method**	Full-matrix least-squares on F^2	
**Refinement program**	SHELXL-2018/3 (Sheldrick, 2018)	
**Function minimized**	Σ w(F_o^2 - F_c^2)^2	
**Data / restraints / parameters**	2996 / 0 / 183	
**Goodness-of-fit on F^2**	1.048	
**Final R indices**	2597 data; I>2σ(I)	R1 = 0.0496, wR2 = 0.1344
	all data	R1 = 0.0561, wR2 = 0.1420
**Weighting scheme**	w=1/[σ^2(F_o^2)+(0.0670P)^2+0.5736P]<br>where P=(F_o^2+2F_c^2)/3	
**Largest diff. peak and hole**	0.491 and -0.201 eÅ^{-3}	
**R.M.S. deviation from mean**	0.036 eÅ^{-3}	

Table 4. Atomic coordinates and equivalent isotropic atomic displacement parameters (Å^2) for 211108MS142.

U(eq) is defined as one third of the trace of the orthogonalized U_{ij} tensor.

	x/a	y/b	z/c	U(eq)
O001	0.54120(15)	0.28962(10)	0.51882(9)	0.0605(4)
N1	0.28271(13)	0.34207(9)	0.27547(8)	0.0320(3)
O003	0.18943(14)	0.33589(14)	0.18568(9)	0.0697(5)
C004	0.53890(17)	0.42711(12)	0.33808(11)	0.0399(4)
C005	0.25003(18)	0.42280(12)	0.43592(11)	0.0430(4)
C006	0.4992(2)	0.52543(12)	0.27576(13)	0.0479(4)
C007	0.21998(16)	0.32877(11)	0.36454(10)	0.0357(3)
C008	0.44834(16)	0.33316(11)	0.28458(11)	0.0342(3)
C009	0.50451(18)	0.23166(12)	0.34297(12)	0.0437(4)
C00A	0.29001(19)	0.22867(12)	0.42057(11)	0.0440(4)
C00B	0.45979(19)	0.21916(12)	0.44090(12)	0.0465(4)
C00C	0.4289(2)	0.42520(14)	0.11281(12)	0.0514(4)
C00D	0.1627(2)	0.51768(14)	0.38724(14)	0.0529(4)
C00E	0.46168(19)	0.32646(13)	0.17514(12)	0.0440(4)
C00F	0.04811(18)	0.31470(13)	0.31979(13)	0.0461(4)
C00G	0.5222(2)	0.51374(14)	0.17100(14)	0.0525(4)
C00H	0.95867(19)	0.41004(15)	0.27446(13)	0.0533(4)
C00I	0.9943(2)	0.49797(16)	0.35086(15)	0.0588(5)
C00J	0.6734(2)	0.21107(16)	0.36661(18)	0.0662(6)
C00K	0.2511(3)	0.20654(17)	0.51971(15)	0.0674(6)

Table 5. Bond lengths (Å) for 211108MS142.

O001-C00B	1.435(2)	N1-O003	1.2674(16)
N1-C007	1.5139(17)	N1-C008	1.5153(18)
C004-C006	1.524(2)	C004-C008	1.5421(19)
C004-H00A	0.97	C004-H00B	0.97
C005-C00D	1.521(2)	C005-C007	1.541(2)
C005-H00C	0.97	C005-H00D	0.97
C006-C00G	1.518(2)	C006-H00E	0.97
C006-H00F	0.97	C007-C00F	1.540(2)
C007-C00A	1.556(2)	C008-C00E	1.540(2)
C008-C009	1.555(2)	C009-C00B	1.528(2)
C009-C00J	1.531(2)	C009-H009	0.98
C00A-C00B	1.526(2)	C00A-C00K	1.533(2)
C00A-H00G	0.98	C00B-H00H	0.98
C00C-C00G	1.518(3)	C00C-C00E	1.526(2)
C00C-H00I	0.97	C00C-H00J	0.97
C00D-C00I	1.516(3)	C00D-H00K	0.97
C00D-H00L	0.97	C00E-H00M	0.97
C00E-H00N	0.97	C00F-C00H	1.522(3)
C00F-H00O	0.97	C00F-H00P	0.97
C00G-H00Q	0.97	C00G-H00R	0.97
C00H-C00I	1.520(3)	C00H-H00S	0.97
C00H-H00T	0.97	C00I-H00U	0.97
C00I-H00V	0.97	C00J-H00W	0.96
C00J-H00X	0.96	C00J-H00Y	0.96
C00K-H00Z	0.96	C00K-H010	0.96
C00K-H011	0.96		

Table 6. Bond angles (°) for 211108MS142.

O003-N1-C007	116.70(11)	O003-N1-C008	116.85(11)
C007-N1-C008	124.25(10)	C006-C004-C008	113.98(12)
C006-C004-H00A	108.8	C008-C004-H00A	108.8
C006-C004-H00B	108.8	C008-C004-H00B	108.8
H00A-C004-H00B	107.7	C00D-C005-C007	114.08(12)
C00D-C005-H00C	108.7	C007-C005-H00C	108.7
C00D-C005-H00D	108.7	C007-C005-H00D	108.7
H00C-C005-H00D	107.6	C00G-C006-C004	111.12(14)
C00G-C006-H00E	109.4	C004-C006-H00E	109.4
C00G-C006-H00F	109.4	C004-C006-H00F	109.4
H00E-C006-H00F	108.0	N1-C007-C00F	107.98(11)
N1-C007-C005	112.10(11)	C00F-C007-C005	108.05(13)
N1-C007-C00A	106.51(12)	C00F-C007-C00A	108.75(12)
C005-C007-C00A	113.29(12)	N1-C008-C00E	108.13(11)
N1-C008-C004	111.87(11)	C00E-C008-C004	107.95(12)
N1-C008-C009	106.32(11)	C00E-C008-C009	109.47(12)
C004-C008-C009	112.99(12)	C00B-C009-C00J	109.78(14)
C00B-C009-C008	113.60(13)	C00J-C009-C008	114.84(14)
C00B-C009-H009	106.0	C00J-C009-H009	106.0
C008-C009-H009	106.0	C00B-C00A-C00K	109.85(14)
C00B-C00A-C007	114.35(12)	C00K-C00A-C007	114.54(15)
C00B-C00A-H00G	105.7	C00K-C00A-H00G	105.7
C007-C00A-H00G	105.7	O001-C00B-C00A	111.33(14)
O001-C00B-C009	110.79(14)	C00A-C00B-C009	112.40(12)
O001-C00B-H00H	107.3	C00A-C00B-H00H	107.3
C009-C00B-H00H	107.3	C00G-C00C-C00E	111.80(13)

C00G-C00C-H00I	109.3	C00E-C00C-H00I	109.3
C00G-C00C-H00J	109.3	C00E-C00C-H00J	109.3
H00I-C00C-H00J	107.9	C00I-C00D-C005	111.39(15)
C00I-C00D-H00K	109.3	C005-C00D-H00K	109.3
C00I-C00D-H00L	109.3	C005-C00D-H00L	109.3
H00K-C00D-H00L	108.0	C00C-C00E-C008	115.50(13)
C00C-C00E-H00M	108.4	C008-C00E-H00M	108.4
C00C-C00E-H00N	108.4	C008-C00E-H00N	108.4
H00M-C00E-H00N	107.5	C00H-C00F-C007	115.81(13)
C00H-C00F-H00O	108.3	C007-C00F-H00O	108.3
C00H-C00F-H00P	108.3	C007-C00F-H00P	108.3
H00O-C00F-H00P	107.4	C006-C00G-C00C	110.17(14)
C006-C00G-H00Q	109.6	C00C-C00G-H00Q	109.6
C006-C00G-H00R	109.6	C00C-C00G-H00R	109.6
H00Q-C00G-H00R	108.1	C00I-C00H-C00F	111.49(14)
C00I-C00H-H00S	109.3	C00F-C00H-H00S	109.3
C00I-C00H-H00T	109.3	C00F-C00H-H00T	109.3
H00S-C00H-H00T	108.0	C00D-C00I-C00H	109.75(14)
C00D-C00I-H00U	109.7	C00H-C00I-H00U	109.7
C00D-C00I-H00V	109.7	C00H-C00I-H00V	109.7
H00U-C00I-H00V	108.2	C009-C00J-H00W	109.5
C009-C00J-H00X	109.5	H00W-C00J-H00X	109.5
C009-C00J-H00Y	109.5	H00W-C00J-H00Y	109.5
H00X-C00J-H00Y	109.5	C00A-C00K-H00Z	109.5
C00A-C00K-H010	109.5	H00Z-C00K-H010	109.5
C00A-C00K-H011	109.5	H00Z-C00K-H011	109.5
H010-C00K-H011	109.5		

Table 7. Anisotropic atomic displacement parameters ($Å^2$) for 211108MS142.

The anisotropic atomic displacement factor exponent takes the form: $-2\pi^2[h^2 a^{*2} U_{11} + ... + 2 h k a^* b^* U_{12}]$

	U_{11}	U_{22}	U_{33}	U_{23}	U_{13}	U_{12}
O001	0.0664(8)	0.0583(8)	0.0385(6)	0.0026(5)	-0.0105(6)	-0.0090(6)
N1	0.0340(6)	0.0360(6)	0.0215(6)	-0.0028(4)	0.0021(4)	-0.0019(5)
O003	0.0423(7)	0.1347(14)	0.0248(6)	-0.0117(6)	-0.0002(5)	-0.0066(7)
C004	0.0389(8)	0.0410(8)	0.0360(8)	-0.0021(6)	0.0058(6)	-0.0065(6)
C005	0.0477(9)	0.0501(9)	0.0293(7)	-0.0093(6)	0.0090(6)	-0.0038(7)
C006	0.0551(10)	0.0388(8)	0.0487(9)	0.0005(7)	0.0143(7)	-0.0048(7)
C007	0.0387(8)	0.0408(8)	0.0256(7)	-0.0013(5)	0.0068(6)	-0.0055(6)
C008	0.0347(7)	0.0354(7)	0.0295(7)	-0.0011(5)	0.0054(5)	0.0005(5)
C009	0.0437(8)	0.0354(8)	0.0456(9)	0.0003(6)	0.0042(7)	0.0036(6)
C00A	0.0546(9)	0.0400(8)	0.0330(7)	0.0041(6)	0.0073(7)	-0.0090(7)
C00B	0.0532(9)	0.0366(8)	0.0394(8)	0.0067(6)	-0.0008(7)	-0.0016(7)
C00C	0.0567(10)	0.0659(11)	0.0345(8)	0.0053(7)	0.0183(7)	0.0052(8)
C00D	0.0614(11)	0.0483(9)	0.0486(9)	-0.0101(7)	0.0165(8)	0.0014(8)
C00E	0.0459(9)	0.0501(9)	0.0377(8)	-0.0057(7)	0.0152(7)	0.0035(7)
C00F	0.0401(8)	0.0568(10)	0.0406(8)	-0.0033(7)	0.0112(7)	-0.0105(7)
C00G	0.0543(10)	0.0527(10)	0.0543(10)	0.0144(8)	0.0224(8)	0.0033(8)
C00H	0.0389(8)	0.0732(12)	0.0449(9)	-0.0041(8)	0.0089(7)	0.0036(8)
C00I	0.0573(11)	0.0674(12)	0.0530(10)	-0.0048(9)	0.0189(8)	0.0142(9)
C00J	0.0519(11)	0.0610(12)	0.0790(14)	0.0139(10)	0.0102(9)	0.0177(9)
C00K	0.0825(14)	0.0742(13)	0.0451(10)	0.0193(9)	0.0190(9)	-0.0102(11)

Table 8. Hydrogen atomic coordinates and isotropic atomic displacement parameters (Å^2) for 211108MS142.

	x/a	y/b	z/c	U(eq)
H00A	0.5214	0.4371	0.4038	0.048
H00B	0.6455	0.4133	0.3518	0.048
H00C	0.2240	0.4059	0.4975	0.052
H00D	0.3570	0.4382	0.4568	0.052
H00E	0.3949	0.5430	0.2667	0.058
H00F	0.5621	0.5806	0.3131	0.058
H009	0.4529	0.1768	0.2966	0.052
H00G	0.2446	0.1730	0.3731	0.053
H00H	0.4891	0.1501	0.4672	0.056
H00I	0.4506	0.4153	0.0484	0.062
H00J	0.3226	0.4416	0.0966	0.062
H00K	0.1849	0.5728	0.4372	0.063
H00L	0.1948	0.5388	0.3292	0.063
H00M	0.5631	0.3044	0.1803	0.053
H00N	0.3928	0.2742	0.1374	0.053
H00O	0.0283	0.2629	0.2664	0.055
H00P	0.0108	0.2889	0.3739	0.055
H00Q	0.6280	0.5016	0.1797	0.063
H00R	0.4926	0.5762	0.1318	0.063
H00S	-0.0176	0.4301	0.2128	0.064
H00T	-0.1483	0.3949	0.2550	0.064
H00U	-0.0585	0.5589	0.3184	0.071
H00V	-0.0397	0.4813	0.4093	0.071
H00W	0.7003	0.2165	0.3043	0.099
H00X	0.6963	0.1436	0.3946	0.099
H00Y	0.7297	0.2603	0.4157	0.099
H00Z	0.1455	0.2180	0.5079	0.101
H010	0.3084	0.2510	0.5735	0.101
H011	0.2752	0.1368	0.5398	0.101
